# Risk factors and protective factors of depression in older people 65+. A systematic review

**DOI:** 10.1371/journal.pone.0251326

**Published:** 2021-05-13

**Authors:** Alexander Maier, Steffi G. Riedel-Heller, Alexander Pabst, Melanie Luppa

**Affiliations:** Faculty of Medicine, Institute of Social Medicine, Occupational Health and Public Health, University of Leipzig, Leipzig, Germany; Cardiff University, UNITED KINGDOM

## Abstract

**Objectives:**

Identifying risk factors of depression can provide a better understanding of the disorder in older people. However, to minimize bias due to the influence of confounders and to detect reverse influence, a focus on longitudinal studies using multivariate analysis is required.

**Design:**

A systematic literature search was conducted by searching the databases MEDLINE, Cochrane, PsycINFO and Web of Science for all relevant articles published from January 2000 to the end of March 2020. The following inclusion criteria were used: prospective design, nationally or regionally representative sample, published in English or German, analyzed risk factors for depression of individuals 65+ identified by multivariate analysis, and provided validity of diagnostic instrument. All results of multivariate analysis were reported and summarized.

**Results:**

Thirty articles were identified. Heterogeneous results were found for education, female gender, self-rated health, cognitive impairment and older age, although significant in several studies. Findings hinted at a protective quality of physical activity. In terms of physical health, chronic disease and difficulty initiating sleep homogeneously increased risk of depression. Mobility impairment resulted as a risk factor in three studies. IADL impairment and vision impairment were mostly identified as significant risk factors. Alcohol consumption and smoking behavior yielded heterogenous results. Psychosocial factors were assessed similarly in multiple studies and yielded heterogenous results.

**Limitations:**

Research was limited to articles published in English or German. Length of follow up was not considered for the presentation of results. Adjustments for and inclusion of different variables in the studies may distort results.

**Conclusion:**

Our findings demonstrate the necessity of refined, more comparable assessment tools for evaluating potential risk factors.

## Introduction

Depression is frequent in the elderly population; meta-analyses find prevalence rates of depressive symptomatology to be 17.1% in individuals 75 years old and older and 19.5% in individuals 50 years old and older [1]. Several aspects call for investigating risk factors for depression in the old age separately. Firstly, potentially important risk factors, such as bereavement, social isolation, impairment and somatic diseases are more prevalent in older age [2]. Secondly investigation shows depression in older age being either a prodromal or risk factor of later dementia [3]. Thirdly, the prognosis of late-life depression appears to be worse than for younger age groups [4]. Additionally, depression in late life has been found to be severely underdiagnosed by primary care physicians [5]. Given that presence and persistence of depressive symptoms increases morbidity, leads to lower life quality, higher suicidal mortality, higher non-suicidal mortality (e.g. by enhancing the risk of cardiac mortality) [6], the problem is serious. Furthermore the persistence of depressive symptoms may constitute a burden to society by augmentation of mean annual direct costs [7]. Since therapy for depressive disorders in old age is effective, especially combined pharmacological therapy and psychotherapy [8], early detection of depressive disorders and commencement of suitable therapy for older aged people specifically, is important. Thus, knowledge of the risk factors of depression in older people may help to identify high risk groups to reduce risk factors and to establish personalized interventions [9]. Many studies have examined factors associated with depression and reviews have been conducted previously [10,11]. However, to make a statement about risk factors or protective factors for developing depression, it is necessary to focus on longitudinal studies. No current review summarizing these less-frequent studies after June 2001 exists to our knowledge. Therefore, we conducted a systematic review of longitudinal studies published in this century that examine the risk factors of depression in the elderly population over 65 years old by applying quality criteria in the selection process and incorporating the results in a conceptual framework. The aim of this review is to summarize protective factors and risk factors for the onset of depression in older people 65+ and to put the findings in context of previous literature on risk factors of late-life depression.

## Conceptual framework

**Fig 1:**
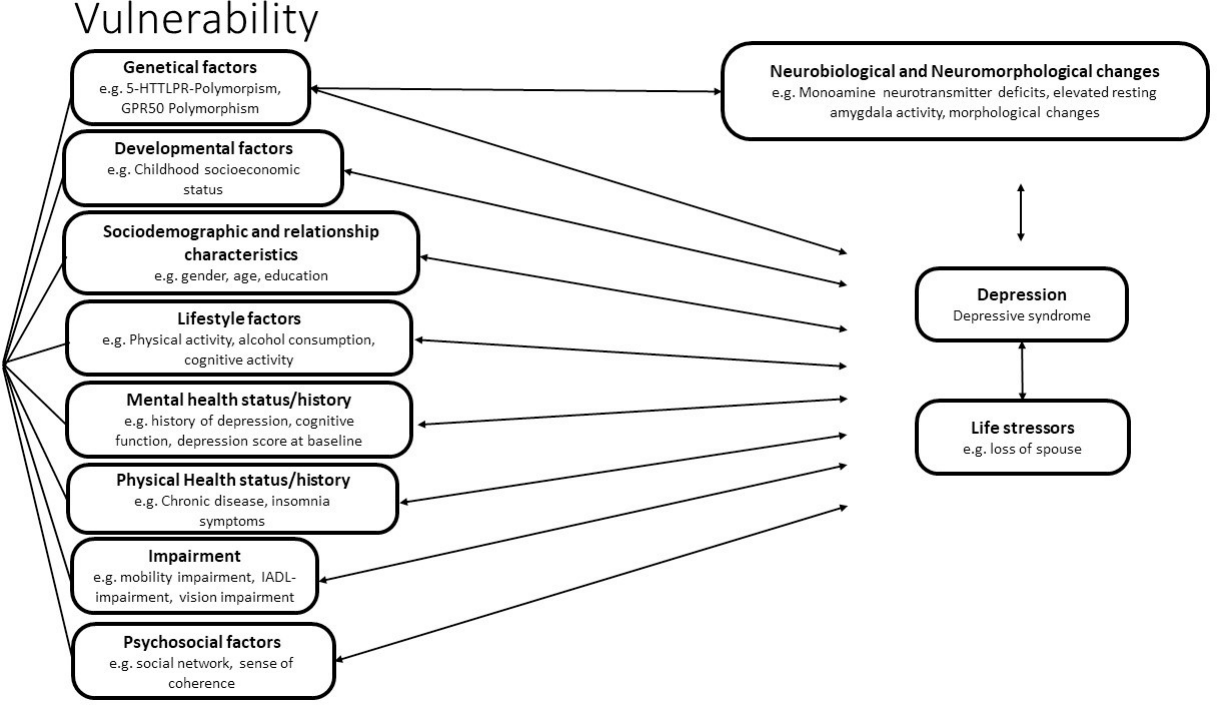
Conceptual framework of risk factors for incident depression.

In order to structure the results of the review, we developed a conceptual framework for risk factor for depression in the elderly population. We included new findings on neurotrophic theory for stress-related mood disorders [12,13], additional genetic and neurobiological factors such as GPR50 polymorphisms [14], associations of depression with morphometric brain-changes [15], and results of previous reviews that included cross-sectional studies [11] in our framework–the diathesis-stress model for mental disorders (see Fig 1). The diathesis-stress model suggests that the combination of stressful events (critical life events, stress) and the individual vulnerability are both preconditions for the development of a mental disorder. Vulnerability is determined by a set of factors differing from individual to individual. Psychological factors and factors associated with the personal development moderate the characteristics and the consequences of the mental disorder [16]. Therefore our combined model suggests that a mix of genetic, developmental, sociodemographic, and psychosocial factors, as well as relationship characteristics, physical and mental health status and impairment could potentially influence individual vulnerability to depression. These factors could moderate the effect of life stressors on the development of depression in subjects, or directly influence the development of depressive disorder. Furthermore, these factors could potentially be interrelated. Many potential risk factors may influence each other (e.g. health status variables such as history of stroke may influence impairment variables, age may influence health status variables). Taking this into consideration, a multivariate analysis is needed to effectively discover risk factors for depression. Furthermore depression could have a moderating effect on potential risk factors (e.g. depression may influence health status, as it leads to higher cardiac mortality) [17].

## Methods

### Literature research

**Fig 2:**
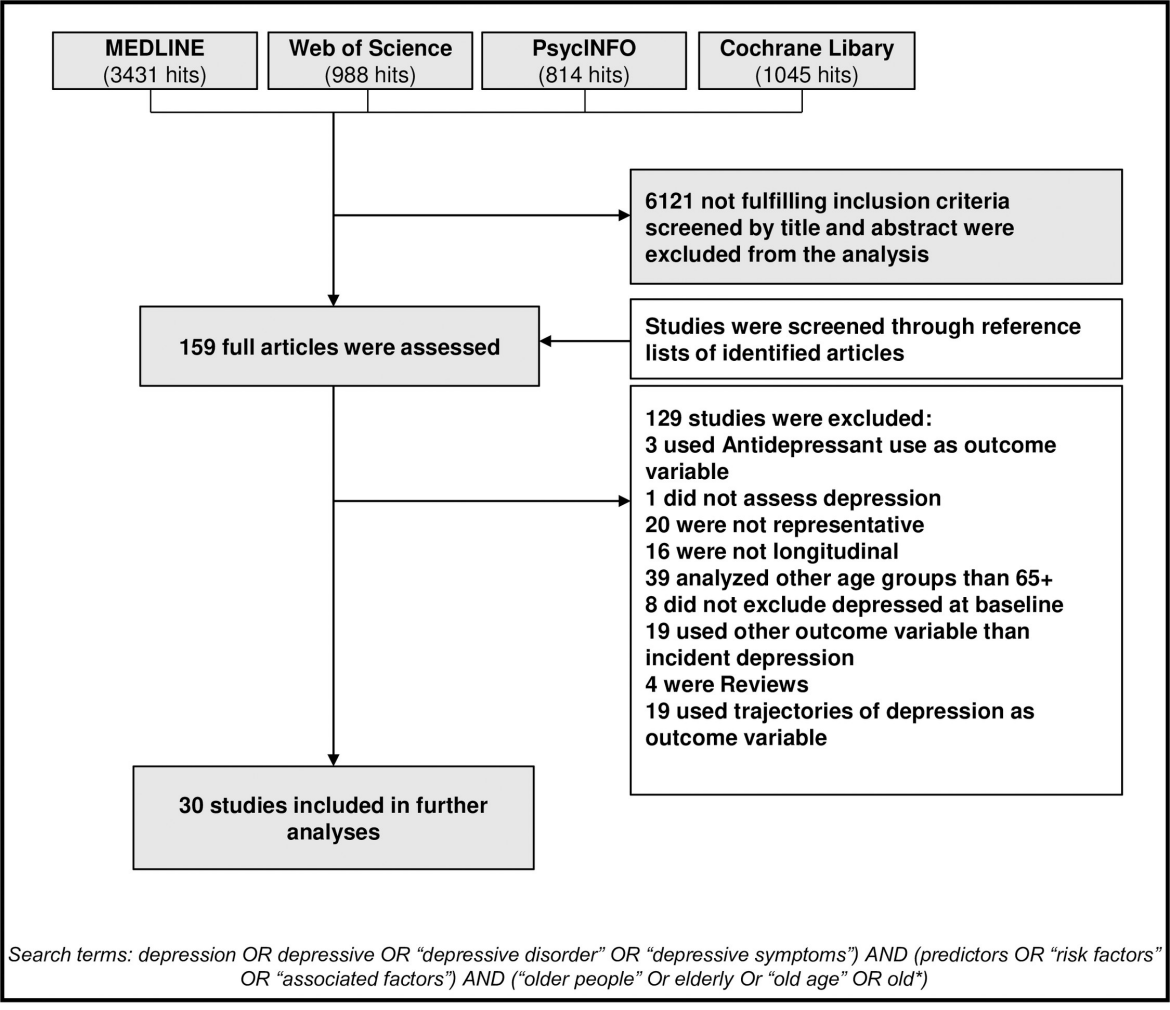
Results of the systematic literature search.

A systematic literature search was conducted. Relevant publications were identified by searching the electronic databases MEDLINE, Cochrane, PsycINFO and Web of Science applying the keywords: (depression OR depressive OR “depressive disorder” OR “depressive symptoms”) AND (predictors OR “risk factors” OR “associated factors”) AND (“older people” OR elderly OR “old age” OR old*) articles published from January 2000 to the end of March 2020. Articles published prior to June 2001 were summarized in a previous review with meta-analysis [10]. In the timespan from January 2000 to June 2001 we did not find any studies not covered in this previous review. Some additional studies were reported in the review due to differing inclusion criteria. For Cochrane, no additional limits were applied. In PsychInfo and Medline additional limits were applied: Languages: English, German, aged: 65+ years, methodology: prospective study, longitudinal study, follow-up study, systematic review, literature review, meta-analysis. As there were no other limits available in Web of Science, we added “AND (65- Or “65 and older”) AND (prospective OR longitudinal OR follow-up OR review OR meta-analysis) to the search. In addition, bibliographies of identified articles and reviews were searched for relevant articles. Of the 6278 identified articles, 157 were selected by title and abstract, 30 articles met the following inclusion criteria: prospective design, nationally or regionally representative sample, published in English or German, analyzing risk factors or protective factors for incident depression of individuals 65+, employed multivariate analysis, provided validity of diagnostic instrument (see Fig 2). Next, the quality of the 30 articles was rated using the criteria outlined in Table 1. All relevant data was extracted from reports independently.

**Table 1:** Criteria for assessing methodological quality.

A positive score of 1 applied if:
1) Study sample is nationally or regionally representative of the elderly population
2) Sample inclusion and/or exclusion criteria are formulated
3) Information on participants lost-to-follow-up is reported
4) The process of data collection is described (e.g. interview or self-report)
5) Training and quality control methods for interviewers’ technique are applied
6) Definition of the outcome criteria incident depression is provided: e.g. cut-off-score, measuring instrument for depression
7) Descriptive data are provided on depression: e.g. number of incident cases
8) Characteristics of study participants (socio-demographic, clinical, social) are given
9) For each variable of interest, sources of data and details of methods of assessment are given
10) Reliability and/or validity of study instruments is reported
11) Detailed description of statistical analysis is given
12) Adjustment for cognitive status in analyses is made (0 if no information is provided)
13) Individuals living with dementia are excluded from the analysis (0 if no information is provided)
14) Information on non-significant risk factor and protective factor variables is reported
15) Precision of estimates is given (e.g. 95% confidence interval)
16) model is adjusted for potentially relevant cofounders

Table 1 shows all criteria used for assessing the methodological quality of the studies about risk factors or protective factors for depression in individuals 65+. If one of the 17 criteria were met, 1 point was added. If the study did not meet one criterion, 0 points were added. Studies reaching 16 to 14 points were considered as “high quality”, studies reaching 13 to 11 points “medium quality” and studies reaching 10 points or less “lower quality”.

Factors were considered as significant risk factors if confidence intervals of Odds Ratio (OR) or Hazard Ratio (HR) was above 1. Factors were considered as significant protective factors if confidence intervals of OR or HR were below 1. All factors not fulfilling that criteria were considered as non-significant factors.

## Results

### Methodical characteristics

Methodical characteristics applied for significant risk factors are shown in Tables 2 and 3. The review included studies from Africa, Asia, North-America and Europe with studies form Nigeria, Japan, Taiwan, South Korea, USA, Austria, France, the Netherlands, Germany, Finland, Sweden, Great Britain, and a survey including data from several European countries. The shortest study was continued for 1 year and the longest for 12 years, mean time from baseline to last follow-up was 3.8 years. Length of interval between follow-ups, as well as number of follow-ups, can be seen in detail in Table 2. Sample size of participants included into analysis ranged between 115 and 17067 with most surveys analyzing data of 1400–3500 participants. Most included studies analyzed samples of elderly individuals aged 65+ and did not provide information about mean age. However, one study only analyzed elderly individuals aged 70+, several 75+, one 85+ and one a sample of Austrian elderly individuals aged 77–78 at baseline. Information on incidence rate per person years at risk of incident depression were rarely provided. Incident rates of non-depressed baseline participants ranged widely. The smallest percentage of participants with incident depression was found in a Japanese study with 7.5% in 1.25 years of follow-up and the highest was 31.4% in a study lasting 2.5 years including major, minor and subsyndromal depression. This is not surprising considering the differing criteria for incident depression and length of the studies.

**Table 2:** Characteristics of included studies.

Study; year; country of sample	N^5^	Age range/mean age in years at baseline	Interval/number of follow-ups	Diagnostic Instrument for Depression Incidence	Criteria for “incident depression”	Incident rate per 1000 person years (95% CI) or cumulative incidence	Incident cases	Depression in the past excluded (assessment of depression in the past)
Ibadan Study of Ageing; 2011; Nigeria [18]	892	65+	3.25/1	WHO Composite International Diagnostic Interview version 3 (CIDI.3) (DSM-IV) (by trained interviewers)	DSM-IV criteria for Major Depressive Disorder	104.3/1000 years at risk 34.5% of non-depressed at baseline	308	Yes
Ibadan Study of Ageing; 2018; Nigeria [19]	1394	65+	At 3, 4, 5 years	WHO Composite Diagnostic Interview (CIDI.3) (DSM-IV) (by trained interviewers)	DSM-IV criteria for Major Depressive Disorder	120.9/1000 person years at risk	464	Yes
Aichi Gerontological Evaluation Study (AGES) project; 2019; Japan [20]	3464	65+	4/1	GDS-15	GDS-15 score > = 5	14% of non-depressed at baseline	490	No
The Tsurugaya Project; 2005; Japan; [21]	475	70+	1/1	GDS-30	GDS-30 Score > = 11 or antidepressant users	11.6% of non-depressed participants at bl	55	No
The JAGES prospective cohort study; 2016; Japan [22]	10458	65+	3/1	GDS-15	GDS-15-score > = 5	13.9% of non-depressed at bl	1.435 (1.403–1.458)	no
Obu Study of Health Promotion in the elderly; 2018; Japan [23]	3106	65+/71.5	1.25/1	GDS-15	GDS-15-score > = 6	7.7% of non-depressed participants	239	Yes (interview)
Obu study of Health Promotion for the Elderly; 2015; Japan [24]	3025	65+/ 71.4	1,25/1	GDS-15	GDS-15-score > = 6	7.5% of non-depressed participants	226	Yes (Interview)
Obu study of Health Promotion for the Elderly; 2016; Japan [25]	3066	65+	1,25/1	GDS-15	GDS-15-score > = 6	7.6% of non-depressed participants at baseline	232	Yes (Interview)
Survey of Health and Living Status of the Elderly in Taiwan; 2010; Taiwan [26]	1487	65+/ 72.8	4/1	CES-D-10	CES-D-10-score> = 10	19.7% of non-depressed at baseline	293	No
Yang et al.; 2015; Taiwan [27]	1467	65+	4/1	CES-D-10	CES-D-10-score > = 10	14.6% of non-depressed participants at baseline	215	No
Kim et al 2006; South Korea [28]	521	65+	2,4/1	GMS-AGECAT	GMS-AGECAT confidence level > = 3	12.1% of non-depressed participants	63	No information
Lyness et al.; 2009; USA [29]	405	65+	1/4	SCID (DSM-IV)	DSM-IV criteria for episode of major depression	5.3% major depression of non-depressed at baseline	33	No
Health and Retirement Study; 2019; USA [30]	4914	75+	8/1	CES-D-8	CES-D-8 score > = 4	-	-	no
The Vienna Transdanube Aging study (VITA); 2009; Austria [31]	331	77–78	2,5/1	HAM-D GDS-short version DSM-IV	DSM-IV criteria for depressive episode	31% including MDD, subsyndromal and minor depression	86 including MDD, subsyndromal and minor depression	Yes
ESPRIT study of neuropsychiatric disorders in French elderly; 2010; France [32]	1131	65+	At 2, 4, 7 years	CES-D-20 MINI (DSM-IV)	DSM-IV criteria of major depression or CES-D-20> = 16	-	-	No, but adjusted for history of depression
ESPRIT study of neuropsychiatric disorders in French elderly; 2015; France [14]	415 (only women)	65+	12/1	CES-D-20 MINI (DSM-IV)	DSM-IV criteria for major depression or CES-D-20> = 16	-	-	no
The French Three City study; 2013; France; [33]	2307	65+	2, 4, 7, and 10 years	CES-D-20 MINI	DSM-IV criteria for major depressive episode or CES-D> = 20	22.6%	521	no
The French Three-City study; 2011; France [34]	3824	65+	2/2	CES-D (excluding “my sleep is restless”) MINI (history of major depression)	CES-D-scores > = 15 (“my sleep was restless”) excluded as item	16.2% of non-depressed participants	618	No
AMSTEL; 2000; Netherlands [35]	1940	65–84	3/1	GMS-AGECAT	GMS-AGECAT confidence level > = 3	15.9% of non-depressed at baseline	309	No
AMSTEL; 2006; Netherlands; [36]	1915	65–84	3/1	GMS-AGECAT	GMS-AGECAT confidence level > = 3	13.1% of non-depressed and without Generalized Anxiety Disorder at baseline	250	No
German Study on Ageing, Cognition, Dementia in Primary Care Patients (AgeCoDe Study); 2013; Germany [2]	2512	75-99/ 79.6	1.5/2	GDS-15	GDS-15 score > = 6	42,7. (38.0–47.9) per 1000 person years	92	No
LEILA 75+.; 2012; Germany [37]	1265	75–99; 81.5	1.5/5	CES-D-20	CES-D-20-score > = 23 points	34 (31–37) per 1000 person years	92	No
Evergreen Project; 2003; Finland [38]	384	65+	8/1	RBDI	RBDI-score > = 5	17% of non-depressed participants	66	No
GERDA Project; 2014; Finland [39]	115	85+	5/1	DSM-IV GDS-15 MADRS-30	Diagnosis of depression after joint evaluation of medical record, questionnaires, and interviews. Including major depressive disorder, dysthymic disorder (…)	25.5% of non-depressed participants	40	No
Kungsholmen project 2000; Sweden [40]	894	75+/84.5	3/1	DSM-IV	Depressive syndromes According to DSM-IV	8/1000 person years	29	no
English longitudinal study of the Ageing (ELSA); 2008; [41]	2929	65+	2/1	CES-D-8	CES-D-8-score > = 3	16.5% of non-depressed at baseline	469	No
English Longitudinal Study of the Ageing (ELSA);2007; England [42]	2814	65+	2/1	CES-D-8	CES-D-8-score > = 3	16.5% of non-depressed at baseline	464	No
Survey of Health, Ageing and Retirement in Europe (SHARE); 2019; Several Countries^6^ [43]	17067	65+	2/1	EURO-D-12 item	EURO-D-12-score > = 4	12.3% 6.62/100 person years	2,862	No
Nihon University Japanese Longitudinal Study of Aging: NUJLSOA; Japan [44]	3065	65+	3/1	CES-D-11	CES-D-11-score > = 7	Not given	Not given	No
Prospective community-based study of late-life psychiatric morbidity in Kwangju; South Korea [45]	792	65+	2/1	GMS-AGECAT	GMS-AGECAT confidence level > = 3	12.9%	102	No

ARR = Adjusted Risk Ratio BMI = Body Mass Index; BR = Binomial Regression; CPHR = Cox proportional hazard regression; CPHA = Cox Proportional Hazard Analysis; CRM = Cox Regression Model; DSM = Diagnostic and Statistical Manual of Mental Disorders; GDS = Geriatric Depression Scale; GLM: Generalized Linear Model; GLMLL: Generalized Linear Model with Logistic Link; GMSS = Geriatric Mental State Schedule; GMS-AGECAT = Automated Geriatric Examination for Computer Assisted Taxonomy- Geriatric Mental State Schedule; HAM-D = Hamilton Depression Rating Scale; HR = Hazard Ratio; IRR = Incident Risk Ratio; JAGES = Japan Gerontological Evaluation Study; Leila 75+ = Leipzig Longitudinal Study of the Ageing; LR = Logistic regression; MADRS = Montgomery-Åsberg Depression Scale; MCM = Multivariate Cox Model; MINI = Mini-International Neuropsychiatric Interview; MBLR = Multivariate Binary logistic regression; MLM = Mixed logistic model; MLR: Multiple logistic regression/Multivariate Logistic Regression; OR = Odds Ratio; RBDI = Finish modified version of Beck´s 13-item depression scale; RR = Relative Risk; SLR = Stepwise logistic regression; SMOLR = Stepwise Multiple Ordinal Logistic Regression; SCID = Structured Clinical Interview for DSM-IV; SLEs = Stressful life events.

^1^Structured Interview for Diagnosis of Dementia of Alzheimer Type, Multi-infarct Dementia and Dementia of Other Etiology.

^2^Activities of Daily Living

^3^Instrumental Activities of Daily Living by Lawton and Brody, 1969

^4^Social network index according to Wenger and Tucker 2002

^5^number of participants included into the analysis

^6^Denmark, Sweden, Switzerland, Luxembourg, Austria, Germany, Belgium, France, Slovenia, Czech Republic, Estonia, Spain, Italy, Israel.

**Table 3:** Results of included studies.

Study; year; country of sample	N^5^	Identified Risk factors or protective factors	Instruments measuring factors	Risk (95% CI) adjusted	Risk (95-% CI) unadjusted	Risk type	Type Of multivariate analysis	Quality rating; scores	Depression in the past excluded	Variables adjusted for
Ibadan Study of Ageing; 2011; Nigeria [18]	892	**Men** - **Women** Rural residence No Regular contact with friends	Rural classified as <12000 households CIDI	2.5 (1.4–4.4) 2.1 (1.1–3.7)	Unadjusted results not provided for men/women separately	OR	LR	High;14/16	yes	Age Sex
Ibadan Study of Ageing; 2018; Nigeria [19]	1394	**Men**Occupational attainment (reference: skilled) • Trade • Elementary **Women**Rural residenceNo regular contacts with family	Self-report, categorised based on International standard classification of occupations Rural classified as <12000 households CIDI	1.4 (1.0–2.0) 1.5 (1.1–2.1) 1.3 (1.0–1.7) 2.2 (1.0–4.7)	1.4(1.0–2.0) 1.5(1.1–2.1) 1.3(1.0–1.7) 2.2(1.0–4.7)	HR	CRM	High;14/16	yes	Age
Aichi Gerontological Evaluation Study (AGES) project; 2019; Japan [20]	3464	**Men:** 1 or more life events Age Poorer self-reported health ***Protective factors*:** Having hobbies Sense of coherence: medium Sense of coherence: high **Women:** 1 or more life events Age old-old (compared to young-old) **Protective factors** Sense of coherence: high Sense of coherence: no response	Question “did you experience any of the following events: (…)” 13-item Sense of Coherence scale (SOC-13) Question “did you experience any of the following events: (…)” 13-item Sense of Coherence scale (SOC-13)	1.64 (1.22–2.19) 1.33 (0.96–1.86) 1,92 (1.35–2.78) 0.59 (0,40–0,86) 0.58 (0.41–0.82) 0.24 (0.16–0.35( 1.49 (1.11–2.01) 1.55 (1.11–2.15) 0.35(0,23–0,52) 0.44 (0.26–0.75)	Unadjusted results not provided	OR	GLM	Medium;13/16	no	Frequency of meeting friends Emotional support: Receiving + providing Instrumental support Receiving + providing Hobbies (yes/no) Participation in organization Life events Illness Self-reported health IADL Sense of coherence Age Marital status Educational level Equivalent income (all variables dichotomous)
The Tsurugaya Project; 2005; Japan; [21]	475	Not having someone with whom one can consult in trouble Not having someone who can take care of you when you are ill in bed	Question: yes/no Question: yes/no	2.6 (1.2–5.3) 3.0 (1.4–6.1)	Results adjusted for sex, age: 2.8(1.5–5.2) 2.9(1.6–5.3)	OR	LR	High;14/16	no	Sex Age Presence/absence of spouse Numbers of people in household History of physical disease Age at finishing education Cognitive function Physical function Level of pain Self-rated health GDS-score at baseline
The JAGES prospective cohort study; 2016; Japan [22]	10458	Low Childhood Socioeconomic status Low Annual household income	Question to participants arranged at 5-point Likert scale Question: < 2 million yen	1.27 (1.08–1.50) 1.32 (1.08–1.60)	Adjusted for age and sex: 1.44(1.23–1.69) -	ARR	BR	Medium;12/16	no	Age Sex Childhood socioeconomic status Education (High, middle, low) Adult socioeconomic status: longest occupation (non-manual, manual, no occupation) Annual household income (high, middle low) Living situation (Own home, Rent home, Other) Disease status Health behaviours (smoking, alcohol, walking time) Social relationships (marital status, employment status, social participation, social support, general trust) Municipality of residence
Obu Study of Health Promotion in the elderly; 2018; Japan [23]	3106	**PROTECTIVE FACTORS** Light physical exercise Taking enrichment lessons Using personal computer Participation in events at the community Centre Attending a community meeting **Predictive Factors** No light physical exercise Not taking enrichment lessons Not using a personal computer No participation in events at the community Centre Not attending a community meeting	Question: yes/no Question: yes/no Question: yes/no Question: yes/no Question: yes/no Question: yes/no Question: yes/no Question: yes/no Question: yes/no Question: yes/no	0.74(0.56–0.98) 0.62 (0.46–0.85) 0.68 (0.48–0.97) 0.54 (0.40–0.72) 0.69 (0.52–0.92) 1.35 (1.02–1.79) 1.61 (1.18–2.17) 1.47 (1.03–2.08) 1.85 (1.39–2,50) 1.45 (1.09–1.92)	0.62 (0.48–0.81) 0.50 (0.38–0.67) 0.51 (0.37–0.69) 0.41 (0.31–0.54) 0.52 (0.39–0.67) 1.61 (1,23–2,08) 2.00(1.49–2.63) 1.96(1.44–2.70) 2.44(1,85–3,22) 1.92(1,49–2.56)	OR	MLR	High; 15/16	yes	Age, Gender, Education, Current smoking status, Alcohol consumption Living status Self-rated health Scores on MMSE Score on SPPB Total number of medication doses GDS at baseline
Obu study of Health Promotion for the Elderly; 2015; Japan [24]	3025	Poor self-rated general health Frailty	Question: “How good was your health” Limitations in physical tests in 5 Domains (mobility, strength, endurance, physical activity, weight loss)	1.86 (1.30–2.66) 1.86 (1.05–3.28)	Adjusted for Sex + Age: 3.27(2.35–4.55) Not given Unadjusted results not provided	OR	MLR	High; 15/16	yes	Age Sex Education Self-rated general health Fear of falling Smoking status Alcohol MMSE SPPB Frailty status GDS score at baseline
Obu study of Health Promotion for the Elderly; 2016; Japan [25]	3066	Gait speed slower than 1.0 m/s Sedentary behaviour time per day 240–480 minutes > = 480 minutes Using sleep medication	6.4 m walkway at participants usual gait speed, gait time measured between 2.0–4.4 m Self-report: International Activity Questionnaire Not specified	1.95 (1.25–3.04) 1.60 (1.09–2.38) 1.64 (1.02–2.64) 1.94 (1.40–2.67)	Adjusted for Sex + Age: 2.08(1.34–3.22) 1.59(1.08–2.34) 1.72(1.08–2.75) 1.98(1.44–2.72) Unadjusted results not given	OR	MLR	High;15/16	yes	Age Sex Educational history Current smoking status Current alcohol consumption Living arrangements Habit of going out General cognition Mobility Sedentary behaviour time Household and locomotive activities time
Survey of Health and Living Status of the Elderly in Taiwan; 2010; Taiwan [26]	1487	Female Sex **Change of variables between baseline and follow up as independent variables:** Worse perceived health stress Worse perceived financial stress Worse Life satisfaction Worse Functional condition	Self-rated scale Self-rated scale Life Satisfaction Index (LSI-A) ADL and IADL	1.58 (1.14–2.19) 3.06 (2.21–4.24) 2.02 (1.41–2.89) 1.92 (1.29–2.84) 2.39 (1.72–3.33)	Unadjusted results not provided	OR	Multivariate regression analysis	Medium; 13/16	no	Sex Age Education Marital status Ethnicity Occurence of new disease Perceived health stress Perceived financial stress Instrumental social support Emotional social support Life satisfaction Functional condition
Yang et al.; 2015; Taiwan [27]	1467	Ongoing heart disease Worsening ADL^2^ Worsening IADL Ongoing Arthritis or rheumatism	Not specified ADL-Scale Six items from older OARS IADL-survey Not specified	1.64(1.17–2.30) 1.80(1.28–2.52) 1.86(1.25–2.75) 1.50(1.08–2.09)	1.75(1.28–2.40) 2.08(1.55–2.79) 2.20(1.53–3.16) 1.67(1.22–2.29)	OR	LR	Medium; 12/16	no	Sex Age ADL change IADL chage Mobility change Perceived health status Comorbidities
Kim et al 2006; South Korea [28]	521	Pre-existing heart disease Lower HDL cholesterol	Self-reported Assay of blood sample	2.2 (1.3–3.7) 1.3 (1.1–1.6)	2.1(1.3–3.3) No information	OR	MLR	High;14/16	No information	Age Gender Education Level of disability
Lyness et al.; 2009; USA [29]	405	Minor or subsyndromal depression at baseline Physical Self-Maintenance Scale total score >0 History of major or minor depression Other psychiatric disorder	SCID (DSM-IV) Physical Self-Maintenance Scale (higher scale indicates poorer functioning) SCID (DSM-IV) SCID (DSM-IV) of any current alcohol-related or anxiety disorder	2.86 (1.33–6.15) 2.86 (1.19–6.84) 2.47 (1.12–5.44) 2.67 (1.22–5.86)	Unadjusted results not provided	IRR	GLMLG	Lower; 8/16	no	Not specified
Health and Retirement Study; 2019; USA [30]	4914	Insomnia symptoms (vs. no symptoms) 2 symptoms 3 symptoms 4 symptoms **Protective factors** White race/Caucasian (vs. black/African American, other) Participating in physical activity Years of education	Question: How often do you have trouble 1) Falling asleep 2) Waking up during night 3) Waking up to early 4) How often do you feel rested in the morning Question Question: yes or no No information	2.80 (1.48–5.32) 4.44 (2.42–8.15) 6.74 (3.70–12.29) 0.72 (0.54–0.98) 0.51 (0.37–0.71) 0.92 (0.89–0.96)	Unadjusted results not provided	HR	CPHA	Medium;13/16	no	Age Sex Race BMI Smoking Alcohol Physical activity Years of education
The Vienna Transdanube Aging study (VITA); 2009; Austria [31]	331	Score on Fuld Object Memory Evaluation (cognitive function) **Protective factors** “troubles with relatives”	Fuld Object Memory Evaluation	0.90 (0.88–0.99) 0.5 (0.28–0.89) p = 0.18	Unadjusted results not provided	OR	SMOLR	Medium; 11/16	yes	Not specified
ESPRIT study of neuropsychiatric disorders in French elderly; 2010; France [32]	1131	**MEN** Low LDL-C levels at bl **WOMEN** **-**	Venous blood sample, determined by Friedwald formula	1.98 (1.06–3.72)	Adjusted for educational level 1.90 (1.25–2.89)	HR	MCM	Medium; 12/16	No, but adjusted for history of depression	Age Education level Marital status Cognitive impairment BMI Mobility Ischemic pathologies Hypertension Diabetes Alcohol and tobacco intect Recent loss of appetite Apo” History of psychiatric disorder
ESPRIT study of neuropsychiatric disorders in French elderly; 2015; France [14]	415 (only women)	**WOMEN** Homozygotes for minor Allele of GPR50-Polymorphism rs561077:AA	Buccal samples, Genotyping by LGC Genomics	1.77 (1.18–2.67)	Unadjusted results not provided	HR	CPHA with delayed entry	Medium;12/16	no	Age Education MMSE Incapacities, Cardiovascular ischemic pathologies Current anxiety disorders
The French Three City study; 2013; France; [33]	2307	2-year decrease in distance visual function	Self-report; switching between, from without difficulties to at least “with difficulties” or from “with difficulties” to “unable” from baseline to 2 year follow-up	3.03 (1.75–5.23)	Unadjusted results not provided	OR	MLM	High; 14/16	no	Study Centre Age Gender Time since baseline Income Living alone Ischemic pathologies Diabetes Respiratory diseases Number of medications Obesity Mobility impairment Cognitive impairment falls
The French Three-City study; 2011; France [34]	3824	Insomnia symptoms Sleep quality • Average • Poor Difficulty initiating sleep • Frequently • Often Difficulty maintaining sleep • Frequently • Often Early Morning Awakening • Rarely • Frequently • Often Number of Insomnia symptoms • 2 • 3–4 Prescribed sleep medication	Face-to face interview; sleep-questionnaire Self-report + control of medication/recipe by interviewer/ drug inventory	1.27 (1.05–1.54) 1.62 (1.32–1.98) 1.71(1.26–2.32) 1.65(1.19–2.28) 1.88(1.35–2.62) 1.63(1.01–2.62) 1.92(1.18–3.13) 1.31(1.01–1.70) 1.55(1.14–2.09) 1.58(1.16–2.15) 1.56(1.15–2.11) 1.75(1.28–2.40) 1.71(1.33–2.20)	1.81 (1.52–2.16) 2.14 (1.77–2.58) 3.16(2.40–4.14) 2.33 (1.74–3.12) 3.56(2.64–4.81) 1.96(1.25–3.07) 2.66(1.68–4.22) 1.155(1.26–1.90) 2.22(1.67–2.94) 3.17(2.06–4.88) 2.24(1.70–2.96) 3.03 (2.28–4.02) Unadjusted result not provided	OR	LR	High;14/16	no	Study Centre CES-D at baseline Gender Age Education Living alone Coffee consumption Alcohol consumption Smoking Chronic disease Past Major depression Disability Prescribed sleep medication intake Homeopathic and non-prescription treatments for sleep
AMSTEL; 2000; Netherlands [35]	1940	Loss of spouse Personal history of disorder IADL decrease (>1pt) Baseline IADL disability New chronic disease Baseline chronic disease	CAMDEX-interview IADL IADL not specified not specified	3.11 (2.10–4.60) 1.75 (1.26–2.43) 1.71 (1.28–2.27) 1.44 (1.10–1.90) 1.41 (1.05–1.90) 1.40 (1.08–1.80)	2.30(1.19–1.80) 1.61(1.25–2.06) 1.73(1.39–2.14) 1.55(1.26–1.92) 1.40(1.11–1.77) 1.46(1.19–1.80)	RR	SLR	Medium;14/16	no	Age Sex Education social support Personal history of depression Family history of depression Chronic diseases ADL disability IADL disability MMSE<26 Anxiety syndrome New organic syndrome New Anxiety syndrome *Changes between bl and follow-up*: Partner died Relocation All ADL decrease All IADL decrease New chronic diseases
AMSTEL; 2006; Netherlands; [36]	1915	Loss of spouse Recent IADL decrease Baseline IADL disability Chronic illness at bl	Interview IADL IADL interview	2.93 (1.93–4.47) 1.53 (1.12–2.10) 1.78 (1.28–2.48) 1.45 (1.10–1.91)	Unadjusted results not provided	OR	MLR	High;15/16	no	Age Sex Education Marital status Social support Personal history of depression Family history of psychiatric disorder Baseline chronic diseases Baseline ADL disability Baseline IADL disability Low MMSE(<26) Life events
German Study on Ageing, Cognition, Dementia in Primary Care Patients (AgeCoDe Study); 2013; Germany [2]	2512	Age (85+) Mobility impairment Vision impairment MCI Subjective memory impairment Current smoking	SIDAM-ADL-Scale^1^ SIDAM-ADL-Scale^1^ Consensus criteria by the International Working Group on mild cognitive Impairment Question Self-report	1.83 (1.24–2.70) 2.53 (1.97–3.25) 1.41 (1.04–1.91) 1.52 (1.10–2.10) 1.33 (1.01–1.74) 1.69 (1.13–2.53)	2.11(1.47–3.03) 2.91(2.31–3.66) 1.65(1.23–2.21) 1.68(1.25–2.24) 1.54(1.20–1.96) 1.69(1.13–2.53)	HR	CPHR	High;16/16	no	Sex Age Living alone Marital status Level of education Mobility impairment Vision impairment Hearing impairment IADL impairment Somatic comorbidity Mild cognitive impairment Subjective memory impairment Current alcohol consumption Current smoking apoE4
LEILA 75+.; 2012; Germany [37]	1265	Female gender Satisfactory Self-rated health status (Functional Impairment) Poor/very poor Self-rated health status (Functional Impairment) Stroke in the past (comorbidity) Risky alcohol consumption Higher specialist visits **Protective factors** Higher social network score, pre point	IADL^3^ 26 Item IADL^3^ 26 Item Question Self-report: g/day (= 20g w, 30g m) Self-report last 12 months Social Network Index, determined by the authors	2.93 (1.50–5.73) 2.60 (1.31–5.14) 2.64 (1.28–5.46) 2.78 (1.27–6.09) 2.33 (1.09–4.96) 1.61 (1.03–2.52) 0.84 (0.74–0.95)	Unadjusted results not provided	HR	CPHR	High;14/16	no	Age Gender Educational level Marital status Living situation (ref. alone) Self-rated health status ADL MMSE Myocardial infarction Stroke Specialist visits in last 12 months Hospitalization in last 12 months Stressful life events Social network score Alcohol consumption Family history of mental illness (Impatient treatment of near relatives, suicide of relatives)
Evergreen Project; 2003; Finland [38]	384	Age		1.09(1.03–1.16)	Unadjusted results not provided	OR	LR	High;14/16	no	Mobilty groups Physical activity groups Gender Age Number of chronic illnesses Length of education
GERDA Project; 2014; Finland [39]	115	Hypertension History of stroke GDS-15 Score at baseline	RR > = 160/95mmHg after 5min of Rest or treatment/previous diagnosis of hypertension Medical records, report of patients/relatives GDS-15	2.83 (1.08–7.42) 3.25 (1.12–9.44) 1.39 (1.09–1.76)	Unadjusted results not provided	OR	MLR	Medium;11/16	no	Age Gender Poor self-rated health Taking Anxiolytics History of stroke Delirium during preceding month Hypertension Impaired hearing GDS-score at bl Number of medications
Kungsholmen project 2000; Sweden [40]	894	History of depression/anxiety	Medical records, examination of participants	4.8 (1.7–7.9)	Unadjusted results not provided	OR	LR	Medium;12/16	no	Gender Age>85 Education <8 years Being immigrant Marriage status Being institutionalised Using home care Somatic illness Dementia Not feeling well History of depression/anxiety History of psychosis Disabilitiees in daily life Hearing disabilities Visual impairment No regular visitors Having no friends Being unsatisfied with social network
English longitudinal study of the Ageing (ELSA); 2008; [41]	2929	Visual impairment Older Age Female sex No. of illnesses Mobility impairment IADL-Impairment Current Smoker Family negative interaction	Self-rated scale Interview number of medical conditions in eight areas Interview Interview IADL-impairment Interview Interview 3-Items	1.66 (1.21–2.27) 1.30 (1.09–1.55) 1.42 (1.11–1.81) 1.18 (1.06–1.32) 1.48 (1.14–1.93) 1.52 (1.06–2.12) 1.50 (1.06–2.12) 1.14 (1.08–1.21)	Unadjusted results not provided	OR	MR	Medium;11/16	no	Visual impairment Hearing impairment Both visual and hearing impairment Age Sex Marriage status Education Working status Income Number of medical conditions Mobility impairment ADL impairment IADL impairment Ex-smoker Current smoker Alcohol user Family support Family negative interactions
English Longitudinal Study of the Ageing (ELSA);2007; England [42]	2814	Older Age Female Gender Poor sight Mobility disability IADL disability Current smoking Negative interaction with family Pain	Interview: fair, poor, or legally blind Self-reported Difficulty in at least 1 of 5 activities Self-reported Difficulty in at least 1 of 7 IADL activities Self-reported, 3-Item Score Question, moderate or severe pain	1.35 (1.13–1.61) 1.35 (1.06–1.71) 1.46 (1.12–1.90) 1.35 (1.03–1.78) 1.45 (1.10–1.91) 1.43 (1.03–1.96) 1.13 (1.07–1.20) 1.54 (1.19–2.00)	Unadjusted results not provided	OR	LR	Medium;12/16	no	Age Gender Education Pain Poor sight Mobility disability IADL disability Current smokers Negative interaction with family Marriage status Working status Heart disease Diabetes, stroke Lung disease Bone disease Cancer ADL disability Two measures of social networks for family income
Survey of Health, Ageing and Retirement in Europe (SHARE); 2019; Several Countries^6^ [43]	17067	Female gender Poor self-rated health Loneliness Older Age ADL-impairment Financial difficulty Cognition (<15) Chronic diseases (> = 2) Worse Education	Subjective rating of health Short loneliness scale ADL impairment in > = 1 interview Specific items from SHARE Project assessed: immediate recall, delayed recall, subtraction calculation skills, verbal fluency Heart disease, hypertension, cholesterol, stroke, diabetes, chronic lung disease, cancer, ulcer, Parkinson, fractures, dementia Years of Schooling: Less than 10 years	**99.9% CI** 1.78 (1.77–1.78) 1.67 (1.66–1.67) 1.63 (1.62–1.64) 1.44 (1.43–1.44) 1.34 (1.34–1.35) 1.30 (1.30–1.31) 1.27 (1.27–2.28) 1.24 (1.23–1.24) 1.09 (1.09–1.10)	Unadjusted results not provided.	OR	MBLR	High;14/16	no	Gender Self-rated health Loneliness Age ADL impairment Financial difficulty Cognition Chronic disease Education
Nihon University Japanese Longitudinal Study of Aging: NUJLSOA; Japan [44]	3065	Sleep disturbances: Difficulty initiating sleep Psychological stress Poor Self-Rated health	Self-reportet response to question (yes/no) Self-report (Do you have psychological stress? Yes/no answer) Self-report (How do you rate your present general health condition? Excellent/good/fair/poor/very poor)	1.592 (1.012–2.504) 1.553 (1.125–2.145) 2.517 (1.778–3.562)	2.042 (1.391–2.997) 1.846 (1.375–2.479) 2.589 (1.881–3.563)	OR	MLR	Medium; 12/16	no	Age Gender Educational history Place of residence Sleep duration Excessive daily sleepiness Discomfort feeling in the legs Subjective sleep sufficiency Psychological stress Self-rated health ADL
Prospective community-based study of late-life psychiatric morbidity in Kwangju; South Korea [45]	792	Insomnia	According to answers to questions: Difficulty in initiation or maintenance of sleep with a frequency of 3 nights or more per week.	1.8 (1.2–2.9)	1.7 (1.1–2.7)	OR	LR	Medium 13/16	no	Age Gender Education Housing Past occupation Current employment Living area Life events Social deficit Physical activity GMS organicity GMS anxiety Daily drinking

ARR = Adjusted Risk Ratio BMI = Body Mass Index; BR = Binomial Regression; CPHR = Cox proportional hazard regression; CPHA = Cox Proportional Hazard Analysis; CRM = Cox Regression Model; DSM = Diagnostic and Statistical Manual of Mental Disorders; GDS = Geriatric Depression Scale; GLM: Generalized Linear Model; GLMLL: Generalized Linear Model with Logistic Link; GMSS = Geriatric Mental State Schedule; GMS-AGECAT = Automated Geriatric Examination for Computer Assisted Taxonomy- Geriatric Mental State Schedule; HAM-D = Hamilton Depression Rating Scale; HR = Hazard Ratio; IRR = Incident Risk Ratio; JAGES = Japan Gerontological Evaluation Study; Leila 75+ = Leipzig Longitudinal Study of the Ageing; LR = Logistic regression; MADRS = Montgomery-Åsberg Depression Scale; MCM = Multivariate Cox Model; MINI = Mini-International Neuropsychiatric Interview; MBLR = Multivariate Binary logistic regression; MLM = Mixed logistic model; MLR: Multiple logistic regression/Multivariate Logistic Regression; MMSE = Mini Mental Status Examination; OR = Odds Ratio; RBDI = Finish modified version of Beck´s 13-item depression scale; RR = Relative Risk; SLR = Stepwise logistic regression; SMOLR = Stepwise Multiple Ordinal Logistic Regression; SCID = Structured Clinical Interview for DSM-IV; SLEs = Stressful life events; SPPB = Short Physical Performance Battery.

^1^Structured Interview for Diagnosis of Dementia of Alzheimer Type, Multi-infarct Dementia and Dementia of Other Etiology.

^2^Activities of Daily Living

^3^Instrumental Activities of Daily Living by Lawton and Brody, 1969

^4^Social network index according to Wenger and Tucker 2002

^5^number of participants included into the analysis

^6^Denmark, Sweden, Switzerland, Luxembourg, Austria, Germany, Belgium, France, Slovenia, Czech Republic, Estonia, Spain, Italy, Israel.

Various instruments for assessing the dependent variable “incident depression” were applied. However, all studies excluded depression at baseline according to outcome criteria. Studies with dimensional criteria for depression included: versions of the Geriatric Depression Scale (GDS), Center of Epidemiologic Studies Depression Scale (CES-D), Beck´s 13-item depression scale with cut-off set at four (less rated as no symptoms), and the EURO-D scale with cut-off set at four. Three studies applied the GMS-AGECAT system, all using the recommended GMS-AGECAT level three or higher [46] for definition of incident depression. In two surveys on a French three city study, the Mini Neuropsychiatric Interview (MINI) and CES-D-20 with a cut-off at 16 were used. One study used the Structured Clinical interview for DSM-IV (SCID), using incident depressive episodes as positive outcome. Another study used depressive syndromes according to DSM-IV criteria as an outcome variable. [18] and [19] also defined the diagnosis of major depressive disorder according to DSM-IV as outcome variable. In another study case definition of depressive disorder included major depressive disorder, dysthymic disorder, substance induced disorder with depressive features, mood disorder with depressive features due to a general condition and minor depression diagnosed after joint evaluation of medical record data, earlier depressive disorder with ongoing treatment. Assessment tools included the Geriatric Depression Scale (GDS-15), Montgomery-Åsberg Depression Scale (MADRS), Organic Brain Syndrome (OBS) scale and the Philadelphia Geriatric Center Morale (PGCM) scale [39]. Another study defined subsyndromal, minor or major depressive episode as positive outcome according to DSM-IV criteria, as well as the Hamilton Rating Scale for Depression and GDS [31].

Psychosocial factors were assessed with a wide range of instruments. Discrepancies between instruments employed in various studies are mentioned in detail later. Factors associated with physical health status also differed widely between studies and are discussed in the results of physical health status. Activities of daily living (ADL) and impairment of activities of daily living (IADL) were measured with IADL and ADL scoring instruments which defined a specific number of impaired activities as an “impairment” (e.g. Forsell 2000 [40]). Not all papers clearly defined impairment [41,42].

### Methodical quality

The quality of studies included was assessed using criteria shown in Table 1 based on established criteria applied in previous reviews [47,48]. We adjusted the criteria of Luppa et al. [48] and added the criteria “individuals living with dementia are excluded from the analysis” on account of the potential overlap between symptoms of depression and dementia [49]. Furthermore, we added the criterium “model is adjusted for potentially relevant cofounders” to evaluate potential bias in studies for confounding. According to the criteria, 14 studies were rated high quality (47%), 15 were rated medium quality (50%) and 1 paper was rated “low quality” (3%) (see Table 4). The mean quality score was 13.1 of a possible 17 points. Common methodical shortcomings were lack of information on applied training and quality control of interviewers, missing adjustment for cognitive state in multivariate analysis and not excluding demented participants from the analysis.

**Table 4:** Score of studies on each criterium for quality assessment score.

Study	1*	2*	3*	4*	5*	6*	7*	8*	9*	10*	11*	12*	13*	14*	15*	16*	Total
Chou et al. 2007[42]	1	1	1	1	1	1	1	0	1	0	1	0	0	1	1	1	12
Chou et al. 2007[41]	1	1	1	1	1	1	1	1	1	0	1	0	0	0	1	0	11
Conde-Sala et. Al 2019 [43]	1	1	1	1	1	1	1	1	1	1	1	1	0	0	1	1	14
Dong et al. 2019 [30]	1	1	0	1	1	1	1	1	1	1	1	0	0	1	1	1	13
Forsell 2000 [40]	1	1	1	1	0	1	1	1	1	1	1	0	0	1	1	0	12
Gureje et al. 2011 [18]	1	1	1	1	1	1	1	1	1	1	1	0	1	1	1	0	14
Jaussent et al. 2011 [34]	1	1	1	1	0	1	1	1	1	1	1	0	1	1	1	1	14
Kim et al. 2006 [28]	1	1	1	1	1	1	1	1	1	1	1	1	0	0	1	1	14
Kim et al. 2009 [45]	1	1	1	1	0	1	1	1	1	1	1	0	0	1	1	1	13
Koizumi et al. 2005 [21]	1	1	1	1	0	1	1	1	1	0	1	1	1	1	1	1	14
Lampinen et al. 2003 [38]	1	1	1	1	1	1	1	1	1	1	1	0	0	1	1	1	14
Lue et al. 2010 [26]	1	1	1	1	0	1	1	1	1	1	1	0	0	1	1	1	13
Luppa et al. 2012 [37]	1	1	1	1	1	1	0	0	1	1	1	1	1	1	1	1	14
Lyness et al. 2009 [29]	0	1	0	1	0	1	1	1	1	0	0	0	0	1	1	0	8
Makizako et al. 2015 [24]	1	1	1	1	0	1	1	1	1	1	1	1	1	1	1	1	15
Mossaheb et al. 2009 [31]	1	1	1	1	0	1	1	0	1	0	1	1	1	1	0	0	11
Petersson et al. 2014 [39]	1	1	1	1	0	1	1	1	1	0	1	0	0	0	1	1	11
Schoevers et al 2005 [36]	1	1	1	1	1	1	1	1	1	1	1	0	1	1	1	1	15
Schoevers et al. 2000 [35]	1	1	1	1	1	1	1	1	1	1	1	0	1	0	1	0	13
Tani et al. 2016 [22]	1	1	1	1	0	1	1	1	1	1	1	0	0	1	1	1	13
Tsutsumoto et al. 2016 [25]	1	1	1	1	0	1	1	1	1	1	1	1	1	1	1	1	15
Uemura et al. 2018[23]	1	1	1	1	0	1	1	1	1	1	1	1	1	1	1	1	15
Weyerer et al. 2013 [2]	1	1	1	1	1	1	1	1	1	1	1	1	1	1	1	1	16
Yang et al. 2015 [27]	1	1	0	0	0	1	1	1	1	1	1	0	1	1	1	1	12
Yokohama et al. 2010 [44]	1	1	1	1	0	1	0	1	1	1	1	0	0	1	1	1	12
Misawa et al. [20]	1	1	1	1	0	1	1	1	1	0	1	0	1	1	1	1	13
Ryan et al. 2015 [14]	1	1	0	1	1	1	0	1	1	0	1	1	0	1	1	1	12
Ojagbemi et al. 2018 [19]	1	1	1	1	1	1	1	1	1	1	1	0	1	1	1	0	14
Carrière et al. 2013 [33]	1	1	1	1	0	1	1	1	1	1	1	0	1	1	1	1	14
Ancelin et al. 2010 [32]	1	1	0	1	0	1	1	1	1	1	0	1	1	0	1	1	12

*Criteria in the columns

1) Study sample is nationally or regionally representative of the older population.

2) Sample inclusion and/or exclusion criteria are formulated.

3) Information on participants lost-to-follow-up is reported.

4) The process of data collection is described (e.g. interview or self-report).

5) Training and quality control methods for interviewers’ technique are applied.

6) Definition of the outcome criteria incident depression is provided: e.g. cut-off-score, measuring instrument for depression.

7) Descriptive data are provided on depression: e.g. number of incident cases.

8) Characteristics of study participants (socio-demographic, clinical, social) are given.

9) For each variable of interest, sources of data and details of methods of assessment are given.

10) Reliability and/or validity of study instruments is reported.

11) Detailed description of statistical analysis is given.

12) Adjustment for cognitive status in analyses is made (0 if no information is provided).

13) Individuals living with dementia are excluded from the analysis (0 if no information is provided).

14) Information on non-significant risk factor or protective factor variables is reported.

15) Precision of estimates is given (e.g. 95% confidence interval).

16) model is adjusted for potentially relevant cofounders.

1 = Criteria fulfilled; 0 = Criteria not fulfilled.

### Risk of bias assessment

To assess the risk of bias in all included studies, the main author evaluated the risk of bias in 6 different bias domains (study participation, study attrition, risk factor measurement, outcome measurement, study confounding and statistical analysis and reporting) applying the QUIPS (Quality in Prognosis Studies) tool [50]. Judgement for all included studies is listed in Table 5. All studies had moderate or high risk of bias in at least one domain. Elevated risk of bias in study analysis and reporting was scarce.

**Table 5:** Judgement of risk of bias in 6 domains applying QUIPS tool.

Study	Risk of Bias in Study Participation	Risk of Bias in Study Attrition	Risk of Bias in Risk Factor Measurement	Risk of Bias in Outcome Measurement	Risk of Bias in Study Confounding	Risk of Bias in Study Analysis and Reporting
**Chou et al. 2007[42]**	low	moderate	high	high	low	low
**Chou et al. 2007[41]**	low	moderate	low	low	low	low
**Conde-Sala et. al 2019 [43]**	low	high	low	low	moderate	low
**Dong et al. 2019 [30]**	high	high	moderate	moderate	high	low
**Forsell 2000 [40]**	high	high	low	high	moderate	low
**Gureje et al. 2011 [18]**	low	moderate	low	low	high	low
**Jaussent et al. 2011 [34]**	moderate	moderate	low	low	low	low
**Kim et al. 2006 [28]**	high	low	moderate	low	moderate	low
**Kim et al. 2009 [45]**	low	high	low	low	high	low
**Koizumi et al. 2005 [21]**	moderate	moderate	high	low	moderate	low
**Lampinen et al. 2003 [38]**	moderate	moderate	high	moderate	high	low
**Lue et al. 2010 [26]**	moderate	high	moderate	low	high	low
**Luppa et al. 2012 [37]**	low	moderate	low	low	low	low
**Lyness et al. 2009 [29]**	high	moderate	low	low	high	-
**Makizako et al. 2015 [24]**	high	moderate	low	low	moderate	low
**Mossaheb et al. 2009 [31]**	moderate	moderate	high	high	high	high
**Petersson et al. 2014 [39]**	low	moderate	low	high	low	moderate
**Schoevers et al 2005 [36]**	moderate	moderate	low	low	moderate	low
**Schoevers et al. 2000 [35]**	moderate	moderate	low	low	moderate	moderate
**Tani et al. 2016 [22]**	low	moderate	moderate	low	moderate	low
**Tsutsumoto et al. 2016 [25]**	high	moderate	low	low	high	low
**Uemura et al. 2018[23]**	high	moderate	low	low	low	low
**Weyerer et al. 2013 [2]**	low	moderate	low	low	low	low
**Yang et al. 2015 [27]**	moderate	high	low	low	moderate	low
**Yokohama et al. 2010 [44]**	moderate	high	moderate	low	moderate	low
**Misawa et al. [20]**	high	moderate	moderate	low	moderate	low
**Ryan et al. 2015 [14]**	moderate	high	moderate	low	moderate	low
**Ojagbemi et al. 2018 [19]**	low	moderate	low	low	high	low
**Carrière et al. 2013 [33]**	moderate	moderate	low	low	high	low
**Ancelin et al. 2010 [32]**	low	moderate	moderate	low	moderate	low

Judgement of risk of bias by the main author in 6 domains applying the criteria of QUIPS tool [50].

### Risk factors and protective factors of incident depression

A list with of results of all potential risk factors analyzed in the included studies can be found in the appendix. Distinctions were made between high, medium, and low quality. A report of the number of significant risk or protective factors and insignificant results for all analyzed variables is provided.

#### Genetic factors

Genetic variations of serotonin-transporter-linked polymorphic region (5-HTTLPR) were analyzed in a study of Austrian older people and yielded no significant results for occurrence of 5-HTTLPR short allele [31]. A study of 415 older French women assessed GPR50 polymorphisms (melatonin-related receptor) located on the X-chromosome and found that homozygotes for the minor allele of *rs561077* were a risk factor for incident depression in women, but the polymorphisms *rs13440581* and *rs2072621* were not found to increase risk for depression [14]. A German study looked at Apolipoprotein E and compared having at least one 4-allele with having no 4-allele with insignificant results [2].

#### Developmental factors

Lower childhood socioeconomic status was identified as a positive risk factor in one study of medium quality [22].

#### Sociodemographic and relationship characteristics

Older age was identified as a risk factor in five studies [2,38,41–43] but was not significant in eleven studies [18,24,26,27,29,30,35–37,40,44]. Additionally, older age was insignificant in both men and women analyzed separately in one study [18] and increased risk for depression in women, but not in men in another [20]. Female gender was associated with more cases of incident depression in seven studies [18,26,37,41–44] but insignificant in ten studies [2,24,27,29,30,31,36,38–40]. Marital status was also assessed in ten studies [2,18,20,26,29,36,37,41–43], but did not reach significance. “Never being married” was also investigated by two studies [40,43] and found to be insignificant. In both high and medium quality studies significance and non-significance for the factor were results of the analyses. Also, living alone versus living with others did not reach significance in any of the studies investigating that factor [2,24,25,29,37]. Similarly, significant results were not found for living in an institution/nursing home [37], living in a rented home [22] or changing a living situation [31]. However, in Nigeria rural residence is a risk factor for depression in women, but not in men. These results were consistent in two studies of the same cohort [18,19]. Furthermore, rural residence was insignificant in a Japanese study. One Swedish study of 894 older people found use of care (home care), as well as use of institutionalized care to be insignificant [40]. Lower education was identified as a risk factor in four studies [30,41–43] but was insignificant in 12 others [19,20,22,24–26,29,31,36–38,40,44] and also in men and women individually. Noteworthy, only one study of high quality found education to be significant, whereas in five studies “lower education” was insignificant. One study found “middle level” of education, but not “high level” to be a protective factor against incident depression [2]. A Japanese study [22] reported “lower income” as a risk factor for incident depression, although and English study found that it was not significant [41]. Similarly, “lower income” was not a risk factor for both in men and women in another Japanese population [20]. Furthermore, economic status yielded no significant results for men and women in Nigeria [18,19]. Similarly, a number of factors asssociated with economic status, such as source of water supply and source of energy for cooking, did not reach significance in the same Nigerian cohort [19]. In terms of “longest held occupation”, there were no significant results when comparing manual, non-manual and no occupation [22]. A study of European older people found financial stress to be a significant risk factor [43] and a Taiwanese survey identified worsened financial stress as a significant risk factor [26]. Immigrant status was analyzed by one study, but did not increase the risk for depression [40]. Additionally, an American study found that having been raised in the USA was a protective factor for white people, [30] while a lower quality US-study reported contrary results [29].

#### Lifestyle factors

Participating in a physical activity was identified as a protective factor by one study of medium quality [30] However, a Finish sample of non-institutionalized seniors did not find a significant difference between the onset of depression in the subgroups disabled-sedentary, disabled-active, and mobile-sedentary as compared to a mobile-active subgroup in multivariate analysis [38]. In another study, some specific forms of physical activity, such as walking habits and moderate physical exercise, yielded insignificant results, however light physical exercise was found to be a protective factor [23]. Furthermore, household and locomotive activities time per day and habits of going out were not significant, although it was found that more than 240 minutes of sedentary time per day increased risk for incident depression in a study of 3066 Japanese older people [25]. The analysis of “current smoking” yielded varying results in studies of high- and medium quality: three surveys stated it to be a risk factor [2,41,42], whereas three studies found no significant association [24,25,30]. Additionally, being ex-smoker was insignificant in a study of older people in the U.K. [41]. Alcohol consumption was insignificant in all five studies analyzing the diversely defined factor [2,24,25,30,41]. However, a German study found at-risk drinking to be a risk factor [37]. A face-to-face interview study of Japanese older people identified taking enrichment lessons and using a personal computer as protective factor against incident depression, and yielded insignificant results for operating video or DVD-player [23].

#### Mental health status/history

Analysis of the history of mental health disorders revealed varying results. “History of mental disorder” as such was identified as a risk factor in one study [35]. A history of depression resulted as a risk factor in the only study rated “low quality” [29], but was not significant in an Austrian study of medium quality [31]. Analysis of “history of depression or anxiety” as a risk factor also yielded a significant association to more incident cases of depression in one survey [40], but was not significant in another [36]. A history of psychosis was not significant in one of these studies [40]. The study of low quality also yielded no significant results for “current alcohol related or anxiety disorder” at baseline as a risk factor [29]. Subsyndromal depression at baseline [18] and delirium in preceding month [39] also did not result in significant findings. Depression-score at baseline was identified as a risk factor in one study [39], although it was insignificant in another study of lower quality [29]. Family history of mental illness was not significant in all surveys that analyzed the factor [36,37]. In addition, despite frontal executive function tested with Trail Making Test Part B not being significant [31], poor cognitive function increased the risk of incident depression according to three studies [2,31,43], although four studies found no significant association [24,29,36,37]. Furthermore dementia at baseline did not increase risk for depression in two studies of medium and lower quality [29,40].

#### Physical health status

Various factors related to the presence of physical illnesses were analyzed with mostly insignificant results. Multivariate analysis of a cumulative illness score [29], having somatic illness [2,20,40], and having severe illness [31] all produced insignificant results. However, one survey identified “number of illnesses” as a risk factor [41], although the factor resulted insignificant in two other studies [2,38]. Equally, occurrence of new disease/new medical illness was not significant [26,36]. In contrast, poorer self-rated health was identified as a risk factor in four high-quality and medium-quality surveys [24,37,43,44] and only for men in another study [20]. Notwithstanding that in a study of medium quality [39] and lower quality [20,29], as well as separately for women [20] no significant results were found for this factor. Moreover, worsened self-rated health in a Taiwanese sample [27] was insignificant. Interestingly, “chronic disease” increased risk for depression significantly in all studies testing this factor for the whole sample [18,35,36,43], although no significant results were found in men and women separately. Likewise, new chronic disease [35] and the worsening of perceived health stress [26] were significant risk factors.

History of stroke/stroke in the past was a significant risk factor according to two studies [37,39], but “ongoing medical condition: stroke” was insignificant in another study [27]. Similarly, a new stroke in last 30 months was not a risk factor [31]. History of other specific somatic diseases was mostly insignificant. Likewise, history of myocardial infarction [37,39], history of lung disease, history of bone disease and history of cancer [42] did not reach significance. Other factors related to cardiac health such as myocardial infarction within last 30 months and coronary heart disease [31] remained insignificant. However, pre-existing heart disease was identified as a risk factor in a study [27,28], as was ongoing heart disease [27], but heart disease, defined as positively endorsing the question: “has your doctor ever told you, that you have (or had) any of the conditions on this card”, was insignificant in one study [42]. Furthermore, newly diagnosed cancer [31] and ongoing cancer [27] were not significant risk factors. In contrast, ongoing arthritis or rheumatism [27] increased risk of incident depression. More severe pain [42] and emergent pain [31] were identified as risk factors, although chronic pain yielded insignificant results for Nigerian men and women separately [18]. Illness of relatives was not a risk factor of incident depression [31]. With regard to vascular risk and depression, lower HDL cholesterol levels [28] and hypertension [39] were identified as a risk factors, although hypertension was not significant in another analysis [27] and low HDL-cholesterol was insignificant in older French women [32]. Surprisingly, low LDL-cholesterol increased risk of incident depression in French men [32]. Other factors related to vascular risk, such as another vascular risk factors not specified [31], diabetes [27,42], systolic blood pressure, diastolic blood pressure and higher BMI [30], were not significantly associated with incident depression.

Concerning medication, mean number of medications [39], taking anxiolytic medication [39], as well as taking antihypertensive medication [30] were all insignificant, whereas “using sleep medication” was a significant risk factor according to a high-quality survey [25]. Four studies analyzed the influence of sleep disturbances on depression. In a study of elders in the USA, analysis of a subsample of participants 75 years and older found two or more insomnia symptoms to be a risk factor of incident depression [30,44], as well as a Japanese study defining insomnia as difficulty in initiation or maintainance of sleep with a frequency of 3 night per week in the past month. Furthermore, difficulty initiating sleep and difficulty of maintaining sleep, but not poor sleep quality and early morning awakening, where significantly associated with depression onset [34]. A Japanese study found difficulty initiating sleep, but not difficulty maintaining sleep, early morning awakening, or excessive daytime sleepiness to be associated with depression onset [44].

A German study yielded no significant results for hospitalization during the last year [37], but found that two or more specialists visits in the last 12 months increased risk for depression [37].

#### Impairment

Measures of impairment where significantly associated with incident depression in several included longitudinal studies. Mobility impairment increased risk for incident depression significantly according to three studies [2,41,42]. Four studies found IADL impairment to increase risk for incident depression [35,36,41,42], although the factor did not reach significance in two other studies [2,29], nor in two further studies analyzing men and women separately [18,20]. Furthermore, ADL impairment was identified as a risk factor in one [43], but was insignificant in six studies [36,37,40–42,44]. Change of impairment was analyzed separately in some studies: worsened IADL impairment was identified as a risk factor in three studies [26,27,35,36], worsened mobility impairment was insignificant in one study [27], whereas worsened ADL- impairment was only significant in one [27] but not another study [36]. Visual impairment increased risk for depression as stated by three included publications [2,41,42], while results were insignificant in another survey [40]. In addition, a study of French older people found distance visual function loss and near visual impairment at baseline to be insignificant, but a 2-year decrease in distance visual function to be a risk factor [37]. Hearing impairment [2,39,41,40] and visual and hearing impairment analyzed together did not reach significance [41]. Physical frailty [24] and gait speed slower than one meter per second [25], but not fear of falling [25], increased risk for depression in two studies. From one study of Austrian older people, having a “handicap” was reported to be insignificant, without specifying the assessment method [31]. A study of 384 Finnish older people yielded no significant results for disabled sedentary vs. mobile active and disabled active vs. mobile-active groups [38] with mobility being assessed of self-reported ability to walk two kilometers and to be able to climb one flight of stairs without difficulty. One study included Instrumental Activities of Daily living score, Global Assessment of Functioning score and Karnofsky Performance Status scale into the analysis, all of which remained insignificant [29], although Physical self-maintenance score >0, indicating poorer functioning, was significant.

#### Psychosocial factors

The protective attribute of family support against depression onset was found to be significant in one study [41]. Furthermore family negative interaction was identified as a risk factor in two studies [41,42], although insignificant in another study of lower quality [29]. Per contra, frequency of contact by family and emotional support by family [42] yielded insignificant results. In addition, “no regular contact with family” was insignificant in Nigerian men and women individually in one study [18], but was a risk factor in women in another study analyzing the sample [19]. Receiving lower instrumental social support as assessed by the Duke Social Support Index [29] and worsened instrumental social support and worsening of received emotional social support as assessed by self-report using a five-point Likert-scale [26] revealed no significant results. A study analyzing older Japanese women and men separately, found receiving and providing emotional social support, as well as receiving and providing instrumental social support to be insignificant [20]. A higher sense of coherence was identified as a protective factor for both women and men, although a higher frequency of meeting with friends and having hobbies was protective for men, but not for women [20]. Furthermore, participation in organizations yielded no significant results in the same study [20]. Loneliness resulted as significantly increasing the risk of depression in one [43], but was not-significant in another survey [39]. In a study from northern Japan, negative answers to the questions: “Do you have someone with whom you can consult when in trouble?”, as well as “Do you have someone who can to take care of you when you are ill in bed” increased risk of depression significantly, although negative answers to the questions: “Do you have someone to take you to the hospital when you do not feel well?” and “Do you have someone with whom you can consult when in your physical condition is not good?” did not [21]. Higher social network score, indicating a more complex social network, assessed with a social network index in a German study [37], as well as participating in events in community center and attending a community meeting in Japanese older [23] were identified as protective factors against incident depression. In contrast, being called on for advice, having no regular visitors, having no friends and being unsatisfied with the social network did not significantly increased risk for depression in a Swedish sample [40]. Still, decreased life satisfaction was identified as a risk factor for depression [26]. Two studies analyzed social factors for incident depression separately for men and women. In a Japanese study, receiving and providing emotional and instrumental social support were not significant for both men and women; participation in organizations was also not significant [20]. In a Nigerian study, lack of regular contact with family was not found to be significant [18]. However, higher sense of coherence, as assessed by a 13-Item Sense of Coherence scale (SOC-13) was a protective factor for both men and women in Japan, while frequency of meeting with friends 1–2 times per week or more was identified as a protective factor for men, but not for women [20]. In contrast, the Nigerian survey found that having no regular contact with friends increased risk for depression in women, but not in men [18]. However, a later study of the same sample found significance in neither men nor women and additionally found no significant results for participation in family activities or participation in community activities [19]. Having hobbies yielded protective in men, but not in women in the Japanese study [20].

#### Life stressors

Stressful life events were analyzed as potential risk factors in four studies but did not yield significant results. However, having experienced stressful life events was defined differently in each study: [37] defined positive outcome as at least one event in last 6 months, [29] used a modified version of Louisville Older Persons Event Scale, [31] used a modified version of the Life Event and Difficulties Schedule by Brown and Harris, and [18] employed the List of Life Threatening Events 12 months prior to baseline for both men and women. A Japanese study analyzed factors separately for men and women and found a significant positive association of one or more stressful life events in the 12-months prior to the follow up for both men and women [20]. However, as life events were not assessed prior to baseline, this association cannot be interpreted as a risk factor. Two papers analyzing a sample in the Netherlands identified loss of spouse as a significant risk factor [35,36]. Furthermore the Austrian study found “bereavement” to be insignificant, but “troubles with relatives” to be protective against depression [31]. Participants affirming presence of psychological stress in their daily lifes also had a significantly higher incidence of depression in one study [44].

#### MRI alterations

One study examined MRI changes as potential risk factors, but yielded insignificant results for progression of white matter or periventricular hyperintensities in MRI, cella media index in MRI, and atrophy of medial temporal lobe in MRI [31].

## Discussion

This survey aimed to review all nationally or regionally representative studies analyzing risk factors of incident depression in longitudinal studies of older people 65 years of age or older using multivariate analysis. Compared to prior literature reviews, we focused on longitudinal studies which enables stronger statements for directionality of identified significant associations. Moreover, only including studies using multivariate analysis diminished the risk of confounders leading to falsely significant results. Most importantly, reporting non-significant results for the analyzed factors helped to avoid a false impression on certainty of risk factors, especially those that are analyzed frequently.

### Factors

#### Genetic factors

The insignificant result of 5-HTTPLR serotonin transporter promoter region short allele matches with results of a recent large meta-analysis focusing on a gene-environment interaction with 5-HTTPLR polymorphisms, stress and depression that found no significant interaction [51]. Research on GRP50 polymorphisms is scarce yielding mixed findings for connection between polymorphisms and mood disorder [52,53], although one included study in this review found a increase of risk by GPR50 polymorphism *rs561077* for incident depression which makes further research necessary. The APOE allele being insignificant in our findings supports the assumption of a previous study that found associations between ApOE4 alleles and depression might be due to confounding through individuals with Alzheimer’s disease [53], as demented patients were excluded and mild cognitive impairment was controlled for in the included study [2,11]. In addition, this result is consistent with previous longitudinal findings [11].

#### Developmental factors

Only one study analyzed self-rated childhood socioeconomic status finding it to increase risk for depression. Still, this result must be interpreted with precaution as recall bias might be high in this type of self-rated question concerning long past circumstances. Longer longitudinal studies assessing socioeconomic status objectively in childhood and adolescence are needed to produce more robust results for the relation to depression in late life.

#### Sociodemographic and relationship characteristics

Frequently analyzed factors rarely delivered homogenous results regardless of the quality of the involved studies. Findings on older age do not paint a clear picture, as twice the number of studies that identified older age as a risk factor, did not find a significant association. Gender also delivered heterogenous results, although male gender never increased risk for depression. In addition, lower education cannot be clearly stated as a risk factor, as it was more often insignificant than a risk factor, especially in high quality studies. In addition, a study of higher quality identifying middle-level, but not high-level education, as protective indicates that a simple dichotomous view on education might be oversimplified.

*Financial factors were assessed in several studies*. A study identifying the factor as risk factors used three subgroups according to income, with the lowest income group increasing risk for depression significantly [22] while a study analyzing total income in decile did not find a significant association [41]. Two other studies finding financial stress and worsened financial stress as risk factors might hint to lower income increasing risk for depression only when causing stress. Therefore, more research is needed for this set of factors. The identification of white ethnicity as a protective factor in one American study [30], but insignificant in study in New-York [29] might be due to the different methodological quality, or e.g. due to varying exposure to stressors in different communities. Marriage status seems to have no influence on depression onset, as it was frequently analyzed, but never significant.

#### Lifestyle factors

Physical activity was assessed with varying self-reported questions. Reporting more physical activity in some specific areas was protective against depression. In one study “participating in physical activity”, defined as self-report of mild, moderate, or vigorous activity (vs. no physical activity), was identified as a protective factor [29]. However in a study adding different subgroups of mobility-and physical activity, “physical active” was defined as walking at least several times per week as reported by the participants [38] leading to insignificant results. Overall, the variety in very specific factors assessed by self-report, concerning physical activity might explain the finding of protective quality of some factors while others remained insignificant. Further research with more comparable instruments is needed.

*Alcohol consumption was assessed diversely*. In three studies frequency of alcohol consumption was self-reported and rated ordinally [24,25,41], whereas one study used a simple dichotomous yes/no format [30]. Only one study used a question quantifying the average daily level of consumption on an ordinal level [2]. All these studies yielded insignificant results. In contrast, a study assessing “alcohol at risk drinking” dichotomizing the variable according to consumption below or over the level determined by the British Medical Association and found it to be a risk factor. These findings suggest, that frequency of drinking does not increase risk for depression. However, quantity of alcohol consumption led to contradicting results using different cut-offs and needs to be analyzed further. Findings on smoking were also heterogenous. Therefore, we suggest assessing current and past smoking habits more specifically to obtain clearer results. A study measuring “cognitive activity” revealed inhomogeneous results, with self-reported “taking enrichment lessons” and “using a personal computer” as protective factors, but “operating video or DVD player” as insignificant. The short activity “operating a video or DVD player” would logically usually precede the less cognitively challenging activity “watching video”, which might explain the lack of protective quality of this factor.

#### Mental health status

Whether a personal history of mental disorders increases risk of depression is difficult to interpret when the study [35] did not specify the definition of having a “history of mental disorders”. History of depression was only analyzed in two studies, leading to heterogenous results. Similarly, only half of the studies analyzing “history of depression” in a review of previous longitudinal studies could find a significant association [10]. Furthermore, depression score at baseline also led to heterogenous results. All other studied factors including subsyndromal depression at baseline, delirium in preceding month and history of psychosis did not increase risk of depression. Interestingly, our findings did not find a family history of mental illness to increase risk of depression and no previous survey has analyzed this factor longitudinally with multivariate analysis according to our knowledge [10,11]. Family history of mental illness was assessed by self-report and defined as inpatient treatment of mental disorders or suicide of a first-degree relative [37] or a question of family history of psychiatric illness [36]. The insignificant findings on family history of mental illness suits the findings of a previous cross-sectional twin study which provided hints that familiar history of depression might play a smaller role in depression onset in older rather than in younger subjects [54], although the sample of the study in question was much younger. Furthermore, these self-report questions might be vulnerable to information and recall bias. Cognitive function was frequently analyzed and found to be a significant risk factor, but results were heterogenous. This concurs with the results of five studies from a review of previous studies with participants 50+ or older [10] with similarly heterogenous results. The theory that realization of deteriorating cognitive function may lead to development of depressive symptoms secondary to cognitive decline was previously opposed [55]. However, it can nether be rejected nor supported due to de heterogenicity of our findings. Dementia did not increase risk of depression according to two of our included studies. Both studies use DSM-IV SCID to diagnose depression. However, the process of dementia might lead to symptoms that facilitate the diagnosis of depression such as hypersomnia, fatigue and weight loss. Therefore it is difficult to draw conclusions on the relationship between dementia and depression and the controversial topic is discussed for this reason inter alia [55].

#### Physical health status

Factors depicting present physical health status at baseline more generally, such as cumulative illness score and “having somatic illness” and “having severe illness” were mostly insignificant in our findings, although frequently analyzed. Only “number of illnesses” delivered heterogenous results. These findings are consistent with a previous meta-analysis, which found “poor-health status” to be insignificant, although studies used for the meta-analysis found the factor to be a risk factor for depression. However, our findings suggest, that chronic disease, as well as new chronic disease increased risk for depression. Similarly, nearly all studies from earlier reviews found “number of chronic health conditions” to be a risk factor [11]. Additionally, poorer and worsening of self-rated health did not deliver homogenous results, although it was significant in three studies. Considered together with two older longitudinal studies of participants 65+, finding poorer self-rated health to be a risk factor [10], these findings make further research necessary. History of stroke, but not new stroke seems to increase risk for depression, although stroke more indistinctly defined as “ongoing medical condition” of stroke [24] did not increase risk for depression. Interestingly, all four studies assessing the factor suspended the influence of the potential confounders ADL and/or IADL impairment by adding them to multivariate analysis. Results for heart disease as a risk factors do not allow a clear conclusion to be drawn. History of myocardial infarction and myocardial infarction in last 30 months were insignificant, whereas pre-existing heart disease yielded heterogenous results. The definition of heart disease differed between these studies: [56] used previous diagnosis with clear time of onset, whereas heart disease was assessed as a “ongoing medical condition(..)”; [27] and [42] defined heart disease as positive answer to the question, “Has a doctor ever told you that you have any of the conditions on this card?”. Thus, heterogeneity of the results might be caused by recall-bias in some of the studies. Vascular risk factors require further research, as lower HDL cholesterol levels and hypertension increased risk for depression significantly in single studies, but several other single studies did not find significant results for factors such as hypertension, BMI and blood pressure. Furthermore, taking specific medications seems to have no influence on depression onset, except for sleep medication. This supports our findings on insomnia symptoms, especially difficulty initiating sleep being associated with depression. However, the two studies analyzing difficulty maintaining sleep had heterogenous results. In the previous literature, two of four longitudinal studies of older people assessing insomnia symptoms yielded similar findings [10,11]. Increased pain seems to lead to higher risk of depression, although chronic pain, as such, does not. However, due to the small number of studies analyzing the influence of pain, more research is needed. In addition, the increase in risk through hospitalization and two or more specialists visit in the last 12 months was identified in a German population. Since contact to health care professionals could be an opportunity for prevention, if their predictive value results to be high, these findings call for further analysis.

#### Impairment

Impairment seems to increase the risk of incident depression, which is consistent with earlier reviews [10,11]. However, our findings make distinction between different measures of impairment necessary. Mobility impairment, IADL impairment, visual impairment, as well as worsening of IADL-impairment were frequently investigated and were found to be significant risk factors of depression in most studies. In contrast, less homogenous results for ADL impairment, or its worsening, hint to a lesser influence of this factor. In addition, our findings clearly suggest that hearing impairment does not increase the risk of depression.

#### Psychosocial factors

The varying methods and specificity of psychosocial factors in included studies makes interpretation difficult. Nearly all factors were assessed by positive or negative self-reported answer to a specific statement and were highly specific. Only “loneliness” and “family negative interaction” were assessed in more than one study and yielded heterogenous results. In addition, the findings of two studies hint to modest differences in the influence of social behavior in men and women. However, higher scores in social network measurement scales, such as the Sense of Coherence Scale (SOC-13) [20] as well as the more complex instrument, the Social Network Index [37] hint to a protective quality of these factors. Overall, more research on the influence of social factors with instruments validly depicting these complex systems is needed.

Our findings suggest the conclusion, that stressful life events, in general, do not increase the risk of depression independently. However, “loss of spouse” was identified as a risk factor. The reason why “troubles with relatives” resulted to be protective in a study of medium quality is unclear.

#### Reevaluation of the conceptual framework

Our findings allow a reevaluation of our conceptual framework. Genetic factors were only analyzed by single studies and therefore require further research. Furthermore, developmental factors such as childhood economic status require more research or might be available in longitudinal studies with younger age at baseline. Our findings could not unconditionally support commonly identified sociodemographic factors increasing risk of depression such as lower education, female gender and older age. In contrast, the frequently analyzed factor “marriage status” yielded homogeneously insignificant results. Concerning lifestyle factors, we found strong hints to a protective quality of some types of physical activity against incident depression, although further research with more comparable instruments is needed. Our heterogenous findings on drinking and smoking calls for assessment of frequency, quantity, and past consumption to obtain clearer results. Moreover, the protective effect of self-reported subtypes of cognitive activity only analyzed in one study calls for further investigation. Mental health status yielded heterogenous results on the influence of history of depression and cognitive function, whereas other factors, analyzed by single studies such as subsyndromal depression at baseline, delirium in preceding month and family history of depression remained insignificant. Apart from these factors, further research is needed for depression score at baseline, as previous diagnosis and depression questionnaires could potentially represent targets for screening. In factors related to physical health, chronic disease and insomnia symptoms had the clearest results to increase risk of depression with hints to a specific subset of insomnia symptoms having more influence. Factors related to stroke, heart disease, pain and vascular risk factors delivered heterogenous results and require further research. Our findings suggest a specification of the of impairment as a risk factor, as mobility, IADL- and visual impairment seem to increase the risk of depression, while we found less influence of ADL-impairment. Moreover, although frequently analyzed, hearing impairment apparently has no influence on incident of depression. Unfortunately, variety of methods for assessment of psychosocial factors does not allow a clear conclusion, although the results hint to a protective quality of a higher sense of coherence and a better social network. Studies analyzing factors concerning neurobiological and neuromorphological changes were scarce in this review, as only one study assessed MRI-alterations at baseline. These factors represent a wide and complicated field and are subjects of intensive research e.g. [15].

### Limitations

The research was limited to articles published in English or German. The systematic literature search was conducted by only one of the authors, which may have led to overlooking of some relevant studies. Similarly, limits applied as described before might have led to excluding relevant studies in the search. Length of follow up was stated, but not specifically considered for the presentation of results. The influence of varying methods for multivariate analysis and differing assessment methods for risk factors may have influenced the results. In the included studies, various types of multivariate analysis were applied, and the variables adjusted for differed greatly, as it can be recognized in Table 3. These substantial methodological differences strongly limited the comparability between results for specific factors of the studies and make finding sources of heterogeneity very difficult. However, by selecting only studies meeting our strict inclusion criteria and additionally applying quality criteria systematically, we attempted to achieve as much comparability between studies as possible. Furthermore, as shown in Table 2, only 6 included studies excluded depression in the past. Hence, some of the cases of “incident depression” might in fact represent a recurrence of depression. All studies had at least moderate risk of bias in one or more domain and many studies had several domains with high or moderate risk of bias. Therefore, the results presented in this review should be interpreted cautiously. Also, risk of bias results as well as heterogeneity between studies did not allow us to conduct a meta-analysis. In addition, we did not address the influence of differences in length of follow-up between the studies on their results, as the impact of this difference can only be speculated without a meta-analysis. The quality rating scale we applied merely supports judgement of relevance of certain results, although it does not affect any of the results.

## Conclusion

As depression is a common illness in older people and this age group is becoming increasingly important due to demographic change, identification of risk factors and protective factors for this mental disorder in older people is a highly relevant research topic. Our review allows for a better understanding of risk and protective factors by focusing on longitudinal studies using multivariate analysis. These factors can contribute to development of screening tools and interventions with the aim of improving health-related quality of life. Firstly, modifiable factors, such as physical activity, cognitive activity, social network and sense of coherence may represent a target for preventive intervention. Secondly, non-modifiable factors, such as genetic factors and impairment could be utilized to identify subgroups in which preventive interventions are cost effective. Thus, our findings demonstrate the necessity of further research with a focus on longitudinal studies using multivariate analysis and refined, more comparable assessment tools for risk factors of depression in older people.

## Supporting information

S1 Checklist PRISMA 2009 checklist predictors of depression. (DOC)

S1 Table All risk factors and protective factors of depression in older people 65+. (DOCX)

S1 File Risk of bias according to QUIPS in all studies. (DOCX)
