# Supplementary material for: Risk factors and protective factors of depression in older people 65+. A systematic review
Source: PLoS One. 2021 May 13;16(5):e0251326. doi: 10.1371/journal.pone.0251326 (PMC8118343; doi:10.1371/journal.pone.0251326)
Supplement: S1 Table — (DOCX) [file pone.0251326.s002.docx]

The table provides a detailed overview of the number of studies which yielded the respective factor as a significant predictor of depression (+), a protective factor against depression (-), or yielded an insignificant result for the respective factor (ns). Additionally, the number of studies of high, moderate and lower quality are described equally.

| Table 1: Risk factors and protective factors for depression in older people | | | | | | | | | | | | | | | | | | | | | |
| --- | --- | --- | --- | --- | --- | --- | --- | --- | --- | --- | --- | --- | --- | --- | --- | --- | --- | --- | --- | --- | --- |
|  | **All studies** | | | | | | **High quality studies** | | | | | | | **Moderate quality**  **studies** | | | | **Lower quality studies** | | | |
| **Predicting** | n | + | - | ns | n | | | + | | - | ns | n | | | + | - | ns | n | + | - | ns |
|  |  |  | | | |  | | |  | | | |  | |  | | |  |  | | |
| *Genetical factors* | | | | | | | | | | | | | | | | | | | | | |
| ApoE4: At least one 4 Allele | 1 | 0 | 0 | 1 | 1 | | | 0 | | 0 | 1 | 0 | | | 0 | 0 | 0 | 0 | 0 | 0 | 0 |
| 5-HTTPLR polymorphism: 5-HTTPLR short allele | 1 | 0 | 0 | 1 | 0 | | | 0 | | 0 | 0 | 1 | | | 0 | 0 | 1 | 0 | 0 | 0 | 0 |
| *Developmental factors* | | | | | | | | | | | | | | | | | | | | | |
| Childhood socioeconomic status: middle-low/low | 1 | 1 | 0 | 0 | 0 | | | 0 | | 0 | 0 | 1 | | | 1 | 0 | 0 | 0 | 0 | 0 | 0 |
| Childhood socioeconomic status: middle-high, high | 1 | 0 | 0 | 1 | 0 | | | 0 | | 0 | 0 | 1 | | | 0 | 0 | 1 | 0 | 0 | 0 | 0 |
| Childhood socioeconomic status: middle | 1 | 0 | 0 | 1 | 0 | | | 0 | | 0 | 0 | 1 | | | 0 | 0 | 1 | 0 | 0 | 0 | 0 |
| *Sociodemographic and relationship characteristics* | | | | | | | | | | | | | | | | | | | | | |
| Older age | 16 | 5 | 0 | 11 | 7 | | | 3 | | 0 | 4 | 8 | | | 2 | 0 | 6 | 1 | 0 | 0 | 1 |
| Male gender | 17 | 0 | 7 | 10 | 7 | | | 0 | | 3 | 4 | 9 | | | 0 | 4 | 5 | 1 | 0 | 0 | **1** |
| Female gender | 17 | 7 | 0 | 10 | 7 | | | 3 | | 0 | 4 | 9 | | | 4 | 0 | 5 | 1 | 0 | 0 | **1** |
| Marriage status: married | 8 | 0 | 0 | 8 | 4 | | | 0 | | 0 | 4 | 3 | | | 0 | 0 | 3 | 1 | 0 | 0 | **1** |
| Marriage status: widowed | 8 | 0 | 0 | 8 | 3 | | | 0 | | 0 | 3 | 4 | | | 0 | 0 | 4 | 1 | 0 | 0 | **1** |
| Marriage status: single | 5 | 0 | 0 | 5 | 2 | | | 0 | | 0 | 2 | 3 | | | 0 | 0 | 3 | 0 | 0 | 0 | **0** |
| Marriage status: divorced | 6 | 0 | 0 | 6 | 3 | | | 0 | | 0 | 3 | 3 | | | 0 | 0 | 3 | 0 | 0 | 0 | **0** |
| Never being married | 2 | 0 | 0 | 2 | 1 | | | 0 | | 0 | 1 | 1 | | | 0 | 0 | 1 | 0 | 0 | 0 | **0** |
| Having children | 1 | 0 | 0 | 1 | 0 | | | 0 | | 0 | 0 | 1 | | | 0 | 0 | 1 | 0 | 0 | 0 | **0** |
| Living situation: Alone | 5 | 0 | 0 | 5 | 4 | | | 0 | | 0 | 4 | 0 | | | 0 | 0 | 0 | 1 | 0 | 0 | **1** |
| Living situation: with others | 5 | 0 | 0 | 5 | 4 | | | 0 | | 0 | 4 | 0 | | | 0 | 0 | 0 | 1 | 0 | 0 | **1** |
| Living situation: Nursing home/Institutionalized | 1 | 0 | 0 | 1 | 1 | | | 0 | | 0 | 1 | 0 | | | 0 | 0 | 0 | 0 | 0 | 0 | **0** |
| Living situation: rented | 1 | 0 | 0 | 1 | 0 | | | 0 | | 0 | 0 | 1 | | | 0 | 0 | 1 | 0 | 0 | 0 | **0** |
| Change of living situation | 1 | 0 | 0 | 1 | 0 | | | 0 | | 0 | 0 | 1 | | | 0 | 0 | 1 | 0 | 0 | 0 | **0** |
| Use of care: Nursing home | 1 | 0 | 0 | 1 | 0 | | | 0 | | 0 | 0 | 1 | | | 0 | 0 | 1 | 0 | 0 | 0 | **0** |
| Use of care: home care | 1 | 0 | 0 | 1 | 0 | | | 0 | | 0 | 0 | 1 | | | 0 | 0 | 1 | 0 | 0 | 0 | **0** |
| Education: lower Education | 14 | 4 | 0 | 10 | 6 | | | 1 | | 0 | 5 | 7 | | | 3 | 0 | 4 | 1 | 0 | 0 | **1** |
| Education: higher Education | 14 | 0 | 4 | 10 | 6 | | | 0 | | 1 | 5 | 7 | | | 0 | 3 | 4 | 1 | 0 | 0 | **1** |
| Education: middle level (vs. high level; low level) | 1 | 0 | 1 | 0 | 1 | | | 0 | | 1 | 0 | 0 | | | 0 | 0 | 0 | 0 | 0 | 0 | **0** |
| Working status: in labor force | 2 | 0 | 0 | 2 | 0 | | | 0 | | 0 | 0 | 2 | | | 0 | 0 | 2 | 0 | 0 | 0 | **0** |
| Working status: retired | 2 | 0 | 0 | 2 | 0 | | | 0 | | 0 | 0 | 2 | | | 0 | 0 | 2 | 0 | 0 | 0 | **0** |
| Financial situation: Lower Income | 2 | 1 | 0 | 1 | 0 | | | 0 | | 0 | 0 | 2 | | | 1 | 0 | 1 | 0 | 0 | 0 | **0** |
| Longest occupation: manual vs. Non-manual; no occupation) | 1 | 0 | 0 | 1 | 0 | | | 0 | | 0 | 0 | 1 | | | 0 | 0 | 1 | 0 | 0 | 0 | **0** |
| Financial stresses/difficulties | 1 | 1 | 0 | 0 | 1 | | | 1 | | 0 | 0 | 0 | | | 0 | 0 | 0 | 0 | 0 | 0 | **0** |
| Worsened financial stresses | 1 | 1 | 0 | 0 | 0 | | | 0 | | 0 | 0 | 1 | | | 1 | 0 | 0 | 0 | 0 | 0 | **0** |
| Immigrant status | 1 | 0 | 0 | 1 | 0 | | | 0 | | 0 | 0 | 1 | | | 0 | 0 | 1 | 0 | 0 | 0 | **0** |
| Ethnicity: White/Caucasian | 2 | 0 | 1 | 1 | 0 | | | 0 | | 0 | 0 | 1 | | | 0 | 1 | 0 | 1 | 0 | 0 | **1** |
| *Lifestyle factors* | | | | | | | | | | | | | | | | | | | | | |
| Physical activity | 1 | 0 | 1 | 1 | 0 | | | 0 | | 0 | 0 | 1 | | | 0 | 1 | 0 | 0 | 0 | 0 | **0** |
| Walking habits | 1 | 0 | 0 | 1 | 1 | | | 0 | | 0 | 1 | 0 | | | 0 | 0 | 0 | 0 | 0 | 0 | **0** |
| Light physical exercise | 1 | 0 | 1 | 0 | 1 | | | 0 | | 1 | 0 | 0 | | | 0 | 0 | 0 | 0 | 0 | 0 | **0** |
| Moderate physical exercise | 1 | 0 | 0 | 1 | 1 | | | 0 | | 0 | 1 | 0 | | | 0 | 0 | 0 | 0 | 0 | 0 | **0** |
| Household and locomotive activities´ time | 1 | 0 | 0 | 1 | 1 | | | 0 | | 0 | 1 | 0 | | | 0 | 0 | 0 | 0 | 0 | 0 | **0** |
| Sedentary behavior per day: more than 240 min | 1 | 1 | 0 | 0 | 1 | | | 1 | | 0 | 0 | 0 | | | 0 | 0 | 0 | 0 | 0 | 0 | **0** |
| Habit of going out | 1 | 0 | 0 | 1 | 1 | | | 0 | | 0 | 1 | 0 | | | 0 | 0 | 0 | 0 | 0 | 0 | **0** |
| Current Smoking | 6 | 3 | 0 | 3 | 3 | | | 1 | | 0 | 2 | 3 | | | 2 | 0 | 1 | 0 | 0 | 0 | **0** |
| Ex-Smoker | 1 | 0 | 0 | 1 | 0 | | | 0 | | 0 | 0 | 1 | | | 0 | 0 | 1 | 0 | 0 | 0 | **0** |
| Alcohol consumption | 5 | 0 | 0 | 5 | 3 | | | 0 | | 0 | 3 | 2 | | | 0 | 0 | 2 | 0 | 0 | 0 | **0** |
| Alcohol at risk drinking | 1 | 1 | 0 | 0 | 1 | | | 1 | | 0 | 0 | 0 | | | 0 | 0 | 0 | 0 | 0 | 0 | **0** |
| Taking enrichment lessons | 1 | 0 | 1 | 0 | 1 | | | 0 | | 1 | 0 | 0 | | | 0 | 0 | 0 | 0 | 0 | 0 | **0** |
| Operating video or DVD player | 1 | 0 | 0 | 1 | 1 | | | 0 | | 0 | 1 | 0 | | | 0 | 0 | 0 | 0 | 0 | 0 | **0** |
| Using personal computer | 1 | 0 | 1 | 0 | 1 | | | 0 | | 1 | 0 | 0 | | | 0 | 0 | 0 | 0 | 0 | 0 | **0** |
| *Mental health status/history* | | | | | | | | | | | | | | | | | | | | | |
| History of depression | 2 | 1 | 0 | 1 | 0 | | | 0 | | 0 | 0 | 1 | | | 0 | 0 | 1 | 1 | 1 | 0 | 0 |
| History of depression/anxiety | 2 | 1 | 0 | 1 | 1 | | | 0 | | 0 | 1 | 1 | | | 1 | 0 | 0 | 0 | 0 | 0 | 0 |
| History of psychosis | 1 | 0 | 0 | 1 | 0 | | | 0 | | 0 | 0 | 1 | | | 0 | 0 | 1 | 0 | 0 | 0 | 0 |
| History of mental disorder | 1 | 1 | 0 | 0 | 0 | | | 0 | | 0 | 0 | 1 | | | 1 | 0 | 0 | 0 | 0 | 0 | 0 |
| Current alcohol-related or anxiety disorder | 1 | 0 | 0 | 1 | 0 | | | 0 | | 0 | 0 | 0 | | | 0 | 0 | 0 | 1 | 0 | 0 | 1 |
| Depression score at baseline | 2 | 1 | 0 | 1 | 0 | | | 0 | | 0 | 0 | 1 | | | 1 | 0 | 0 | 1 | 0 | 0 | 1 |
| Subsyndromal depression at baseline | 1 | 0 | 0 | 1 | 1 | | | 0 | | 0 | 1 | 0 | | | 0 | 0 | 0 | 0 | 0 | 0 | 0 |
| Delirium in preceding month | 1 | 0 | 0 | 1 | 0 | | | 0 | | 0 | 0 | 1 | | | 0 | 0 | 1 | 0 | 0 | 0 | 0 |
| Family history of mental illness | 2 | 0 | 0 | 2 | 2 | | | 0 | | 0 | 2 | 0 | | | 0 | 0 | 0 | 0 | 0 | 0 | 0 |
| Cognitive function: worse | 7 | 3 | 0 | 4 | 5 | | | 2 | | 0 | 3 | 1 | | | 1 | 0 | 0 | 1 | 0 | 0 | 1 |
| Frontal execute function | 1 | 0 | 0 | 1 | 0 | | | 0 | | 0 | 0 | 1 | | | 0 | 0 | 1 | 0 | 0 | 0 | 0 |
| Subjective memory impairment | 1 | 1 | 0 | 0 | 1 | | | 1 | | 0 | 0 | 0 | | | 0 | 0 | 0 | 0 | 0 | 0 | 0 |
| Dementia | 2 | 0 | 0 | 2 | 0 | | | 0 | | 0 | 0 | 1 | | | 0 | 0 | 1 | 1 | 0 | 0 | 1 |
| *Physical health status/history* | | | | | | | | | | | | | | | | | | | | | |
| Number of illnesses | 3 | 1 | 0 | 2 | 2 | | | 0 | | 0 | 2 | 1 | | | 1 | 0 | 0 | 0 | 0 | 0 | **0** |
| Cumulative illness score | 1 | 0 | 0 | 1 | 0 | | | 0 | | 0 | 0 | 0 | | | 0 | 0 | 0 | 1 | 0 | 0 | **1** |
| Having somatic illness | 2 | 0 | 0 | 2 | 1 | | | 0 | | 0 | 1 | 1 | | | 0 | 0 | 1 | 0 | 0 | 0 | **0** |
| Having severe illness | 1 | 0 | 0 | 1 | 0 | | | 0 | | 0 | 0 | 1 | | | 0 | 0 | 1 | 0 | 0 | 0 | **0** |
| Occurrence of new disease/new medical illness | 2 | 0 | 0 | 2 | 1 | | | 0 | | 0 | 1 | 1 | | | 0 | 0 | 1 | 0 | 0 | 0 | **0** |
| Poorer self-rated health | 6 | 4 | 0 | 2 | 3 | | | 3 | | 0 | 0 | 2 | | | 1 | 0 | 1 | 1 | 0 | 0 | **1** |
| Worsening of self-rated health | 1 | 0 | 0 | 1 | 0 | | | 0 | | 0 | 0 | 1 | | | 0 | 0 | 1 | 0 | 0 | 0 | **0** |
| Chronic diseases | 3 | 3 | 0 | 0 | 2 | | | 2 | | 0 | 0 | 1 | | | 1 | 0 | 0 | 0 | 0 | 0 | **0** |
| New chronic disease | 1 | 1 | 0 | 0 | 0 | | | 0 | | 0 | 0 | 1 | | | 1 | 0 | 0 | 0 | 0 | 0 | **0** |
| Worsening of perceived health stress | 1 | 1 | 0 | 0 | 0 | | | 0 | | 0 | 0 | 1 | | | 1 | 0 | 0 | 0 | 0 | 0 | **0** |
| History of myocardial infarction | 1 | 0 | 0 | 1 | 1 | | | 0 | | 0 | 1 | 0 | | | 0 | 0 | 0 | 0 | 0 | 0 | **0** |
| Myocardial infarction (within last 30 months) | 1 | 0 | 0 | 1 | 0 | | | 0 | | 0 | 0 | 1 | | | 0 | 0 | 1 | 0 | 0 | 0 | **0** |
| Coronary heart disease | 1 | 0 | 0 | 1 | 0 | | | 0 | | 0 | 0 | 1 | | | 0 | 0 | 1 | 0 | 0 | 0 | **0** |
| Pre-existing heart disease | 3 | 2 | 0 | 1 | 1 | | | 1 | | 0 | 0 | 2 | | | 1 | 0 | 1 | 0 | 0 | 0 | **0** |
| Lower HDL cholesterol | 1 | 1 | 0 | 0 | 1 | | | 1 | | 0 | 0 | 0 | | | 0 | 0 | 0 | 0 | 0 | 0 | **0** |
| Diabetes | 2 | 0 | 0 | 2 | 0 | | | 0 | | 0 | 0 | 2 | | | 0 | 0 | 2 | 0 | 0 | 0 | **0** |
| History of Lung disease | 1 | 0 | 0 | 1 | 0 | | | 0 | | 0 | 0 | 1 | | | 0 | 0 | 1 | 0 | 0 | 0 | **0** |
| History of bone disease | 1 | 0 | 0 | 1 | 0 | | | 0 | | 0 | 0 | 1 | | | 0 | 0 | 1 | 0 | 0 | 0 | **0** |
| History of cancer | 1 | 0 | 0 | 1 | 0 | | | 0 | | 0 | 0 | 1 | | | 0 | 0 | 1 | 0 | 0 | 0 | **0** |
| Ongoing cancer | 1 | 0 | 0 | 1 | 0 | | | 0 | | 0 | 0 | 1 | | | 0 | 0 | 1 | 0 | 0 | 0 | **0** |
| Newly diagnosed cancer | 1 | 0 | 0 | 1 | 0 | | | 0 | | 0 | 0 | 1 | | | 0 | 0 | 1 | 0 | 0 | 0 | **0** |
| Ongoing Arthritis or rheumatism | 1 | 1 | 0 | 0 | 0 | | | 0 | | 0 | 0 | 1 | | | 1 | 0 | 0 | 0 | 0 | 0 | **0** |
| History of stroke/stroke | 3 | 2 | 0 | 1 | 1 | | | 1 | | 0 | 0 | 2 | | | 1 | 0 | 1 | 0 | 0 | 0 | **0** |
| New stroke (in last 30 months) | 1 | 0 | 0 | 1 | 0 | | | 0 | | 0 | 0 | 1 | | | 0 | 0 | 1 | 0 | 0 | 0 | **0** |
| Illness of relatives | 1 | 0 | 0 | 1 | 0 | | | 0 | | 0 | 0 | 1 | | | 0 | 0 | 1 | 0 | 0 | 0 | **0** |
| More severe Pain | 1 | 1 | 0 | 0 | 0 | | | 0 | | 0 | 0 | 1 | | | 1 | 0 | 0 | 0 | 0 | 0 | **0** |
| Emergent Pain | 1 | 1 | 0 | 0 | 0 | | | 0 | | 0 | 0 | 1 | | | 1 | 0 | 0 | 0 | 0 | 0 | **0** |
| Higher BMI | 1 | 0 | 0 | 1 | 0 | | | 0 | | 0 | 0 | 1 | | | 0 | 0 | 1 | 0 | 0 | 0 | **0** |
| Hypertension | 2 | 1 | 0 | 1 | 0 | | | 0 | | 0 | 0 | 2 | | | 1 | 0 | 1 | 0 | 0 | 0 | **0** |
| Systolic Blood pressure | 1 | 0 | 0 | 1 | 0 | | | 0 | | 0 | 0 | 1 | | | 0 | 0 | 1 | 0 | 0 | 0 | **0** |
| Diastolic blood pressure | 1 | 0 | 0 | 1 | 0 | | | 0 | | 0 | 0 | 1 | | | 0 | 0 | 1 | 0 | 0 | 0 | **0** |
| Vascular risk factors | 1 | 0 | 0 | 1 | 0 | | | 0 | | 0 | 0 | 1 | | | 0 | 0 | 1 | 0 | 0 | 0 | **0** |
| Number of medications, mean | 1 | 0 | 0 | 1 | 0 | | | 0 | | 0 | 0 | 1 | | | 0 | 0 | 1 | 0 | 0 | 0 | **0** |
| Taking anxiolytic medication | 1 | 0 | 0 | 1 | 0 | | | 0 | | 0 | 0 | 1 | | | 0 | 0 | 1 | 0 | 0 | 0 | **0** |
| Taking Antihypertensive medication | 1 | 0 | 0 | 1 | 0 | | | 0 | | 0 | 0 | 1 | | | 0 | 0 | 1 | 0 | 0 | 0 | **0** |
| Using Sleep medication | 1 | 1 | 0 | 0 | 1 | | | 1 | | 0 | 0 | 0 | | | 0 | 0 | 0 | 0 | 0 | 0 | **0** |
| Poor sleep quality | 1 | 0 | 0 | 1 | 1 | | | 0 | | 0 | 1 | 0 | | | 0 | 0 | 0 | 0 | 0 | 0 | **0** |
| Difficulty initiating sleep | 2 | 2 | 0 | 0 | 1 | | | 1 | | 0 | 0 | 1 | | | 1 | 0 | 0 | 0 | 0 | 0 | **0** |
| Difficulty maintaining sleep | 2 | 1 | 0 | 1 | 1 | | | 1 | | 0 | 0 | 2 | | | 0 | 0 | 1 | 0 | 0 | 0 | **0** |
| Excessive daytime sleepiness | 1 | 0 | 0 | 1 | 0 | | | 0 | | 0 | 0 | 1 | | | 0 | 0 | 1 | 0 | 0 | 0 | **0** |
| Early morning awakening | 2 | 0 | 0 | 2 | 1 | | | 0 | | 0 | 1 | 1 | | | 0 | 0 | 1 | 0 | 0 | 0 | **0** |
| Discomfort feeling in the legs | 1 | 0 | 0 | 1 | 0 | | | 0 | | 0 | 0 | 1 | | | 0 | 0 | 1 | 0 | 0 | 0 | **0** |
| Insomnia symptoms: two or more | 1 | 1 | 0 | 0 | 0 | | | 0 | | 0 | 0 | 1 | | | 1 | 0 | 0 | 0 | 0 | 0 | **0** |
| Insomnia: DIS or DMS 3 night or more per week over last month | 1 | 1 | 0 | 0 | 0 | | | 0 | | 0 | 0 | 1 | | | 1 | 0 | 0 | 0 | 0 | 0 | **0** |
| Subjective sleep sufficiency: insufficient | 1 | 0 | 0 | 1 | 0 | | | 0 | | 0 | 0 | 1 | | | 0 | 0 | 1 | 0 | 0 | 0 | **0** |
| 2 or more specialist visits | 1 | 1 | 0 | 0 | 1 | | | 1 | | 0 | 0 | 0 | | | 0 | 0 | 0 | 0 | 0 | 0 | **0** |
| Hospitalization | 1 | 0 | 0 | 1 | 1 | | | 0 | | 0 | 1 | 0 | | | 0 | 0 | 0 | 0 | 0 | 0 | **0** |
|  | | | | | | | | | | | | | | | | | | | | | |
| *Impairment* | | | | | | | | | | | | | | | | | | | | | |
| Mobility impairment | 3 | 3 | 0 | 0 | 1 | | | 1 | | 0 | 0 | 2 | | | 2 | 0 | 0 | 0 | 0 | 0 | **0** |
| Worsened mobility | 1 | 0 | 0 | 1 | 0 | | | 0 | | 0 | 0 | 1 | | | 0 | 0 | 1 | 0 | 0 | 0 | **0** |
| Physical performance | 1 | 0 | 0 | 1 | 1 | | | 0 | | 0 | 1 | 0 | | | 0 | 0 | 0 | 0 | 0 | 0 | **0** |
| Disabled-sedentary (vs. Mobile active) | 1 | 0 | 0 | 1 | 1 | | | 0 | | 0 | 1 | 0 | | | 0 | 0 | 0 | 0 | 0 | 0 | **0** |
| Disabled-active (vs. Mobile-active) | 1 | 0 | 0 | 1 | 1 | | | 0 | | 0 | 1 | 0 | | | 0 | 0 | 0 | 0 | 0 | 0 | **0** |
| Mobile-sedentary (vs. Mobile-active) | 1 | 0 | 0 | 1 | 1 | | | 0 | | 0 | 1 | 0 | | | 0 | 0 | 0 | 0 | 0 | 0 | **0** |
| Handicap | 1 | 0 | 0 | 1 | 0 | | | 0 | | 0 | 0 | 1 | | | 0 | 0 | 1 | 0 | 0 | 0 | **0** |
| Physical self-maintenance score | 1 | 1 | 0 | 0 | 0 | | | 0 | | 0 | 0 | 0 | | | 0 | 0 | 0 | 1 | 1 | 0 | **0** |
| Karnofsky performance status scale | 1 | 0 | 0 | 1 | 0 | | | 0 | | 0 | 0 | 0 | | | 0 | 0 | 0 | 1 | 0 | 0 | **1** |
| Worsened IADL-impairment | 3 | 3 | 0 | 0 | 1 | | | 1 | | 0 | 0 | 2 | | | 2 | 0 | 0 | 0 | 0 | 0 | **0** |
| Worsened ADL-impairment | 2 | 1 | 0 | 1 | 1 | | | 0 | | 0 | 1 | 1 | | | 1 | 0 | 0 | 0 | 0 | 0 | **0** |
| IADL impairment | 6 | 4 | 0 | 2 | 2 | | | 1 | | 0 | 1 | 3 | | | 3 | 0 | 0 | 1 | 0 | 0 | **1** |
| ADL impairment | 7 | 1 | 0 | 6 | 3 | | | 1 | | 0 | 2 | 4 | | | 0 | 0 | 4 | 0 | 0 | 0 | **0** |
| Visual impairment | 4 | 3 | 0 | 1 | 1 | | | 1 | | 0 | 0 | 3 | | | 2 | 0 | 1 | 0 | 0 | 0 | **0** |
| Near visual impairment | 1 | 0 | 0 | 1 | 1 | | | 0 | | 0 | 1 | 0 | | | 0 | 0 | 0 | 0 | 0 | 0 | **0** |
| Baseline distance visual function loss | 1 | 0 | 0 | 1 | 1 | | | 0 | | 0 | 1 | 0 | | | 0 | 0 | 0 | 0 | 0 | 0 | **0** |
| 2-year decrease in distance visual function | 1 | 1 | 0 | 0 | 1 | | | 1 | | 0 | 0 | 0 | | | 0 | 0 | 0 | 0 | 0 | 0 | **0** |
| Hearing impairment | 4 | 0 | 0 | 4 | 1 | | | 0 | | 0 | 1 | 3 | | | 0 | 0 | 3 | 0 | 0 | 0 | **0** |
| Both visual and hearing impairment | 1 | 0 | 0 | 1 | 0 | | | 0 | | 0 | 0 | 1 | | | 0 | 0 | 1 | 0 | 0 | 0 | **0** |
| Physical frailty | 1 | 1 | 0 | 0 | 1 | | | 1 | | 0 | 0 | 0 | | | 0 | 0 | 0 | 0 | 0 | 0 | **0** |
| Gait speed (slower than 1m/s) | 1 | 1 | 0 | 0 | 1 | | | 1 | | 0 | 0 | 0 | | | 0 | 0 | 0 | 0 | 0 | 0 | **0** |
| Fear of falling | 1 | 0 | 0 | 1 | 1 | | | 0 | | 0 | 1 | 0 | | | 0 | 0 | 0 | 0 | 0 | 0 | **0** |
| *Psychosocial factors* | | | | | | | | | | | | | | | | | | | | | |
| Family support | 1 | 0 | 1 | 0 | 0 | | | 0 | | 0 | 0 | 1 | | | 0 | 1 | 0 | 0 | 0 | 0 | **0** |
| Family negative interaction | 3 | 2 | 0 | 1 | 0 | | | 0 | | 0 | 0 | 2 | | | 2 | 0 | 0 | 1 | 0 | 0 | **1** |
| Emotional support by family | 1 | 0 | 0 | 1 | 0 | | | 0 | | 0 | 0 | 1 | | | 0 | 0 | 1 | 0 | 0 | 0 | **0** |
| Frequency of contact by family | 1 | 0 | 0 | 1 | 0 | | | 0 | | 0 | 0 | 1 | | | 0 | 0 | 1 | 0 | 0 | 0 | **0** |
| Loneliness | 2 | 1 | 0 | 1 | 1 | | | 1 | | 0 | 0 | 1 | | | 0 | 0 | 1 | 0 | 0 | 0 | **0** |
| Receiving Instrumental social support | 1 | 0 | 0 | 1 | 0 | | | 0 | | 0 | 0 | 0 | | | 0 | 0 | 0 | 1 | 0 | 0 | **1** |
| Worsened Instrumental social support | 1 | 0 | 0 | 1 | 0 | | | 0 | | 0 | 0 | 1 | | | 0 | 0 | 1 | 0 | 0 | 0 | **0** |
| Receiving social support | 1 | 0 | 0 | 1 | 1 | | | 0 | | 0 | 1 | 0 | | | 0 | 0 | 0 | 1 | 0 | 0 | **1** |
| Worsened Receiving emotional social support | 1 | 0 | 0 | 1 | 0 | | | 0 | | 0 | 0 | 1 | | | 0 | 0 | 1 | 0 | 0 | 0 | **0** |
| Not having someone to consult when in trouble | 1 | 1 | 0 | 0 | 1 | | | 1 | | 0 | 0 | 0 | | | 0 | 0 | 0 | 0 | 0 | 0 | **0** |
| Not having someone to take care of oneself when ill in bed | 1 | 1 | 0 | 0 | 1 | | | 1 | | 0 | 0 | 0 | | | 0 | 0 | 0 | 0 | 0 | 0 | **0** |
| Not having someone to help with daily housework | 1 | 0 | 0 | 1 | 1 | | | 0 | | 0 | 1 | 0 | | | 0 | 0 | 0 | 0 | 0 | 0 | **0** |
| Not having someone to take you to the hospital | 1 | 0 | 0 | 1 | 1 | | | 0 | | 0 | 1 | 0 | | | 0 | 0 | 0 | 0 | 0 | 0 | **0** |
| Not having someone to consult in bad physical condition | 1 | 0 | 0 | 1 | 1 | | | 0 | | 0 | 1 | 0 | | | 0 | 0 | 0 | 0 | 0 | 0 | **0** |
| being called for advice | 1 | 0 | 0 | 1 | 1 | | | 0 | | 0 | 1 | 0 | | | 0 | 0 | 0 | 0 | 0 | 0 | **0** |
| Participating in events in community center | 1 | 0 | 1 | 0 | 1 | | | 0 | | 1 | 0 | 0 | | | 0 | 0 | 0 | 0 | 0 | 0 | **0** |
| Attending a community meeting | 1 | 0 | 1 | 0 | 1 | | | 0 | | 1 | 0 | 0 | | | 0 | 0 | 0 | 0 | 0 | 0 | **0** |
| No regular visitors | 1 | 0 | 0 | 1 | 0 | | | 0 | | 0 | 0 | 1 | | | 0 | 0 | 1 | 0 | 0 | 0 | **0** |
| Having no friends | 1 | 0 | 0 | 1 | 0 | | | 0 | | 0 | 0 | 1 | | | 0 | 0 | 1 | 0 | 0 | 0 | **0** |
| Being unsatisfied with social network | 1 | 0 | 0 | 1 | 0 | | | 0 | | 0 | 0 | 1 | | | 0 | 0 | 1 | 0 | 0 | 0 | **0** |
| Higher social network score | 1 | 0 | 1 | 0 | 1 | | | 0 | | 1 | 0 | 0 | | | 0 | 0 | 0 | 0 | 0 | 0 | **0** |
| Worsened life satisfaction | 1 | 1 | 0 | 0 | 0 | | | 0 | | 0 | 0 | 1 | | | 1 | 0 | 0 | 0 | 0 | 0 | **0** |
| *Life stressors* | | | | | | | | | | | | | | | | | | | | | |
| Stressful life events | 3 | 0 | 0 | 3 | 1 | | | 0 | | 0 | 1 | 1 | | | 0 | 0 | 1 | 1 | 0 | 0 | 1 |
| Psychological stress | 1 | 1 | 0 | 0 | 0 | | | 0 | | 0 | 0 | 1 | | | 1 | 0 | 0 | 0 | 0 | 0 | 0 |
| Loss of spouse | 2 | 2 | 0 | 0 | 1 | | | 1 | | 0 | 0 | 1 | | | 1 | 0 | 0 | 0 | 0 | 0 | 0 |
| bereavement | 1 | 0 | 0 | 1 | 0 | | | 0 | | 0 | 0 | 1 | | | 0 | 0 | 1 | 0 | 0 | 0 | 0 |
| Troubles with relatives | 1 | 0 | 1 | 0 | 0 | | | 0 | | 0 | 0 | 1 | | | 0 | 0 | 1 | 0 | 0 | 0 | 0 |
| *MRI alterations* | | | | | | | | | | | | | | | | | | | | | |
| Progression of white matter or periventricular hyperintensities in MRI | 1 | 0 | 0 | 1 | 0 | | | 0 | | 0 | 0 | 1 | | | 0 | 0 | 1 | 0 | 0 | 0 | **0** |
| Cella media index in MRI | 1 | 0 | 0 | 1 | 0 | | | 0 | | 0 | 0 | 1 | | | 0 | 0 | 1 | 0 | 0 | 0 | **0** |
| Atrophy of medial temporal lobe in MRI | 1 | 0 | 0 | 1 | 0 | | | 0 | | 0 | 0 | 1 | | | 0 | 0 | 1 | 0 | 0 | 0 | **0** |
| *Risk factors only women* | | | | | | | | | | | | | | | | | | | | | |
| Rural residence | 2 | 2 | 0 | 0 | 2 | | | 2 | | 0 | 0 | 0 | | | 0 | 0 | 0 | - | - | - | **-** |
| Older age | 2 | 1 | 0 | 1 | 1 | | | 0 | | 0 | 1 | 1 | | | 1 | 0 | 0 | - | - | - | **-** |
| Marriage status: widowed/divorced | 1 | 0 | 0 | 1 | 1 | | | 0 | | 0 | 1 | 0 | | | 0 | 0 | 0 | - | - | - | **-** |
| Marriage status: married | 2 | 0 | 0 | 2 | 1 | | | 0 | | 0 | 1 | 1 | | | 0 | 0 | 1 | - | - | - | **-** |
| Education: lower | 1 | 0 | 0 | 1 | 0 | | | 0 | | 0 | 0 | 1 | | | 0 | 0 | 1 | - | - | - | **-** |
| Education: higher | 1 | 0 | 0 | 1 | 0 | | | 0 | | 0 | 0 | 1 | | | 0 | 0 | 1 | - | - | - | **-** |
| Income: lower | 1 | 0 | 0 | 1 | 0 | | | 0 | | 0 | 0 | 1 | | | 0 | 0 | 1 | - | - | - | **-** |
| Economic status | 1 | 0 | 0 | 1 | 1 | | | 0 | | 0 | 1 | 0 | | | 0 | 0 | 0 | - | - | - | **-** |
| Having somatic illness | 1 | 0 | 0 | 1 | 0 | | | 0 | | 0 | 0 | 1 | | | 0 | 0 | 1 | - | - | - | **-** |
| Poorer self-rated health | 1 | 0 | 0 | 1 | 0 | | | 0 | | 0 | 0 | 1 | | | 0 | 0 | 1 | - | - | - | **-** |
| Chronic diseases | 1 | 0 | 0 | 1 | 1 | | | 0 | | 0 | 1 | 0 | | | 0 | 0 | 0 | - | - | - | **-** |
| Chronic pain | 1 | 0 | 0 | 1 | 1 | | | 0 | | 0 | 1 | 0 | | | 0 | 0 | 0 | - | - | - | **-** |
| IADL-impairment | 1 | 0 | 0 | 1 | 1 | | | 0 | | 0 | 1 | 1 | | | 0 | 0 | 1 | - | - | - | **-** |
| No regular contact with family | 2 | 1 | 0 | 1 | 2 | | | 1 | | 0 | 1 | 0 | | | 0 | 0 | 0 | - | - | - | **-** |
| Receiving emotional social support | 1 | 0 | 0 | 1 | 0 | | | 0 | | 0 | 0 | 1 | | | 0 | 0 | 1 | - | - | - | **-** |
| Receiving Instrumental social support | 1 | 0 | 0 | 1 | 0 | | | 0 | | 0 | 0 | 1 | | | 0 | 0 | 1 | - | - | - | **-** |
| Receiving social support | 1 | 0 | 0 | 1 | 0 | | | 0 | | 0 | 0 | 1 | | | 0 | 0 | 1 | - | - | - | **-** |
| Providing emotional social support | 1 | 0 | 0 | 1 | 0 | | | 0 | | 0 | 0 | 1 | | | 0 | 0 | 1 | - | - | - | **-** |
| Providing instrumental social support | 1 | 0 | 0 | 1 | 0 | | | 0 | | 0 | 0 | 1 | | | 0 | 0 | 1 | - | - | - | **-** |
| Higher sense of coherence | 1 | 0 | 0 | 1 | 0 | | | 0 | | 0 | 0 | 1 | | | 0 | 1 | 0 | - | - | - | **-** |
| No regular contact with friends | 2 | 1 | 0 | 1 | 2 | | | 1 | | 0 | 1 | 0 | | | 0 | 0 | 0 | - | - | - | **-** |
| Higher frequency of meeting with friends | 1 | 0 | 0 | 1 | 0 | | | 0 | | 0 | 0 | 1 | | | 0 | 0 | 1 | - | - | - | **-** |
| Having hobbies | 1 | 0 | 0 | 1 | 0 | | | 0 | | 0 | 0 | 1 | | | 0 | 0 | 1 | - | - | - | **-** |
| Number of Household assets | 1 | 0 | 0 | 1 | 1 | | | 0 | | 0 | 1 | 0 | | | 0 | 0 | 0 |  |  |  |  |
| Household floor types | 1 | 0 | 0 | 1 | 1 | | | 0 | | 0 | 1 | 0 | | | 0 | 0 | 0 |  |  |  |  |
| Source of energy for cooking | 1 | 0 | 0 | 1 | 1 | | | 0 | | 0 | 1 | 0 | | | 0 | 0 | 0 |  |  |  |  |
| Source of water supply | 1 | 0 | 0 | 1 | 1 | | | 0 | | 0 | 1 | 0 | | | 0 | 0 | 0 |  |  |  |  |
| Occupational attainment: unskilled | 1 | 0 | 0 | 1 | 1 | | | 0 | | 0 | 1 | 0 | | | 0 | 0 | 0 |  |  |  |  |
| Occupational attainment: elementary | 1 | 0 | 0 | 1 | 1 | | | 0 | | 0 | 1 | 0 | | | 0 | 0 | 0 |  |  |  |  |
| Participating in Organizations | 1 | 0 | 0 | 1 | 0 | | | 0 | | 0 | 0 | 1 | | | 0 | 0 | 1 | - | - | - | **-** |
| Stressful life events | 2 | 1 | 0 | 1 | 1 | | | 0 | | 0 | 1 | 1 | | | 1 | 0 | 0 | - | - | - | **-** |
| Lower HDL-Cholesterol level | 1 | 0 | 0 | 1 | 0 | | | 0 | | 0 | 0 | 1 | | | 0 | 0 | 1 | - | - | - | **-** |
| GPR50 polymorphism | 1 | 1 | 0 | 0 | 0 | | | 0 | | 0 | 0 | 1 | | | 1 | 0 | 0 | - | - | - |  |
| *Risk factors only men* | | | | | | | | | | | | | | | | | | | | | |
| Rural residence | 2 | 0 | 0 | 2 | 2 | | | 0 | | 0 | 2 | 0 | | | 0 | 0 | 0 | - | - | - | **-** |
| Older age | 2 | 0 | 0 | 2 | 1 | | | 0 | | 0 | 1 | 1 | | | 0 | 0 | 1 | - | - | - | **-** |
| Marriage status: widowed/divorced | 1 | 0 | 0 | 1 | 1 | | | 0 | | 0 | 1 | 0 | | | 0 | 0 | 0 | - | - | - | **-** |
| Marriage status: married | 2 | 0 | 0 | 2 | 1 | | | 0 | | 0 | 1 | 1 | | | 0 | 0 | 1 | - | - | - | **-** |
| Education: lower | 2 | 0 | 0 | 2 | 1 | | | 0 | | 0 | 1 | 1 | | | 0 | 0 | 1 | - | - | - | **-** |
| Education: higher | 2 | 0 | 0 | 2 | 1 | | | 0 | | 0 | 1 | 1 | | | 0 | 0 | 1 | - | - | - | **-** |
| Income: lower | 1 | 0 | 0 | 1 | 0 | | | 0 | | 0 | 0 | 1 | | | 0 | 0 | 1 | - | - | - | **-** |
| Economic status | 1 | 0 | 0 | 1 | 1 | | | 0 | | 0 | 1 | 0 | | | 0 | 0 | 0 | - | - | - | **-** |
| Having somatic illness | 0 | 0 | 0 | 0 | 0 | | | 0 | | 0 | 0 | 0 | | | 0 | 0 | 0 | - | - | - | **-** |
| Poorer self-rated health | 1 | 1 | 0 | 0 | 0 | | | 0 | | 0 | 0 | 1 | | | 1 | 0 | 0 | - | - | - | **-** |
| Chronic diseases | 1 | 0 | 0 | 1 | 1 | | | 0 | | 0 | 1 | 0 | | | 0 | 0 | 0 | - | - | - | **-** |
| Chronic pain | 1 | 0 | 0 | 1 | 1 | | | 0 | | 0 | 1 | 0 | | | 0 | 0 | 0 | - | - | - | **-** |
| IADL-impairment | 2 | 0 | 0 | 2 | 1 | | | 0 | | 0 | 1 | 1 | | | 0 | 0 | 1 | - | - | - | **-** |
| No regular contact with family | 2 | 0 | 0 | 2 | 2 | | | 0 | | 0 | 2 | 0 | | | 0 | 0 | 0 | - | - | - | **-** |
| Receiving emotional social support | 1 | 0 | 0 | 1 | 0 | | | 0 | | 0 | 0 | 1 | | | 0 | 0 | 1 | - | - | - | **-** |
| Receiving Instrumental social support | 1 | 0 | 0 | 1 | 0 | | | 0 | | 0 | 0 | 1 | | | 0 | 0 | 1 | - | - | - | **-** |
| Providing emotional social support | 1 | 0 | 0 | 1 | 0 | | | 0 | | 0 | 0 | 1 | | | 0 | 0 | 1 | - | - | - | **-** |
| Providing instrumental social support | 1 | 0 | 0 | 1 | 0 | | | 0 | | 0 | 0 | 1 | | | 0 | 0 | 1 | - | - | - | **-** |
| Participation in family activities | 1 | 0 | 0 | 1 | 1 | | | 0 | | 0 | 1 | 0 | | | 0 | 0 | 0 |  |  |  |  |
| Participation in community activities | 1 | 0 | 0 | 1 | 1 | | | 0 | | 0 | 1 | 0 | | | 0 | 0 | 0 |  |  |  |  |
| Higher sense of coherence | 1 | 0 | 1 | 0 | 0 | | | 0 | | 0 | 0 | 1 | | | 0 | 1 | 0 | - | - | - | **-** |
| No regular contact with friends | 2 | 0 | 0 | 2 | 2 | | | 0 | | 0 | 2 | 0 | | | 0 | 0 | 0 | - | - | - | **-** |
| Having hobbies | 1 | 0 | 1 | 0 | 0 | | | 0 | | 0 | 0 | 1 | | | 0 | 1 | 0 | - | - | - | **-** |
| Number of Household assets | 1 | 0 | 0 | 1 | 1 | | | 0 | | 0 | 1 | 0 | | | 0 | 0 | 0 |  |  |  |  |
| Household floor types | 1 | 0 | 0 | 1 | 1 | | | 0 | | 0 | 1 | 0 | | | 0 | 0 | 0 |  |  |  |  |
| Source of energy for cooking | 1 | 0 | 0 | 1 | 1 | | | 0 | | 0 | 1 | 0 | | | 0 | 0 | 0 |  |  |  |  |
| Source of water supply | 1 | 0 | 0 | 1 | 1 | | | 0 | | 0 | 1 | 0 | | | 0 | 0 | 0 |  |  |  |  |
| Occupational attainment: unskilled | 1 | 1 | 0 | 0 | 1 | | | 1 | | 0 | 0 | 0 | | | 0 | 0 | 0 |  |  |  |  |
| Occupational attainment: trade | 1 | 1 | 0 | 0 | 1 | | | 1 | | 0 | 0 | 0 | | | 0 | 0 | 0 |  |  |  |  |
| Participating in Organizations | 1 | 0 | 0 | 1 | 0 | | | 0 | | 0 | 0 | 1 | | | 0 | 0 | 1 | - | - | - | **-** |
| Stressful life events | 2 | 1 | 0 | 1 | 1 | | | 0 | | 0 | 1 | 1 | | | 1 | 0 | 0 | - | - | - | **-** |
| Lower LDL-Cholesterol level | 1 | 1 | 0 | 0 | 0 | | | 0 | | 0 | 0 | 1 | | | 1 | 0 | 0 | - | - | - | **-** |
| GPR50 polymorphism | 0 | 0 | 0 | 0 | 0 | | | 0 | | 0 | 0 | 0 | | | 0 | 0 | 0 | - | - | - |  |

*n = number of studies; + = significant predictive factor for incident depression ; - = significant protective factor against incident depression*
