# Supplementary material for: Risk factors and protective factors of depression in older people 65+. A systematic review
Source: PLoS One. 2021 May 13;16(5):e0251326. doi: 10.1371/journal.pone.0251326 (PMC8118343; doi:10.1371/journal.pone.0251326)
Supplement: S1 File — (DOCX) [file pone.0251326.s003.docx]

# Risk of Bias assessment

# Using QUIPS (Quality in Prognostic Studies)

# (Hayden et al. 2013)

**Chou et al. 2007(Chou 2007b)** Chou, Kee-Lee (2007b): Reciprocal relationship between pain and depression in older adults: evidence from the English Longitudinal Study of Ageing. In: *Journal of affective disorders* 102 (1-3), S. 115–123. DOI: 10.1016/j.jad.2006.12.013.

| **Risk of bias item** | **Author`s judgement** | **Support for judgement** |
| --- | --- | --- |
| Study participation | **Low risk of bias**. The relationship between the PF (Prognostic Factors) and outcome is unlikely to be different for participants and eligible nonparticipants. | 64.3% of eligible persons participated. Source population described random sample of 50+. Baseline sample described. Sampling frame and recruitment adequately described. Period and place of recruitment adequately described. Inclusion and Exclusion criteria adequately described. |
| Study Attrition | **Moderate risk of bias.** The relationship between PF may be different for completing and non-completing participants. | Response rate adequate for study participants (78%). Adequate description of dropped out participants reported. Reasons for follow-up reported (13,6% could not be contacted, 76,5% refused, 9,5% ill or away during survey period). Non-respondents were more likely to have limiting chronic illness, live at London, to be of non-white ethnicity, single or married..) |
| Prognostic Factor Measurement | **High risk of bias** the measurement of the PF is very likely to be different for different levels of the  outcome of interest. | Clear definition and description of prognostic factors was provided. Method and measurement of PF was the same for all participants**. 652 of 9432 were interviewed by proxy.** |
| Outcome measurement | **High risk of bias.** The measurement outcome may be different related to baseline level of RF or may be influenced by interview in person and by proxy interview. | Method was the same for all participants. Method of outcome measurement adequately valid. However, **652 of 9432 were interviewed by proxy.** |
| Study Confounding | **Low risk of bias.** The observed effect on the PF on outcome is unlikely to be distorted by another factor related to PF and outcome. | All important confounders were measured. Clear definitions of important confounders provided and included in model. Measurement of all important confounders is adequately valid and reliable. Method and setting for confounding measurement the same for all study participants |
| Statistical analysis and reporting | **Low risk of bias.** The reported results are unlikely.  to be spurious or biased  related to analysis or reporting | Statistical model was adequate. Significant and insignificant results were reported. All factors included in the model were reported. |

Chou et al. 2007 (Chou 2007a) Chou, Kee-Lee (2007a): Combined effect of vision and hearing impairment on depression in older adults: Evidence from the English Longitudinal Study of the Ageing. In: *Journal of affective disorders*, S. 191–196.

| **Risk of bias item** | **Author`s judgement** | **Support for judgement** |
| --- | --- | --- |
| Study participation | **Low risk of bias**. The relationship between the PF (Prognostic Factors) and outcome is unlikely to be different for participants and eligible nonparticipants. | 64.3% of eligible persons participated. Source population described random sample of 50+. Baseline sample described. Sampling frame and recruitment adequately described. Period and place of recruitment adequately described. Inclusion and Exclusion criteria adequately described. |
| Study Attrition | **Moderate risk of bias.** The relationship between PF may be different for completing and non-completing participants. | Response rate adequate for study participants (78%). Adequate description of dropped out participants reported. Reasons for follow-up reported (13,6% could not be contacted, 76,5% refused, 9,5% ill or away during survey period). However, non-respondents were more likely to have limiting chronic illness, live at London, to be of non-white ethnicity, single or married..) |
| Prognostic Factor Measurement | **Low risk of bias:** The measurement of the PF is unlikely to be different for different levels of the outcome of interest | Not for all PFs a clear definition was provided (marital status, education, employment status), but for most factors. However, no information made the reviewers suspect application of different measurement for different participants. Method for measurement of PF was same for all participants. In this study, only data from individuals interviewed directly were analyzed. |
| Outcome measurement | **Low risk of bias.** The measurement of the outcome is unlikely to be different related to  the baseline level of the PF. | Clear definition of outcome provided. Method of outcome measurement valid and reliable. Method and setting of outcomes same for all study participants. |
| Study Confounding | **Low risk of bias.** The observed effect of the PF  on outcome is unlikely to be distorted by another factor related to PF and outcome | Many important confounders were measured. Clear definitions of important confounders provided and included in model. Measurement of all importan ocnfounders is adequately valid and reliable. Method and setting for confounding measurement the same for all study participants |
| Statistical analysis and reporting | **Low risk of bias.** The reported results are unlikely  to be spurious or biased related to analysis or reporting. | Sufficient presentation of data to assess adequacy of analytic strategy. Strategy appropriate. No selective reporting of results (results of different models presented). |

Conde-Sala et. Al 2019 (Conde-Sala et al. 2019)

| **Risk of bias item** | **Author`s judgement** | **Support for judgement** |
| --- | --- | --- |
| Study participation | **Low risk of bias.** The relationship between the PF and outcome is unlikely tob e different for participants and eligible nonparticipants. | Adequate response rate (69.4/67.1%) in longitudinal sample. Description of target population is given. Description of baseline sample given. Description of period and place of recruitment is given. Descriptionof inclusion and exclusion criteria adequate. |
| Study Attrition | **High risk of bias**. The relationship between PF and outcome is very likely tob e different for completing and noncompleting participants. | Adequate response rate (69.4/67.1%) in longitudinal sample. Information about persons who dropped out provided: older, fewer years of schooling, more commoly female,, porer self-rated health, more depressive symptoms, greater impairment. Reasons for loss to follow-up not provided. The participants and those dropping out differed in the above mentioned variables. |
| Prognostic Factor Measurement | **Low risk of bias**. The measurement of  the PF is unlikely to be different for different levels of the outcome of interest. | Clear defiiton of PFs provided. Method of PF measurement was valid and relaible. Instruments were the same for all participants. Adequate proportion of sample completed dara for PF (67.1%). |
| Outcome measurement | **Low risk of bias.** The measurement of the  outcome is unlikely to be different related to  the baseline level of the PF. | Clear definition of outcome. Method of outcome valid and realiable. Method and setting same for all participants. |
| Study Confounding | **Moderate risk of bias.** The observed effect of the PF  on outcome may be distorted by another factor related to PF and outcome. | Many common and importantant confounders were measured, valid and realiable and accounted for in the analysis.. However e.g. Alcohol consumption. Hearing and visual impariment were not measured. |
| Statistical analysis and reporting | **Low risk of bias**. The reported results are unlikely  to be spurious or biased  related to analysis or reporting | Sufficient pressentation of data to assess adequacy of analytic strategy. Strategy for moedel appropriate. Selected statistical model adequate. No selective reporting (results of multiple models provided. |

Dong et al. 2019 (Dong und Yang 2019) Dong, Yutong; Yang, Frances Margaret (2019): Insomnia symptoms predict both future hypertension and depression. In: *Preventive Medicine* 123, S. 41–47. DOI: 10.1016/j.ypmed.2019.02.001.

| **Risk of bias item** | **Author`s judgement** | **Support for judgement** |
| --- | --- | --- |
| Study participation | **High risk of bias.** The relationship between the  PF and outcome is very likely to be different for  participants and eligible nonparticipants. | Participation rate not avialable to the author. Description of the source  population or population of interest provided. Description of the baseline study sample provided. Adequate description of the sampling frame and recruitment Adequate description of the period and place of  recruitment provided. Participants with hypertension excluded. Therefore probably not representative. |
| Study Attrition | **High risk of bias.** The relationship between the PF and outcome is very likely to be different for completing and noncompleting participants. | No information on participants lost to follow- up or reasons for loss- to folllow- up described. Therefore differences can´t be evaluated. |
| Risk Factor Measurement | **Medium risk of bias**: The measurement of  the PF may be different for different levels of the outcome of interest. | Prognostic factors measured in a similar way for all participants. Clear definition of PFs provided. Continous variables reported appropriately. Measurement of PF same for all participants. No information of proportion of study sample with complete data for PF in the specific age group. |
| Outcome measurement | **Moderate risk of bias:** The measurement of the  outcome may be different related to the  baseline level of the PF | Clear difinition of outcome provided. MEtho adequately valid and reliable (CES-D-8). Method and setting of outcome measurement not the same for all study participants: „e study has been conducted via both telephone and in-person interviews (approximately 50% each method)” |
| Study Confounding | **High risk of bias.** The observed effect of the PF  on the outcome is very likely to be distorted by another factor related to PF and outcome. | Important confounders such as ADL, IADL impairment, other forms of impairment or comorbidities were not assessed or included into the analyiss. |
| Statistical analysis and reporting | **Low risk of bias.** The reported results are unlikely  to be spurious or biased related to analysis or reporting. | Presentation of data sufficient to assess adequacy of the analytic strateg. Strategy for model building appropriate. Selected model adequate for designof the study. No selective reporting could be detected. |

Kim et al. 2006 (Kim et al. 2006) Kim, Jae-Min; Stewart, Robert; Kim, Sung-Wan; Yang, Su-Jin; Shin, Il-Seon; Yoon, Jin-Sang (2006): Vascular risk factors and incident late-life depression in a Korean population. In: *The British journal of psychiatry : the journal of mental science* 189, S. 26–30. DOI: 10.1192/bjp.bp.105.015032.

| **Risk of bias item** | **Author`s judgement** | **Support for judgement** |
| --- | --- | --- |
| Study participation | **High risk of bias.** The relationship between the  PF and outcome is very likely to be different for  participants and eligible nonparticipants. | Population of interest all people > 65 in an area described.Description of baseline sample provided. No informaton about rate of participation provided. Description of period and place of recruitent provided. Inclusion and exlusion criteria provided in other studies on the same sample. |
| Study Attrition | **Low risk of bias**. The relationship between the PF and outcome is unlikely to be different for completing and noncompleting participants. | Adequate response rate for participants in both follow ups (521/631). Adequate description of participants lost to follow up. Information about reasons for loss to follow-up are provided. „ There were no substantial differences in demographic characterisitcs, disability and cognitive function between the two groups.“ |
| Prognostic Factor Measurement | **Moderate risk of bias.** The measurement of  the PF may be different for different levels of the outcome of interest. | Clear definition of most Prognostic factors. Method for most Prognostic factors also valid and reliable. However, treatment histories of stroke, heart disease, hypertension and diabetes were self-reported. Continuous variables were reported. Methods were the same for all participants. Adequate proportion of sample had complete data (521/631). |
| Outcome measurement | **Low risk of bias**. The measurement of the outcome is unlikely to be different related to the baseline level of the PF. | GMS-Agecat with cut-off level of 3 was used for all participants at baseline and follow-up, which is valid and reliable. |
| Study Confounding | **Moderate risk of bias**. The observed effect of the PF on outcome may be distorted by another factor related to PF and outcome. | Several potential confounders, like alcohol consumption, smoking, marital status were not assessed or accounted for in the analysis. Measurement of the assessed confounders was adequate, reliable and the same for all participants. |
| Statistical analysis and reporting | **Low risk of bias.** The reported results are unlikely to be spurious or biased related to analysis or reporting | Sufficient data was available to assess the adequacy of analytic strategy and the model was adequate. There were no hints to selective reporting in the results. |

Forsell 2000 (Forsell 2000) Forsell, Y. (2000): Y. Forsell Predictors for Depression, Anxiety and psychotic Predictors for Depression, Anxiety and psychotic symptoms in a very elderly population: data from a 3-year follow-up study. In: *Social psychiatry and psychiatric epidemiology* 2000 (35), S. 259–263. Online verfügbar unter https://link.springer.com/content/pdf/10.1007%2Fs001270050237.pdf, zuletzt geprüft am 08.10.2019.

| **Risk of bias item** | **Author`s judgement** | **Support for judgement** |
| --- | --- | --- |
| Study participation | **High risk of bias.** The relationship between the  RF and outcome is very likely to be different for participants and eligible nonparticipants. | Description of source population (all persons 75 and over in a district). Participation was adequate 2368 participated and 377 refused to participate. Description of baseline sample provided. Sampling frame and recruitment described. Period an place of recruitment described adequately. Inclusion and exclusion criteria provided (Depression, psychotic or anxiety symptoms and baseline) Participants with anxiety or psychotic symptoms at baseline were excluded. |
| Study Attrition | **High risk of bias.** The relationship between the PF and outcome is very likely to be different for completing and non-completing participants. | Response rate for study participants adequate (377 of 2368 refused to participate at baseline, 1101 of 1279 surviving participants participated in follow-up. Reasons for loss to follow-up were provided. There was no adequate description of participants lost to follow-up. |
| Prognostic Factor Measurement | **Low risk of bias**. The measurement of  the PF is unlikely to be different for  different levels of the outcome of interest | Clear definition of prognostic factors weas provided. Appropriate cut-offs were used. Methods were the same for all participants. Adequate proportion of sample has complete data for Ps (1101/2368 originally selected). Measurement of social network variables were vage „having no regular visitors“, „Having no friends“, „Being unsatisfied with social network. However, this most likely does not influence this type of bias. |
| Outcome measurement | **High risk of bias**. The measurement of the  outcome is very likely to be different related  to the baseline level of the PF | Definition of depression „syndromes“ according to DSM-IV not further specified. |
| Study Confounding | **Moderate risk of bias**. The observed effect of the PF on outcome may be distorted by another factor related to PF and outcome. | Most important confounders were measured and clearly defined. However, some potential confounders such as smoking and alcohol consumption were not assessed or accounted for in the analysis. |
| **Statistical analysis and reporting** | **Low risk of bias**. The reported results are unlikely  to be spurious or biased related to analysis or reporting. | Sufficient presentation of data to assess the adequacy oft he analytic strategy. Model building appropriate. Selected statistical model adequate. No selective reporting of results could be detected. |

Gureje et al. 2011 (Gureje et al. 2011) Gureje, O.; Oladeji, B.; Abiona, T. (2011): Incidence and risk factors for late-life depression in the Ibadan Study of Ageing. In: *Psychological medicine* 41 (9), S. 1897–1906. DOI: 10.1017/S0033291710002643.

| **Risk of bias item** | **Author`s judgement** | **Support for judgement** |
| --- | --- | --- |
| Study participation | **Low risk of bias** The relationship between the  PF and outcome is unlikely to be different for  participants and eligible nonparticipants. | Source population ain 8 regions and mulit-stage random sampling of households described. Sampling frame and description of period and place adequate. Inclusion and exclusion criteria described. Description of baseline sample provided (for men and women separately. |
| Study Attrition | **Moderate risk of bias.** The relationship between the PF and outcome may be different for completing and non-completing participants | Response rate from baseline to follow-u 65.5%, adequate. Reasons for loss to follow-up provided. Participants not lost-to follow-up were least likely to belong to lowest economic class. No difference in sex, residence, educational level or presence of absence of subsyndromal depression. |
| Prognostic Factor Measurement | **Low risk of bias**. The measurement of  the PF is unlikely to be different for  different levels of the outcome of interest. | Clear definition of PFs provided. Methods of measurements valid and reliable. Methods were the same for all participants. Adequate proportion of study sample has complete data (65,5% of baseline sample). Cut points used appropriate. |
| Outcome measurement | **Low risk of bias.** The measurement of the  outcome is unlikely to be different related to  the baseline level of the PF. | Diagnosis MDD according to DSM-IV criteria clearly defined as outcome. Therefore, method of measurements valid and reliable. Method was the same for all study participants. |
| Study Confounding | **High risk of bias.** The observed effect of the PF  on the outcome is very likely to be distorted by another factor related to PF and outcome. | Not all relevant confounders were assessed and accounted for in study design and analysis. E.g. Educational level was not accounted for in the analysis. |
| Statistical analysis and reporting | **Low risk of bias.** The reported results are unlikely  to be spurious or biased related to analysis or reporting. | Sufficient presentation of data to assess adequacy of the analytic strategy. Statistic model adequate for design. No clear evidence for selective reporting of results. |

Jaussent et al. 2011 (Jaussent et al. 2011) Jaussent, Isabelle; Bouyer, Jean; Ancelin, Marie-Laure; Akbaraly, Tasnime; Pérès, Karine; Ritchie, Karen et al. (2011): Insomnia and daytime sleepiness are risk factors for depressive symptoms in the elderly. In: Sleep 34 (8), S. 1103–1110. DOI: 10.5665/SLEEP.1170.

| **Risk of bias item** | **Author`s judgement** | **Support for judgement** |
| --- | --- | --- |
| Study participation | **Moderate risk of bias.** The relationship between the PF and outcome may be different for participants and eligible nonparticipants. | Description of source population adequate. Sampling frame and recruitment described in detail. Time and period of recruitment described in detale. Adequate inclusion and exclusion criteria. Rate of participation of contacted persons quite low (37%). „Those taking antidepressant medication at baselien or at follow up“ were excluded. Therefore, participants potentially developing incident depression were excluded. |
| Study Attrition | **Moderate risk of bias**. The relationship between the PF and outcomee may be different for completing and noncompleting participants. | Response rate for re-examination adequate (90%). Reasons for lost to follow up only partially provided „712 lost to follow-up“ without specification. Participats „not included in the analysis“ described. Participants „not included in the analysis“ had lower educational level, were older and more frequentl female, living alone, with chronic disease and disability. |
| Prognostic Factor Measurement | **Low risk of bias.** The measurement of  the PF is unlikely to be different for different levels of the outcome of interest. | Clear definitionsof prognostic factors were provided. Method of measurement was same for all participants, valid and reliable. Appropriate cut points were used. Adquate proportion of sample has complete data (3824 subjects of 7.810 without depression or dementia at bl). |
| Outcome measurement | **Low risk of bias.** The measurement of the outcome is unlikely to be different related to the baseline level of the PFs. | Outcome variable CES-D with clear cut-off point. Therefore method of outcome valid. Method and setting same for all participants. |
| Study Confounding | **Low risk of bias.** The observed effect of the PF  on outcome is unlikely to be distorted by another factor related to PF and outcome. | All important confounders were measured (including past major depression). Measurements valid and relaiable. Method and setting the same for all study participant. Important potential confounders were accounted for in the analysis. |
| Statistical analysis and reporting | **Low risk of bias.** The reported results are unlikely  to be spurious or biased related to analysis or reporting. | Sufficient presentation of data to assess adequacy of analytic strategy. Selected statistical model adequate for design oft the study. No hints to selective reporting of results. |

Kim et al. 2009 (Kim et al. 2009) Kim, Jae-Min; Stewart, Robert; Kim, Sung-Wan; Yang, Su-Jin; Shin, Il-Seon; Yoon, Jin-Sang (2009): Insomnia, depression, and physical disorders in late life: a 2-year longitudinal community study in Koreans. In: *Sleep* 32 (9), S. 1221–1228. DOI: 10.1093/sleep/32.9.1221.

| **Risk of bias item** | **Author`s judgement** | **Support for judgement** |
| --- | --- | --- |
| Study participation | **Low risk of bias.** The relationship between the  PF and outcome is unlikely to be different for  participants and eligible nonparticipants. | All participants >= 65 of age within 2 geographic catchment areas were approached. Source population described adequately. Baseline sample was not described. Sampling frame and recruitment were described in detail. Period and place of recruitment described adequately. Inclusion and exclusion criteria described (Inclusio: All inhabitants in registration list >= 65 years, for statistical analysis only participants without baseline depression were selected) |
| Study Attrition | **High risk of bias.** The relationship between the PF and outcome is very likely to be different for completing and noncompleting participants. | 909 of 1204 participants completing baseline evaluation completed the follow-up evaluation. Reasons for loss to follow-up given in Figure. „Those present at follow-up were not significantly different at baseline with respect to age (....), gender (…), or prevalence of insomnia (…) at baseline.“ However, differences i those completing and those lost to follow-up were not reported. Differences can therefore be suspected, but not evaluated. |
| Prognostic Factor Measurement | **Low risk of bias**. The measurement of  the PF is unlikely to be different for different levels of the outcome of interest. | Prognostic factor measurment was clearly defined, adequately valid and relaiable, and was the same for all study participants. As mentioned before 909 of 1204 had complete data fort he PFs. For all variables appropriate cut-offs were used. |
| Outcome measurement | **Low risk of bias.** The measurement of the  outcome is unlikely to be different related to  the baseline level of the PF. | Clear definition of outcome (GMS-AGECAT level => 3 used to define depression). This method is valid and reliable. The method was used for al study participants. |
| Study Confounding | **High risk of bias.** The observed effect of the PF  on the outcome is very likely to be distorted by another factor related to PF and outcome. | Important potential confounders were missing (smoking, measurements for impairment). Other important confounders measured valid and reliable and accounted for in study design and analysis. |
| Statistical analysis and reporting | **Low risk of bias**. The reported results are unlikely  to be spurious or biased related to analysis or reporting. | Presentation of data was sufficient to assess adequacy of analytic strategy. Strategy for model building was appropriate. Selected statistical model was adequate. We found no hints to selective reporting. |

Koizumi et al. 2005 (Koizumi et al. 2005) Koizumi, Yayoi; Awata, Shuichi; Kuriyama, Shinichi; Ohmori, Kaori; Hozawa, Atsushi; Seki, Toru et al. (2005): Association between social support and depression status in the elderly: results of a 1-year community-based prospective cohort study in Japan. In: *Psychiatry and clinical neurosciences* 59 (5), S. 563–569. DOI: 10.1111/j.1440-1819.2005.01415.x.

| **Risk of bias item** | **Author`s judgement** | **Support for judgement** |
| --- | --- | --- |
| Study participation | **Moderate risk of bias**. The relationship between the PF and outcome may be different for participants and eligible nonparticipants. | Source of population all individuals >= 70 in an area. 1169 of 2730 answered GDS completely. No complete description of baseline sample, but baseline characteristics of participants completing follow-up and dropouts. Period and recruitment process described adequately. Inclusion criteria (>= 70 years) and exclusion criteria (cognitive dysfunction, MMSE< 18) described. |
| Study Attrition | **Moderate risk of bias.** The relationship between the PF and outcome may be different for  completing and non-completing participants | Response rate adequate. 475 of 753 nondepressed participants at baseline complete follow-up. Reasons for loss to follow-up given (non-participants, answered GDS incompletely). Baseline characteristics of participants completing follow-up and those dropped out given in detail. However, „ women, over 75 years of age, with a low education level, having cognitive dysfunction (MMSE<25), and with physical dysfunction were more likely to drop out. |
| Prognostic Factor Measurement | **High risk of bias.** The measurement of  the PF is very likely to be different for different levels of the outcome of interest. | Prognostic factors clearly defined. „validity and reliability of this questionnaire were not evaluated“ (Referring to a social support questionnaire). Appropriate cut points were used. Adequate proportion onf participants had complete data for the PF. As validity and reliability of the questionnaire were not evaluated, we judged the risk of bias to be high. |
| Outcome measurement | **Low risk of bias.** The measurement of the  outcome is unlikely to be different related to the baseline level of the PF. | Clear definition of outcome provided (GDS30 >11). Method valid and reliable. Method and setting same for all study participants. |
| Study Confounding | **Moderate risk of bias.** The observed effect of the PF  on outcome may be distorted by another factor related to PF and outcome. | Many important confounders were measured with clear definitions, were the same for all study participants and were accounted for in the analysis. However, important potential confounders were not assess (alkohol consumption, smoking) and no validated tool, like ADL or IADL were used to assess impairment. |
| Statistical analysis and reporting | **Low risk of bias.** The reported results are unlikely  to be spurious or biased related to analysis or reporting. | Logistic regression model adequate for design of study. No hints for selective reporting. Sufficient presentation of data to assess the adequacy of analytic strategy. |

Lampinen et al. 2003 (Lampinen und Heikkinen 2003) Lampinen, Päivi; Heikkinen, Eino (2003): Reduced mobility and physical activity as predictors of depressive symptoms among community-dwelling older adults: an eight-year follow-up study. In: *Aging clinical and experimental research* 15 (3), S. 205–211.

| **Risk of bias item** | **Author`s judgement** | **Support for judgement** |
| --- | --- | --- |
| Study participation | **Moderate risk of bias.** The relationship between the PF and outcome may be  different for participants and eligible nonparticipants. | „80% of eligible men and 78% of the women took part in the baseline interview.“ Source population to randomly selected samples of residents of Jyväskylä aged 65 years and over form local population register. Baseline characteristics only provided separate for participants in follow-up, diseased and other dropouts. Period and place of recruitment adequately described. Inclusion and exclusion criteria provided. |
| Study Attrition | **Moderate risk of bias.** The relationship between the PF and outcome may be different for  completing and non-completing participants. | 91% of the men and 87%of women took part in follow-up interview. Reasons to drop out only partly given. Participants lost to follow up adequately described. Drop-outs and deceased were more often disabled and were more often sedentary. |
| Prognostic Factor Measurement | **High risk of bias**. The measurement of  the PF may be different for different levels of the outcome of interest. | Clear definition of PFs provided. No information about validity and reliability of measurement of mobility and impairment was provided. Continuous variables were reported. Method and setting for measurement were same for all participants. Adequate proportion had complete data for PFs. |
| Outcome measurement | **Moderate risk of bias.** The measurement of the  outcome is unlikely to be different related to the baseline level of the PF. | Outcome was measured with Beck´s depression scale. Which „has satisfactory internal consistency and stability for use with older adults in research“. Method was the same for all study participants. |
| Study Confounding | **High risk of bias**. The observed effect of the PF  on the outcome is very likely to be distorted by another factor related to PF and outcome. | Many important confounders were not considered in statistical analysis. E.g. marital status, alcohol consumption were not assessed. Additionally, no validated tool for measuring impairment was used. |
| Statistical analysis and reporting | **Low risk of bias**. The reported results are unlikely  to be spurious or biased related to analysis or reporting. | Selected statistical model (Logistic regression analysis) was adequate for design of the study. We found not hints to selective reporting of results. |

Lue et al. 2010 (Lue et al. 2010) Lue, Bee-Horng; Liang-Ju, Chen; Shwu-Chong, Wu (2010): Health, financial stresses, and life satisfaction affecting late-life depression among older adults: a nationwide, longitudinal survey in Taiwan. In: *Archives of Gerontology and Geriatrics* 2010 (50), S. 34–38.

| **Risk of bias item** | **Author`s judgement** | **Support for judgement** |
| --- | --- | --- |
| Study participation | **Moderate risk of bias**. The relationship between the PF and outcome may be different for participants and eligible nonparticipants. | No information about participation rate of eligible persons provided. Source population were certain townships chosen by a probability sampling technique as primary sampling unit. Baseline characteristics of the sample provided. Inclusion criteria was provided. |
| Study Attrition | **High risk of bias.** The relationship between the PF and outcome is very likely to be different for completing and noncompleting participants. | Of 2890 respondents at baseline 2154 completed the follow-up survey. Reasons for loss to follow-up were mostly provided (death 87,9%, incomplete interview 8.2%, lost to follow-up 3.9%). No adequate description of participants lost to follow-up was provided. Therefore, information on differences between participants who completed the study and those who did not was not provided. |
| Prognostic Factor Measurement | **Moderate risk of bias.** The measurement of  the PF may be different for different levels of the outcome of interest. | Clear definition of all PFs was provided. No information on validity or reliability for assessment of different Social support variables was provided. Methods of measurement were the same for all participants. As described above, adequate proportion of study sample had complete data for PF. |
| Outcome measurement | **Low risk of bias.** The measurement of the outcome is unlikely to be different related to  the baseline level of the PF. | Depression was defined as CES-D-10 >= 10. This method is adequately valid and reliable. Method and setting of outcome measurement were the same for all study participants. |
| Study Confounding | **High risk of bias.** The observed effect of the PF  on the outcome is very likely to be distorted by another factor related to PF and outcome. | Important potential confounders such as Impairment, smoking or alcohol consumption were not accounted in the analysis. |
| Statistical analysis and reporting | **Low risk of bias**. The reported results are unlikely  to be spurious or biased related to analysis or reporting | Sufficient presentation of data to assess the adequacy of the analytic strategy. Selected model adequate. No hints to selective reporting of results. |

Luppa et al. 2012 (Luppa et al. 2012) Luppa, Melanie; Luck, Tobias; König, Hans-Helmut; Angermeyer, Matthias C.; Riedel-Heller, Steffi G. (2012): Natural course of depressive symptoms in late life. An 8-year population-based prospective study. In: *Journal of affective disorders* 142 (1-3), S. 166–171. DOI: 10.1016/j.jad.2012.05.009.

| **Risk of bias item** | **Author`s judgement** | **Support for judgement** |
| --- | --- | --- |
| Study participation | **Low risk of bias.** The relationship between the  PF and outcome are unlikely to be different for  participants and eligible nonparticipants. | Source population described which represents the population of interest adequately. 1,265 of 1,692 eligible persons participated in the baseline assessment. Baseline characteristics of participants described adequately. Period and place of baseline sample described in previous studies about the sample. |
| Study Attrition | **Moderate risk of bias.** The relationship between the PF and outcome may be different for  completing and non-completing participants. | Response rate in follow-ups provided and adequate. Reasons for loss-to follow-up provided in detail. No description of participants lost to follow-up was provided. Therefore, important differences between participants who completed the study and those who did not could not be judged. |
| Prognostic Factor Measurement | **Low risk of bias**. The measurement of  the PF is unlikely to be different for  different levels of the outcome of interest. | Method for assessment of all prognostic factors was clearly defined, valid and reliable, continuous variables were reported. Adequate proportion of study sample hat complete data for the PF. Method for assessment of PFs were the same for all participants. |
| Outcome measurement | **Low risk of bias.** The measurement of the  outcome is unlikely to be different related to  the baseline level of the PFs. | Outcome of interest clearly defined (CES-D-20 score >= 23). Method is valid and reliable. Method was the same for all study participants. |
| Study Confounding | **Low risk of bias.** The observed effect of the PF  on outcome is unlikely to be distorted by another factor related to PF and outcome. | All potentially important confounders except smoking were measured with valid and reliable instruments, clearly defined and the same for all participants. All those confounders were accounted for in the statistical analysis. |
| Statistical analysis and reporting | **Low risk of bias.** The reported results are unlikely.  to be spurious or biased related to analysis or reporting. | Sufficient presentation of data to assess adequacy of analytic strategy. Selected statistical model adequate. No hints for selective reporting. |

Lyness et al. 2009 (Lyness et al. 2009) Lyness, Jeffrey M.; Yu, Qin; Tang, Wan; Tu, Xin; Conwell, Yeates (2009): Risks for depression onset in primary care elderly patients: potential targets for preventive interventions. In: *The American journal of psychiatry* 166 (12), S. 1375–1383. DOI: 10.1176/appi.ajp.2009.08101489.

| **Risk of bias item** | **Author`s judgement** | **Support for judgement** |
| --- | --- | --- |
| Study participation | **High risk of bias.** The relationship between the  PF and outcome is very likely to be different for  participants and eligible nonparticipants. | Study population: All subjects >= 65 years presenting for care in private practices and University-affiliated clinics in interal medicine, geriatrics, and family medicine in Monroe County, N.Y. on selected recruitment days. Participation rate 50.1% of these subjects. No description of baseline study sample provided. Due to lack of information on the days on which recruitment took place, as well as missing information on baseline sample, we judge the risk of bias to be high. |
| Study Attrition | **Moderate risk of bias.** The relationship between the PF and outcome may be different for  completing and noncompleting participants. | 405 of 617 completed on year follow up, 54 of 617 at bl completed 4-year follow-up. Information on participants who dropped out partially given. No reasons for loss to follow-up provided. „Subjects who did complete at least one annual follow-up assessment did not differ from those subjects who did not complete at least one annual follow-up assessment with regard to demographic variables and HAM-D and Cumulative Illness Rating scores but were more likely to be Caucasian. “ No information on differences in other assessed factors between these two groups. |
| Prognostic Factor Measurement | **Low risk of bias**. The measurement of  the PF is unlikely to be different for  different levels of the outcome of interest. | Clear definition of PF is provided. Method is adequately valid and reliable. Appropriate cut-off points were used. Method was the same for all study participants. |
| Outcome measurement | **Low risk of bias.** The measurement of the  outcome is unlikely to be different related to  the baseline level of the PF. | DSM-IV and HAM-D were used to examine depressive symptoms/severity of depressive symptoms. These are valid and reliable methods. Method and setting of outcome were the same for all study participants. |
| Study Confounding | **High risk of bias.** The observed effect of the PF  on the outcome is very likely to be distorted by another factor related to PF and outcome. | No clear understandable information about which confounders were accounted for in the analysis. |
| Statistical analysis and reporting | **No decision** | Statistical model was not fully comprehensible tot he author. |

Makizako et al. 2015 (Makizako et al. 2015) Makizako, Hyuma; Shimada, Hiroyuki; Doi, Takehiko; Yoshida, Daisuke; Anan, Yuya; Tsutsumimoto, Kota et al. (2015): Physical frailty predicts incident depressive symptoms in elderly people: prospective findings from the Obu Study of Health Promotion for the Elderly. In: *Journal of the American Medical Directors Association* 16 (3), S. 194–199. DOI: 10.1016/j.jamda.2014.08.017.

| **Risk of bias item** | **Author`s judgement** | **Support for judgement** |
| --- | --- | --- |
| Study participation | **High risk of bias**. The relationship between the  PF and outcome is very likely to be different for  participants and eligible nonparticipants. | Population source (people living in in a residential suburb) described. 5104 of 15974 eligible participatedi n baseline assessment. Period and place of recruitment described adequately. Inclusion and exclusion criteria given. Exclusion criteria were long-term care insurance system (care level >=3/5) disability in basic activities of daily living, and inability to undergo performance-based assessments. Participants and baseline described adequately. For longitudinal analysis participants with history of Parkinson´s disease, stroke, depression, Alzheimer’s disease, MMSE <18 a depression at bl were also excluded. As exclusion of disabled participants and participants with stroke may induce bias, we judged the bias to be high in this study. |
| Study Attrition | **Moderate risk of bias.** The relationship between the PF and outcome may be different for  completing and noncompleting participants. | 86% of baseline participants responded to 15-month follow-up survey. Information on reasons for loss to follow-up provided. No description of participants who dropped out given. |
| Prognostic Factor Measurement | **Low risk of bias.** The measurement of  the PF is unlikely to be different for  different levels of the outcome of interest. | PFs were clearly defined. Methods of measurement were valid and reliable. Continuous variables were reported. Method was the same for all study participants. As mentioned above, adequate proportion of study sample completed data for PFs. |
| Outcome measurement | **Low risk of bias.** The measurement of the  outcome is unlikely to be different related to  the baseline level of the PF. | Clear definition of outcome (GDS-15 >=6). Method valid and reliable. Method and setting of outcome measurement same for all study participants. |
| Study Confounding | **Moderate risk of bias**. The observed effect of the PF on outcome may be distorted.  by another factor related to  PF and outcome | All-important domains of potential confounders were accounted for in the statistical analysis. Measurement for Frailty was adequately valid and reliable. Measurements were the same for all study participants. |
| Statistical analysis and reporting | **Low risk of bias**. The reported results are unlikely to be spurious or biased related to analysis or reporting. | Sufficient presentation of data to assess the adequacy of the analytic strategy. Multiple logistic regression models were adequate for the design of the study. No signs for selective reporting were found. |

Mossaheb et al. 2009 (Mossaheb et al. 2009) Mossaheb, Nilufar; Weissgram, Silvia; Zehetmayer, Sonja; Jungwirth, Susanne; Rainer, Michael; Tragl, Karl-Heinz; Fischer, Peter (2009): Late-onset depression in elderly subjects from the Vienna Transdanube Aging (VITA) study. In: *The Journal of clinical psychiatry* 70 (4), S. 500–508. DOI: 10.4088/jcp.08m04265.

| **Risk of bias item** | **Author`s judgement** | **Support for judgement** |
| --- | --- | --- |
| Study participation | **Moderate risk of bias.** The relationship between the PF and outcome may be different for participants and eligible nonparticipants. | Source population of interest described. Participation rate 40% of eligible persons. Sampling frame and recruitment adequately describe, as well as period and place of recruitment described. Inclusion and exclusion criteria described. Baseline study sample not fully described. |
| Study Attrition | **Moderate risk of bias.** The relationship between the PF and outcome may be different for  completing and noncompleting participants. | 331 of 406 not demented and never depressed participants at bl fulfilled follow-up. Reasons for loss to follow-up provided. No description of participants lost to follow-up provided. Differences between participants who completed study and those who didn’t could therefore not be assessed. |
| Prognostic Factor Measurement | **High risk of bias.** The measurement of  the PF is very likely to be different for  different levels of the outcome of interest. | Not for all Prognostic factors a clear definition was provided. Measurement was therefore not always clearly valid or reliable. (e.g. „Close relatives were interviewed whenever possible “) |
| Outcome measurement | **High risk of bias**. The measurement of the  outcome is very likely to be different related  to the baseline level of the PF. | Current depressive episode diagnosed according to a questionnaire based on DSM-IV criteria for depressive episode, as well as Hamilton Rating Sclae for Depression and the Short Geriatric Depression scale. It was unclear, if participants had to fulfill criteria for depression in one, some or all of the instruments to be considered as depressed. In Addition, „current depressive episode was diagnosed by the same experienced geriatric psychologist according to a questionnaire based on DSM-IV criteria for depressive episode (…)“ which itself may increase risk of bias. |
| Study Confounding | **High risk of bias.** The observed effect of the PF  on the outcome is very likely to be distorted by another factor related to PF and outcome. | It was not clarified, exactly which confounders were accounted for in the analysis. |
| Statistical analysis and reporting | **High risk of bias.** The reported results are very  likely to be spurious or biased related to analysis or reporting | Adequacy of analytic strategy could not be assessed due to insufficient presentation of data to assess adequacy. |

Petersson et al. 2014 (Petersson et al. 2014) Petersson, Sofia; Mathillas, Johan; Wallin, Karin; Olofsson, Birgitta; Allard, Per; Gustafson, Yngve (2014): Risk factors for depressive disorders in very old age: a population-based cohort study with a 5-year follow-up. In: *Social psychiatry and psychiatric epidemiology* 49 (5), S. 831–839. DOI: 10.1007/s00127-013-0771-2

| **Risk of bias item** | **Author`s judgement** | **Support for judgement** |
| --- | --- | --- |
| Study participation | **Low risk of bias**. The relationship between the  PF and outcome is unlikely to be different for  participants and eligible nonparticipants. | Description of source population, sampling frame, recruitment and period and place of recruitment adequate. 567 of 831 eligible persons could be included into the baseline sample. Baseline sample described in detail. |
| Study Attrition | **Moderate risk of bias.** The relationship between the PF and outcome may be different for  completing and noncompleting participants. | 157 of 384 non-depressed participants at baseline completed Follow-up. Reasons for loss to follow-up provided. No description of participants lost to follow up provided. Therefore, differences between participants who completed study and those who did not could not be judged. |
| Prognostic Factor Measurement | **Low risk of bias**. The measurement of  the PF is unlikely to be different for different levels of the outcome of interest. | Clear definition of PFs provided. Methods valid and reliable. Continuous variables reported. Methods the same for all study participants. Adequate proportion has complete data for PF, as described above. |
| Outcome measurement | **High risk of bias.** The measurement of the  outcome is very likely to be different related  to the baseline level of the PF. | Any participants assessed in 2000–2001 were assessed with the MADRS during a complementary visit 2 weeks after initial assessment if GDS-15 was C5. In all later assessments MADRS was performed if the assessor was a physician or medical student trained in its use. Instruments were valid. However, method measurement therefore is not the same for all study participants and interviewers seemingly had different levels of training. |
| Study Confounding | **Low risk of bias.** The observed effect of the PF  on outcome is unlikely to be distorted by another factor related to PF and outcome. | Most important confounder, except alcohol consumption and drinking were assessed. Confounders were clearly defined. Method and setting for confounding measurement were the same for all study participants. Variables associated with new cases of depressive disorder with a p value\0.15, as well as age and gender, were included in a backward multivariate logistic regression analysis, after checking for collinearity. |
| Statistical analysis and reporting | **Moderate risk of bias.** The reported results may be spurious or biased related to analysis or reporting. | Statistical presentation of data sufficient to assess the adequacy of the analytic strategy. Selected statistical model was appropriate. There were hints of selective reporting in multivariate analysis, as not all results were reported. |

Schoevers et al 2005 (Schoevers et al. 2005) Schoevers, Robert A.; Deeg, D. J. H.; van Tilburg, W.; Beekman, A. T. F. (2005): Depression and generalized anxiety disorder: co-occurrence and longitudinal patterns in elderly patients. In: *The American journal of geriatric psychiatry : official journal of the American Association for Geriatric Psychiatry* 13 (1), S. 31–39. DOI: 10.1176/appi.ajgp.13.1.31.

| **Risk of bias item** | **Author`s judgement** | **Support for judgement** |
| --- | --- | --- |
| Study participation | **Moderate risk of bias.**  The relationship between the PF and outcome may be different for participants and eligible nonparticipants. | „All non-institutionalized individuals in the 65-84-year-old age bracket who lived in the city of Amsterdam.“ 71.5% of eligible subjects responded. Baseline sample described. Adequate description of sampling frame and recruitment. Inclusion and exclusion criteria adequately described. Participants with anxiety were excluded at baseline. |
| Study Attrition | **Moderate risk of bias.** The relationship between the PF and outcome may be different for  completing and noncompleting participants. | 55.4 5 were re-interviewed 3 years later. Reasons for loss to follow-up reported. Adequate description of participants lost to follow-up: „Non-response at the follow-up assessment was predicted by higher age, male gender, lower education level, chronic disease, ADL and IADL impairment, and organic caseness.  „. Therefore, differences between participants and participants lost to follow-up existed. |
| Prognostic Factor Measurement | **Low risk of bias**. The measurement of  the PF is unlikely to be different for  different levels of the outcome of interest. | Clear definition of PF was provided. The methods of measurement were valid and reliable. Appropriate cut-points were used. Methods were the same for all participants. Adequate proportion of sample completed data, as mentioned above. |
| Outcome measurement | **Low risk of bias.** The measurement of the  outcome is unlikely to be different related to the baseline level of the PF. | GMS-AGECAT >=3 was defined as depression. The method is valid and reliable. The method was used for all the study participants. |
| Study Confounding | **Moderate risk of bias.** The observed effect of the PF on outcome may be distorted by another factor related to PF and outcome. | Most potential confounders except alcohol consumption and smoking were measured with clear definitions and adequately valid and reliable measurements. These confounders were accounted for in the analysis and were the same for all study participants. |
| Statistical analysis and reporting | **Low risk of bias.** The reported results are unlikely  to be spurious or biased related to analysis or reporting. | Presentation of data was sufficient to assess adequacy of analytic strategy. The model was adequate. No hints to selective reporting. |

Schoevers et al. 2000 (Schoevers et al. 2000) Schoevers, R. A.; Beekman, A.T.F.; Deeg, D.J.H.; Geerlings, M. I.; Jonker, C.; van Tilburg, W. (2000): Risk factorsfor depression in later life; results of a prospective community based study (ASMTEL). In: *Journal of affective disorders*, S. 127–137.

| **Risk of bias item** | **Author`s judgement** | **Support for judgement** |
| --- | --- | --- |
| Study participation | **Moderate risk of bias.**  The relationship between the PF and outcome may be different for participants and eligible nonparticipants. | „All non-institutionalized individuals in the 65-84-year-old age bracket who lived in the city of Amsterdam. “ 71.5% of eligible subjects responded. Baseline sample described. Adequate description of sampling frame and recruitment. Inclusion and exclusion criteria adequately described. Participants with anxiety were excluded at baseline. |
| Study Attrition | **Moderate risk of bias.** The relationship between the PF and outcome may be different for  completing and noncompleting participants. | 55.4 5 were re-interviewed 3 years later. Reasons for loss to follow-up reported. Adequate description of participants lost to follow-up: „Non-response at the follow-up assessment was predicted by higher age, male gender, lower education level, chronic disease, ADL and IADL impairment, and organic caseness.  „. Therefore, differences between participants and participants lost to follow-up existed. (equal to Schoevers 2005) |
| Prognostic Factor Measurement | **Low risk of bias**. The measurement of  the PF is unlikely to be different for  different levels of the outcome of interest. | Clear definition of PF was provided. The methods of measurement were valid and reliable. Appropriate cut-points were used. Methods were the same for all participants. Adequate proportion of sample completed data, as mentioned above. (equal to Schoevers 2005) |
| Outcome measurement | **Low risk of bias.** The measurement of the  outcome is unlikely to be different related to the baseline level of the PF. | GMS-AGECAT >=3 was defined as depression. The method is valid and reliable. The method was used for all the study participants. (equal to Schoevers 2005) |
| Study Confounding | **Moderate risk of bias.** The observed effect of the PF on outcome may be distorted by another factor related to PF and outcome. | Most potential confounders except alcohol consumption and smoking were measured with clear definitions and adequately valid and reliable measurements. These confounders were accounted for in the stepwise logistic regression analysis and were the same for all study participants. |
| Statistical analysis and reporting | **Moderate risk of bias**. The reported results may be spurious or biased related to analysis or reporting. | Statistical model seemed adequate for the design of the study. Only significant variables in stepwise logistic regression were reported. Data on insignificant factors in stepwise logistic regression were not provided. |

Tani et al. 2016 (Tani et al. 2016) Tani, Yukako; Fujiwara, Takeo; Kondo, Naoki; Noma, Hisashi; Sasaki, Yuri; Kondo, Katsunori (2016): Childhood Socioeconomic Status and Onset of Depression among Japanese Older Adults: The JAGES Prospective Cohort Study. In: *The American Journal of Geriatric Psychiatry* 24 (9), S. 717–726. DOI: 10.1016/j.jagp.2016.06.001.

| **Risk of bias item** | **Author`s judgement** | **Support for judgement** |
| --- | --- | --- |
| Study participation | **Low risk of bias**. The relationship between the  PF and outcome is unlikely to be different for  participants and eligible nonparticipants. | Source population adequately described. Response rate was 65%. Baseline characteristics of participants were provided. Sampling frame and recruitment was described adequately. Inclusion and exclusion criteria provided |
| Study Attrition | **Moderate risk of bias.** The relationship between the PF and outcome may be different for  completing and noncompleting participants. | Response for follow-up was 80% of baseline participants. There was no detailed description on participants lost to follow up or the reasons for loss to follow up, only the description „exclusions of participants who had died, received benefits from public long-term care insurance, or moved to another municipality during the follow-up period”.  Therefore, differences between participants who completed the study and those lost to follow-up could not be judged. |
| Prognostic Factor Measurement | **Moderate risk of bias**. The measurement of  the PF may be different for different levels of the outcome of interest. | Prognostic factor „childhood socioeconomic status“ was clearly defined. Information about validity and reliability of this factor were not provided. Appropriate cut points were used. Measurement and setting was the same for all participants. Adequate proportion of study sample had complete data for PF, as mentioned above. |
| Outcome measurement | **Low risk of bias**. The measurement of the  outcome is unlikely to be different related to  the baseline level of the PF. | GDS-15 >= 6 as definition of depression was used for measurement of outcome for all study participants. The method is reliable and validated. |
| Study Confounding | **Moderate risk of bias.** The observed effect of the PF on outcome may be distorted by another factor related to PF and outcome. | Most important covariates were measured using clear definitions with valid and reliable methods of measurement. However, impairment was not accounted for in the analysis. |
| Statistical analysis and reporting | **Low risk of bias.** The reported results are unlikely  to be spurious or biased related to analysis or reporting. | Sufficient presentation of data to assess the adequacy of analytic strategy. Statistical model seemed adequate for the design of the study. No hints to selective reporting of results. |

Tsutsumoto et al. 2016 (Tsutsumimoto et al. 2017) Tsutsumimoto, Kota; Makizako, Hyuma; Doi, Takehiko; Hotta, Ryo; Nakakubo, Sho; Shimada, Hiroyuki; Suzuki, Takao (2017): Prospective associations between sedentary behaviour and incident depressive symptoms in older people: a 15-month longitudinal cohort study. In: *International journal of geriatric psychiatry* 32 (2), S. 193–200. DOI: 10.1002/gps.4461.

| **Risk of bias item** | **Author`s judgement** | **Support for judgement** |
| --- | --- | --- |
| Study participation | **High risk of bias**. The relationship between the  PF and outcome is very likely to be different for  participants and eligible nonparticipants. | Population source (people >= living in a residential suburb) described. 5104 of 15974 eligible participated n baseline assessment. Period and place of recruitment described adequately. Inclusion and exclusion criteria given. Exclusion criteria were long-term care insurance system (care level >=3/5) disability in basic activities of daily living, and inability to undergo performance-based assessments. Participants and baseline described adequately. For longitudinal analysis participants with history of Parkinson´s disease, stroke, depression, Alzheimer’s disease, MMSE <18 a depression at bl were also excluded. As exclusion of disabled participants and participants with stroke may induce bias, we judged the bias to be high in this study. |
| Study Attrition | **Moderate risk of bias.** The relationship between the PF and outcome may be different for  completing and noncompleting participants. | 86% of baseline participants responded to 15-month follow-up survey. Information on reasons for loss to follow-up provided. No description of participants who dropped out given. |
| Prognostic Factor Measurement | **Low risk of bias.** The measurement of  the PF is unlikely to be different for  different levels of the outcome of interest. | PFs were clearly defined. Methods of measurement were valid and reliable. Continuous variables were reported. Method was the same for all study participants. As mentioned above, adequate proportion of study sample completed data for PFs. |
| Outcome measurement | **Low risk of bias.** The measurement of the  outcome is unlikely to be different related to  the baseline level of the PF. | Clear definition of outcome (GDS-15 >=6). Method valid and reliable. Method and setting of outcome measurement same for all study participants. |
| Study Confounding | **High risk of bias.** The observed effect of the PF  on the outcome is very likely to be distorted by another factor related to PF and outcome. | Most potential confounders were clearly defined, measured with valid and reliable instruments and were the same for all participants of the study. However marital status, physical comorbidities and impairment were not accounted for in the analysis. As mobility parameters were examined as factors, we judged the risk of bias to be high. |
| Statistical analysis and reporting | **Low risk of bias**. The reported results are unlikely  to be spurious or biased  related to analysis or reporting | Sufficient presentation of data to assess the adequacy of the analytic strategy. Selected model was appropriate fort the study design. We found no hints to selective reporting of results. |

Uemura et al. 2018(Uemura et al. 2018) Uemura, Kazuki; Makizako, Hyuma; Lee, Sangyoon; Doi, Takehiko; Lee, Songchul; Tsutsumimoto, Kota; Shimada, Hiroyuki (2018): Behavioral protective factors of increased depressive symptoms in community-dwelling older adults: A prospective cohort study. In: *Int J Geriatr Psychiatry* 33 (2), e234-e241. DOI: 10.1002/gps.4776.

| **Risk of bias item** | **Author`s judgement** | **Support for judgement** |
| --- | --- | --- |
| Study participation | **High risk of bias**. The relationship between the  PF and outcome is very likely to be different for  participants and eligible nonparticipants. | Population source (people >= living in a residential suburb) described. 5104 of 15974 eligible participated n baseline assessment. Period and place of recruitment described adequately. Inclusion and exclusion criteria given. Exclusion criteria were long-term care insurance system (care level >=3/5) disability in basic activities of daily living, and inability to undergo performance-based assessments. Participants and baseline described adequately. For longitudinal analysis participants with history of Parkinson´s disease, stroke, depression, Alzheimer’s disease, MMSE <18 a depression at bl were also excluded. As exclusion of disabled participants and participants with stroke may induce bias, we judged the bias to be high in this study. |
| Study Attrition | **Moderate risk of bias.** The relationship between the PF and outcome may be different for  completing and noncompleting participants. | 86% of baseline participants responded to 15-month follow-up survey. Information on reasons for loss to follow-up provided. No description of participants who dropped out given. |
| Prognostic Factor Measurement | **Low risk of bias.** The measurement of  the PF is unlikely to be different for  different levels of the outcome of interest. | PFs were clearly defined. Methods of measurement were valid and reliable. Continuous variables were reported. Method was the same for all study participants. As mentioned above, adequate proportion of study sample completed data for PFs. |
| Outcome measurement | **Low risk of bias.** The measurement of the  outcome is unlikely to be different related to  the baseline level of the PF. | Clear definition of outcome (GDS-15 >=6). Method valid and reliable. Method and setting of outcome measurement same for all study participants. |
| Study Confounding | **Low risk of bias**. The observed effect of the PF  on outcome is unlikely to be distorted by another factor related to PF and outcome. | All-important confounders were clearly defined, measured with adequately valid and reliable instruments and were the same for all study participants. All important confounders were accounted for in the analysis. |
| Statistical analysis and reporting | **Low risk of bias**. The reported results are unlikely  to be spurious or biased  related to analysis or reporting | Sufficient presentation of data to assess the adequacy of the analytic strategy was provided. The selected model was adequate for the design. We found no signs for selective reporting of results. |

Weyerer et al. 2013 (Weyerer et al. 2013) Weyerer, Siegfried; Eifflaender-Gorfer, Sandra; Wiese, Brigitte; Luppa, Melanie; Pentzek, Michael; Bickel, Horst et al. (2013): Incidence and predictors of depression in non-demented primary care attenders aged 75 years and older: results from a 3-year follow-up study. In: *Age and ageing* 42 (2), S. 173–180. DOI: 10.1093/ageing/afs184.

| **Risk of bias item** | **Author`s judgement** | **Support for judgement** |
| --- | --- | --- |
| Study participation | **Low risk of bias**. The relationship between the  PF and outcome is unlikely to be different for  participants and eligible nonparticipants. | 3292 of 6619 randomly selected persons completed the baseline assessment. Source and population of interest described in detail. Sampling frame, period and place of recruitment described in detail. Inclusion and exclusion criteria adequate. Description of baseline sample provided. |
| Study Attrition | **Moderate risk of bias**. The relationship between the PF and outcome is unlikely to be different.  for completing and noncompleting participants. | Attrition rate during 3-year follow-up period was 13.7%. Reasons for loss to follow-up provided. No description of participants lost to follow-up provided. Therefore, differences between participants who completed the study and those who did not could not be judged. |
| Prognostic Factor Measurement | **Low risk of bias**. The measurement of  the PFs is unlikely to be different for  different levels of the outcome of interest. | Prognostic factors were clearly defined, method of measurements were adequately valid and reliable, appropriate cut points were used and the methods were the same for all study participants. As mentioned above, adequate proportion of the study sample hat complete data for the PFs. |
| Outcome measurement | **Low risk of bias**. The measurement of the outcome is unlikely to be different related to the baseline level of the PF. | Outcome measurement clearly defined, valid and reliable. Same method was used for all study participants. |
| Study Confounding | **Low risk of bias.** The observed effect of the PF  on outcome is unlikely to be distorted by another factor related to PF and outcome. | All important potential confounders were measured, clearly defined, assessed with valid and reliable instruments and were the same for all study participants. These confounders were accounted for in the analysis. |
| Statistical analysis and reporting | **Low risk of bias.** The reported results are unlikely  to be spurious or biased related to analysis or reporting. | Sufficient presentation of data to assess the adequacy of the analytic strategy was provided. The selected model was adequate for the design. We found no signs for selective reporting of results. |

Yang et al. 2015 (Yang et al. 2015) Yang, Ya-Ting; Wang, Yao-Hsien; Chiu, Hsien-Tsai; Wu, Chia-Rong; Handa, Yujiro; Liao, Yin-Lin; Hsu, Yi-Hsin Elsa (2015): Functional limitations and somatic diseases are independent predictors for incident depressive disorders in seniors: Findings from a nationwide longitudinal study. In: *Archives of Gerontology and Geriatrics* 61 (3), S. 371–377. DOI: 10.1016/j.archger.2015.07.005.

| **Risk of bias item** | **Author`s judgement** | **Support for judgement** |
| --- | --- | --- |
| Study participation | **Moderate risk of bias.** The relationship between the PF and outcome may be  different for participants and eligible nonparticipants. | Source population described. Sampling frame and recruitment described. Description of baseline characteristics of participants provided. Inclusion and exclusion criteria described. No information about response rates provided. |
| Study Attrition | **High risk of bias.** The relationship between the PF and outcome is very likely to be different for completing and noncompleting participants. | 1467 of 5377 participants at baseline completed follow up. Reasons for loss to follow-up not prvided. No information about participants who dropped out provided. Differences between participants who completed study and those who did not could therefore not be judged. |
| Prognostic Factor Measurement | **Low risk of bias**. The measurement of  the PF is unlikely to be different for different levels of the outcome of interest. | Clear definition of prognostic factors was provided. Methods were adequately valid, reliable, used appropriate cut points and the same methods were used for all study participants. |
| Outcome measurement | **Low risk of bias**. The measurement of the  outcome is unlikely to be different related to the baseline level of the PFs. | CES-D-10 score >=10 was defined as depression. Method is valid and reliable. Method was same for all study participants. |
| Study Confounding | **Moderate risk of bias**. The observed effect of the PF on outcome may be distorted by another factor related to PF and outcome. | Most potential confounders were clearly defined, measured with valid and reliable instruments and were the same for all participants of the study. However, only change of impairment, not impairment at baseline were accounted for in the analysis. |
| Statistical analysis and reporting | **Low risk of bias**. The reported results are unlikely  to be spurious or biased related to analysis or reporting. | Sufficient presentation of data to assess the adequacy of the analytic strategy was provided. The selected model was adequate for the design. We found no signs for selective reporting of results. |

Yokohama et al. 2010 (Yokoyama et al. 2010) Yokoyama, Eise; Kaneita, Yoshitaka; Saito, Yasuhiko; Uchiyama, Makoto; Matsuzaki, Yoko; Tamaki, Tetsuo et al. (2010): Association between depression and insomnia subtypes: a longitudinal study on the elderly in Japan. In: *Sleep* 33 (12), S. 1693–1702. DOI: 10.1093/sleep/33.12.1693

| **Risk of bias item** | **Author`s judgement** | **Support for judgement** |
| --- | --- | --- |
| Study participation | **Moderate risk of bias**. The relationship between the PF and outcome may be different for participants and eligible nonparticipants. | 74.6 % retrieval. Sampling frame and place of recruitment not described in detail. Period of recruitment provided. Inclusion and exclusion criteria adequately described. Description of baseline sample provided. |
| Study Attrition | **High risk of bias.** The relationship between the PF and outcome is very likely to be different for completing and noncompleting participants | 3065 of 4028 without depression at baseline were included in longitudinal analysis. No exact information on reasons for loss to follow-up provided. No adequate description of participants lost to follow- up. Differences between participants who completed the study and those who did not could not be judged. |
| Prognostic Factor Measurement | **Moderate risk of bias.** The measurement of the PF may be different for different levels of the outcome of interest | All prognostic factors were clearly defined. No information about validity and reliability of self-reported questions about sleep were provided. Appropriate cut points were used. Measurements for PFs was the same for all study participants. Adequate proportion of sample had complete data for PFs (as mentioned above). |
| Outcome measurement | **Low risk of bias.** The measurement of the outcome is unlikely to be different related to the baseline level of the PF | CES-D 11 with cut- off >= 6/7. Method is valid and reliable. It was used for all study participants. |
| Study Confounding | **Moderate risk of bias**. The observed effect of the PF on outcome may be distorted by another factor related to PF and outcome | Most important confounders were measured, clearly defined with valid and reliable measurements, and were accounted for in the analysis. However, Smoking, Alcohol consumption, marital status and somatic comorbidity, as well as self-rated health were not accounted for in the analysis |
| Statistical analysis and reporting | **Low risk of bias.** The reported results are unlikely to be spurious or biased related to analysis or reporting | Statistical model was adequate for design of study. Sufficient presentation of data to assess adequacy of analytic strategy. We found no hints for selective reporting. |

Misawa et al. (Misawa und Kondo) Misawa, Jimpei; Kondo, Katsunori: Social factors relating to depression among older people in Japan: analysis of longitudinal panel date from the AGES project. In: *Aging & mental health* (10), S. 1423–1432.

| **Risk of bias item** | **Author`s judgement** | **Support for judgement** |
| --- | --- | --- |
| Study participation | **High risk of bias.** The relationship between the PF and outcome is very likely to be different for participants and eligible nonparticipants | Population of interest adequately described. Response rate was 49%. Descriptive statistics of baseline sample provided. Sampling frame and recruitment methods are described adequately. Inclusion and exclusion criteria provided. Individuals with physical and cognitive disabilities were excluded. Excluding persons with cognitive disabilities is adequate, however excluding persons with disabilities represents a high risk of bias. |
| Study Attrition | **Moderate risk of bias.** The relationship between the PF and outcome may be different for completing and noncompleting participants | Follow-up rate was 63.5%. Information about reasons for loss to follow-up provided. No adequate description of participants lost to follow-up was provided. Differences between participants who completed the study and those lost to follow-up therefore could not be judged. |
| Prognostic Factor Measurement | **Moderate risk of bias**. The measurement of the PF may be different for different levels of the outcome of interest | Clear definition of risk factors was provided. Method of measurement for most risk factors were valid and reliable. Measurement of Emotional and Instrumental support were measured with single questions and no information about validity and reliability for this method was provided. Methods were the same for all participants. Appropriate cut points were used. Adequate proportion of sample had complete data, as mentioned above. |
| Outcome measurement | **Low risk of bias**. The measurement of the outcome is unlikely to be different related to the baseline level of the PF | GDS-15 with >=5 was defined as depression. Method is valid and reliable. Method was the same for all participants. |
| Study Confounding | **Moderate risk of bias.** The observed effect of the PF on outcome may be distorted by another factor related to PF and outcome | Most important potential confounders, except for alcohol consumption and smoking were clearly defined, measured with valid and reliable methods and were the same for all participants. All these confounders were accounted for in the analysis. Results were reported for women and men separately. |
| Statistical analysis and reporting | **Low risk of bias.** The reported results are unlikely to be spurious or biased related to analysis or reporting | Sufficient data was provided to assess adequacy of the analytics strategy. Selected model was adequate for design of the study. We found no hints to selective reporting. |

Ryan et al. 2015 (Ryan et al. 2015) Ryan, Joanne; Carrière, Isabelle; Ritchie, Karen; Ancelin, Marie-Laure (2015): Involvement of GPR50 polymorphisms in depression: independent replication in a prospective elderly cohort. In: *Brain and behavior* 5 (3), e00313. DOI: 10.1002/brb3.313.

| **Risk of bias item** | **Author`s judgement** | **Support for judgement** |
| --- | --- | --- |
| Study participation | **Moderate risk of bias.** The relationship between the PF and outcome may be different for participants and eligible nonparticipants | Source population adequately described. Participation rate of eligible persons not provided. Descriptive statistics of baseline study sample provided. Recruitment procedure and place of recruitment were not fully provided. Inclusion and exclusion criteria were described. „Participants (..) were less likely to be depressed, use antidepressants and had fewer incapacities, cognitive impairment and cardiovascular disease,(..), but were more likely to live with other and be better educated.“ |
| Study Attrition | **High risk of bias**. The relationship between the PF and outcome is very likely to be different for completing and noncompleting participants | Response rate for follow-ups was not reported. No information about reasons for loss to follow-up or any description of participants lost to follow-up was provided. Difference between participants who completed the study and those who did not could therefore not be judged. |
| Prognostic Factor Measurement | **Moderate risk of bias.** The measurement of the PF may be different for different levels of the outcome of interest | Clear definition of the risk factor was provided. Method seemed to be valid and reliable. Method was used for all participants. Appropriate cut points were used. No information about the proportion of participants with full information about RF. |
| Outcome measurement | **Low risk of bias.** The measurement of the PF is unlikely to be different for different levels of the outcome of interest | CES-D-10 with cut-off at 16 or current diagnosis of MDD was defined as depression. Method is valid and reliable and was used for all study participants. |
| Study Confounding | **Moderate risk of bias.** The observed effect of the PF on outcome may be distorted by another factor related to PF and outcome | Many important confounders were not accounted for in the analysis: marital status: alcohol consumption, smoking, or self rated health were not accounted for in the analysis. The assessed confounders were measured with instruments adequately valid and reliable and were the same for all participants. |
| Statistical analysis and reporting | **Low risk of bias.** The reported results are unlikely to be spurious or biased related to analysis or reporting | Sufficient presentation of data to assess adequacy of analytic strategy. Selected model was adequate. For the risk factor GPR50 polymorphism analysing factors for men and women separately was necessary and adequate. We found no clear signs of selective reporting. |

Ojagbemi et al. 2018 (Ojagbemi et al. 2018) Ojagbemi, Akin; Bello, Toyin; Gureje, Oye (2018): Gender differential in social and economic predictors of incident major depressive disorder in the Ibadan Study of Ageing. In: *Social psychiatry and psychiatric epidemiology* 53 (4), S. 351–361. DOI: 10.1007/s00127-018-1500-7.

| **Risk of bias item** | **Author`s judgement** | **Support for judgement** |
| --- | --- | --- |
| Study participation | **Low risk of bias.** The relationship between the  PF and outcome is unlikely to be different for  participants and eligible nonparticipants. | Source population in 8 regions and multi-stage random sampling of households described. Sampling frame and description of period and place adequate. Inclusion and exclusion criteria described. Description of baseline sample provided (for men and women separately. |
| Study Attrition | **Moderate risk of bias.** The relationship between the PF and outcome may be different for completing and noncompleting participants | Response rate from baseline to follow-u 65.5%, adequate. Reasons for loss to follow-up provided. Participants not lost-to follow-up were least likely to belong to lowest economic class. No difference in sex, residence, educational level or presence of absence of subsyndromal depression. |
| Prognostic Factor Measurement | **Low risk of bias**. The measurement of  the PF is unlikely to be different for different levels of the outcome of interest. | RF clearly defined, and adequately valid and reliable according to authors. Adequate cut points are used. Methods are the same for all participants. Adequate proportion of sample had complete data for RF, as mentioned above. |
| Outcome measurement | **Low risk of bias.** The measurement of the  outcome is unlikely to be different related to  the baseline level of the RF. | Definition of depression as outcome provided (DSM-IV-criteria for MDD). Method valid and reliable. Method the same for all study participants. |
| Study Confounding | **High risk of bias.** The observed effect of the PF  on the outcome is very likely to be distorted by another factor related to PF and outcome. | Important risk factors, such as impairment, alcohol consumption, smoking, self rated health or somatic comorbidity were not accounted for in the analysis. |
| Statistical analysis and reporting | **Low risk of bias.** The reported results are unlikely.  to be spurious or biased related to analysis or reporting. | Sufficient presentation of data to assess the adequacy of the analytic strategy. Statistic model adequate. No clear signs of selective reporting. |

Carrière et al. 2013 (Carrière et al. 2013) Carrière, Isabelle; Delcourt, Cécile; Daien, Vincent; Pérès, Karine; Féart, Catherine; Berr, Claudine et al. (2013): A prospective study of the bi-directional association between vision loss and depression in the elderly. In: *Journal of affective disorders* 151 (1), S. 164–170. DOI: 10.1016/j.jad.2013.05.071.

| **Risk of bias item** | **Author`s judgement** | **Support for judgement** |
| --- | --- | --- |
| Study participation | **Moderate risk of bias**. The relationship between the  PF and outcome may be different for participants.  and eligible nonparticipants. | No information about particpation rate in this study. Source population described. Descriptive statistics of baseline sample provided. Sampling frame and recruitment, as well as period and place of recruitment described. Inclusion and exclusion criteria described. Persons using antidepressants excluded, as antidepressants are also prescribed in anxiety or obsessive-compulsive disorder. |
| Study Attrition | **Moderate risk of bias.** The relationship between the PF and outcome may be different for  completing and noncompleting participants. | No information about response rates provided. Reasons for loss to follow-up were reported. No description of participants lost to follow up was provided. Therefore, differences between participants who completed the study and those who did not could not be judged. |
| Prognostic Factor Measurement | **Low risk of bias**. The measurement of  the PF is unlikely to be different for  different levels of the outcome of interest. | Clear definition of RFs provided. Methods of measurement had adequate validity and reliability. For continuous variables adequate cut points were used. Same methods were used for all participants. |
| Outcome measurement | **Low risk of bias.** The measurement of the  outcome is unlikely to be different related to  the baseline level of the PF. | Outcome clearly defined (current Major depressive episode according to DSM-IV or CES-D-20- score >=16). Methods valid and reliable. Methods were the same for all study participants. |
| Study Confounding | **High risk of bias.** The observed effect of the PF  on the outcome is very likely to be distorted by another factor related to PF and outcome. | Important potential confounders such as marital status, alcohol consumption, ADL or IADL impairment were not accounted for in the analysis. As visual impairment is analyzed as a risk factor, not accounting for other types of impairment as potential confounders represents a high risk of bias. |
| Statistical analysis and reporting | **Low risk of bias.** The reported results are unlikely.  to be spurious or biased related to analysis or reporting | Sufficient presentation of data to assess adequacy of the analytic strategy. Selected statistical model adequate. No signs for selective reporting. |

Ancelin et al. 2010 (Ancelin et al. 2010) Ancelin, Marie-Laure; Carrière, Isabelle; Boulenger, Jean-Philippe; Malafosse, Alain; Stewart, Robert; Cristol, Jean-Paul et al. (2010): Gender and genotype modulation of the association between lipid levels and depressive symptomatology in community-dwelling elderly (the ESPRIT study). In: *Biological psychiatry* 68 (2), S. 125–132. DOI: 10.1016/j.biopsych.2010.04.011.

| **Risk of bias item** | **Author`s judgement** | **Support for judgement** |
| --- | --- | --- |
| Study participation | **Low risk of bias.** The relationship between the  PF and outcome is unlikely to be different for  participants and eligible nonparticipants. | Participation rate was 27.3%. Population of interest was described. Sampling frame, recruitment, period, and place of recruitment adequately described. Inclusion and exclusion criteria described. |
| Study Attrition | **Moderate risk of bias.** The relationship between the PF and outcome may be different for  completing and noncompleting participants. | No information about response rates provided. Reasons for loss to follow-up were reported. No description of participants lost to follow up was provided. Therefore, differences between participants who completed the study and those who did not couldn´t be judged. (information in other study of the same sample.) |
| Prognostic Factor Measurement | **Moderate risk of bias**. The measurement of  the PF may be different for different levels of the outcome of interest. | Risk factors clearly defined. Measurement was valid and reliable for most risk factors. However, „participants interviewed at home were asked to show medical prescriptions, drug packages, and any other relevant information; those interviewed at the study center were asked to come with their prescription forms “. This may have led to reporting bias in the case of this specific factor. |
| Outcome measurement | **Low risk of bias.** The measurement of the  outcome is unlikely to be different related to  the baseline level of the PF. | Outcome clearly defined (current Major depressive episode according to DSM-IV or CES-D-20- score >=16). Methods valid and reliable. Methods were the same for all study participants. |
| Study Confounding | **Moderate risk of bias.** The observed effect of the PF on outcome may be distorted by another factor related to PF and outcome. | Most potential confounders were measured, clear definitions were provided, all important confounders were adequately valid and reliable and accounted for in the analysis. However, impairment was not accounted for as a confounder in the analysis. |
| Statistical analysis and reporting | **Low risk of bias.** The reported results are unlikely  to be spurious or biased related to analysis or reporting. | Sufficient presentation of data to assess adequacy of the analytic strategy. Selected statistical model adequate. No signs for selective reporting. |

Literaturverzeichnis

Ancelin, Marie-Laure; Carrière, Isabelle; Boulenger, Jean-Philippe; Malafosse, Alain; Stewart, Robert; Cristol, Jean-Paul et al. (2010): Gender and genotype modulation of the association between lipid levels and depressive symptomatology in community-dwelling elderly (the ESPRIT study). In: *Biological psychiatry* 68 (2), S. 125–132. DOI: 10.1016/j.biopsych.2010.04.011.

Carrière, Isabelle; Delcourt, Cécile; Daien, Vincent; Pérès, Karine; Féart, Catherine; Berr, Claudine et al. (2013): A prospective study of the bi-directional association between vision loss and depression in the elderly. In: *Journal of affective disorders* 151 (1), S. 164–170. DOI: 10.1016/j.jad.2013.05.071.

Chou, Kee-Lee (2007a): Combined effect of vision and hearing impairment on depression in older adults: Evidence from the English Longitudinal Study of the Ageing. In: *Journal of affective disorders*, S. 191–196.

Chou, Kee-Lee (2007b): Reciprocal relationship between pain and depression in older adults: evidence from the English Longitudinal Study of Ageing. In: *Journal of affective disorders* 102 (1-3), S. 115–123. DOI: 10.1016/j.jad.2006.12.013.

Dong, Yutong; Yang, Frances Margaret (2019): Insomnia symptoms predict both future hypertension and depression. In: *Preventive Medicine* 123, S. 41–47. DOI: 10.1016/j.ypmed.2019.02.001.

Forsell, Y. (2000): Y. Forsell Predictors for Depression, Anxiety and psychotic Predictors for Depression, Anxiety and psychotic symptoms in a very elderly population: data from a 3-year follow-up study. In: *Social psychiatry and psychiatric epidemiology* 2000 (35), S. 259–263. Online verfügbar unter https://link.springer.com/content/pdf/10.1007%2Fs001270050237.pdf, zuletzt geprüft am 08.10.2019.

Gureje, O.; Oladeji, B.; Abiona, T. (2011): Incidence and risk factors for late-life depression in the Ibadan Study of Ageing. In: *Psychological medicine* 41 (9), S. 1897–1906. DOI: 10.1017/S0033291710002643.

Hayden, Jill A.; van der Windt, Danielle A.; Cartwright, Jennifer L.; Côté, Pierre; Bombardier, Claire (2013): Assessing bias in studies of prognostic factors. In: *Annals of internal medicine* 158 (4), S. 280–286. DOI: 10.7326/0003-4819-158-4-201302190-00009.

Kim, Jae-Min; Stewart, Robert; Kim, Sung-Wan; Yang, Su-Jin; Shin, Il-Seon; Yoon, Jin-Sang (2006): Vascular risk factors and incident late-life depression in a Korean population. In: *The British journal of psychiatry : the journal of mental science* 189, S. 26–30. DOI: 10.1192/bjp.bp.105.015032.

Kim, Jae-Min; Stewart, Robert; Kim, Sung-Wan; Yang, Su-Jin; Shin, Il-Seon; Yoon, Jin-Sang (2009): Insomnia, depression, and physical disorders in late life: a 2-year longitudinal community study in Koreans. In: *Sleep* 32 (9), S. 1221–1228. DOI: 10.1093/sleep/32.9.1221.

Koizumi, Yayoi; Awata, Shuichi; Kuriyama, Shinichi; Ohmori, Kaori; Hozawa, Atsushi; Seki, Toru et al. (2005): Association between social support and depression status in the elderly: results of a 1-year community-based prospective cohort study in Japan. In: *Psychiatry and clinical neurosciences* 59 (5), S. 563–569. DOI: 10.1111/j.1440-1819.2005.01415.x.

Lampinen, Päivi; Heikkinen, Eino (2003): Reduced mobility and physical activity as predictors of depressive symptoms among community-dwelling older adults: an eight-year follow-up study. In: *Aging clinical and experimental research* 15 (3), S. 205–211.

Lue, Bee-Horng; Liang-Ju, Chen; Shwu-Chong, Wu (2010): Health, financial stresses, and life satisfaction affecting late-life depression among older adults: a nationwide, longitudinal survey in Taiwan. In: *Archives of Gerontology and Geriatrics* 2010 (50), S. 34–38.

Luppa, Melanie; Luck, Tobias; König, Hans-Helmut; Angermeyer, Matthias C.; Riedel-Heller, Steffi G. (2012): Natural course of depressive symptoms in late life. An 8-year population-based prospective study. In: *Journal of affective disorders* 142 (1-3), S. 166–171. DOI: 10.1016/j.jad.2012.05.009.

Lyness, Jeffrey M.; Yu, Qin; Tang, Wan; Tu, Xin; Conwell, Yeates (2009): Risks for depression onset in primary care elderly patients: potential targets for preventive interventions. In: *The American journal of psychiatry* 166 (12), S. 1375–1383. DOI: 10.1176/appi.ajp.2009.08101489.

Makizako, Hyuma; Shimada, Hiroyuki; Doi, Takehiko; Yoshida, Daisuke; Anan, Yuya; Tsutsumimoto, Kota et al. (2015): Physical frailty predicts incident depressive symptoms in elderly people: prospective findings from the Obu Study of Health Promotion for the Elderly. In: *Journal of the American Medical Directors Association* 16 (3), S. 194–199. DOI: 10.1016/j.jamda.2014.08.017.

Misawa, Jimpei; Kondo, Katsunori: Social factors relating to depression among older people in Japan: analysis of longitudinal panel date from the AGES project. In: *Aging & mental health* (10), S. 1423–1432.

Mossaheb, Nilufar; Weissgram, Silvia; Zehetmayer, Sonja; Jungwirth, Susanne; Rainer, Michael; Tragl, Karl-Heinz; Fischer, Peter (2009): Late-onset depression in elderly subjects from the Vienna Transdanube Aging (VITA) study. In: *The Journal of clinical psychiatry* 70 (4), S. 500–508. DOI: 10.4088/jcp.08m04265.

Ojagbemi, Akin; Bello, Toyin; Gureje, Oye (2018): Gender differential in social and economic predictors of incident major depressive disorder in the Ibadan Study of Ageing. In: *Social psychiatry and psychiatric epidemiology* 53 (4), S. 351–361. DOI: 10.1007/s00127-018-1500-7.

Petersson, Sofia; Mathillas, Johan; Wallin, Karin; Olofsson, Birgitta; Allard, Per; Gustafson, Yngve (2014): Risk factors for depressive disorders in very old age: a population-based cohort study with a 5-year follow-up. In: *Social psychiatry and psychiatric epidemiology* 49 (5), S. 831–839. DOI: 10.1007/s00127-013-0771-2.

Ryan, Joanne; Carrière, Isabelle; Ritchie, Karen; Ancelin, Marie-Laure (2015): Involvement of GPR50 polymorphisms in depression: independent replication in a prospective elderly cohort. In: *Brain and behavior* 5 (3), e00313. DOI: 10.1002/brb3.313.

Schoevers, R. A.; Beekman, A.T.F.; Deeg, D.J.H.; Geerlings, M. I.; Jonker, C.; van Tilburg, W. (2000): Risk factorsfor depression in later life; results of a prospective community based study (ASMTEL). In: *Journal of affective disorders*, S. 127–137.

Schoevers, Robert A.; Deeg, D. J. H.; van Tilburg, W.; Beekman, A. T. F. (2005): Depression and generalized anxiety disorder: co-occurrence and longitudinal patterns in elderly patients. In: *The American journal of geriatric psychiatry : official journal of the American Association for Geriatric Psychiatry* 13 (1), S. 31–39. DOI: 10.1176/appi.ajgp.13.1.31.

Tani, Yukako; Fujiwara, Takeo; Kondo, Naoki; Noma, Hisashi; Sasaki, Yuri; Kondo, Katsunori (2016): Childhood Socioeconomic Status and Onset of Depression among Japanese Older Adults: The JAGES Prospective Cohort Study. In: *The American Journal of Geriatric Psychiatry* 24 (9), S. 717–726. DOI: 10.1016/j.jagp.2016.06.001.

Tsutsumimoto, Kota; Makizako, Hyuma; Doi, Takehiko; Hotta, Ryo; Nakakubo, Sho; Shimada, Hiroyuki; Suzuki, Takao (2017): Prospective associations between sedentary behaviour and incident depressive symptoms in older people: a 15-month longitudinal cohort study. In: *International journal of geriatric psychiatry* 32 (2), S. 193–200. DOI: 10.1002/gps.4461.

Uemura, Kazuki; Makizako, Hyuma; Lee, Sangyoon; Doi, Takehiko; Lee, Songchul; Tsutsumimoto, Kota; Shimada, Hiroyuki (2018): Behavioral protective factors of increased depressive symptoms in community-dwelling older adults: A prospective cohort study. In: *Int J Geriatr Psychiatry* 33 (2), e234-e241. DOI: 10.1002/gps.4776.

Weyerer, Siegfried; Eifflaender-Gorfer, Sandra; Wiese, Birgitt; Luppa, Melanie; Pentzek, Michael; Bickel, Horst et al. (2013): Incidence and predictors of depression in non-demented primary care attenders aged 75 years and older: results from a 3-year follow-up study. In: *Age and ageing* 42 (2), S. 173–180. DOI: 10.1093/ageing/afs184.

Yang, Ya-Ting; Wang, Yao-Hsien; Chiu, Hsien-Tsai; Wu, Chia-Rong; Handa, Yujiro; Liao, Yin-Lin; Hsu, Yi-Hsin Elsa (2015): Functional limitations and somatic diseases are independent predictors for incident depressive disorders in seniors: Findings from a nationwide longitudinal study. In: *Archives of Gerontology and Geriatrics* 61 (3), S. 371–377. DOI: 10.1016/j.archger.2015.07.005.

Yokoyama, Eise; Kaneita, Yoshitaka; Saito, Yasuhiko; Uchiyama, Makoto; Matsuzaki, Yoko; Tamaki, Tetsuo et al. (2010): Association between depression and insomnia subtypes: a longitudinal study on the elderly in Japan. In: *Sleep* 33 (12), S. 1693–1702. DOI: 10.1093/sleep/33.12.1693.
